# Novel Polymyxin-Inspired Peptidomimetics Targeting the SARS-CoV-2 Spike:hACE2 Interface

**DOI:** 10.3390/ijms24108765

**Published:** 2023-05-15

**Authors:** Kelly Bugatti, Andrea Sartori, Lucia Battistini, Crescenzo Coppa, Emiel Vanhulle, Sam Noppen, Becky Provinciael, Lieve Naesens, Annelies Stevaert, Alessandro Contini, Kurt Vermeire, Franca Zanardi

**Affiliations:** 1 Department of Food and Drug, University of Parma, Parco Area delle Scienze 27/A, 43124 Parma, Italy; kelly.bugatti@unipr.it (K.B.); andrea.sartori@unipr.it (A.S.); lucia.battistini@unipr.it (L.B.); 2 Department of Pharmaceutical Sciences, University of Milan, Via Venezian 21, 20133 Milano, Italy; crescenzo.coppa@unimi.it; 3 KU Leuven, Department of Microbiology, Immunology and Transplantation, Rega Institute, Laboratory of Virology and Chemotherapy, Herestraat 49, 3000 Leuven, Belgium; emiel.vanhulle@kuleuven.be (E.V.); sam.noppen@kuleuven.be (S.N.); becky.provinciael@kuleuven.be (B.P.); lieve.naesens@kuleuven.be (L.N.); annelies.stevaert@kuleuven.be (A.S.); kurt.vermeire@kuleuven.be (K.V.)

**Keywords:** cyclic peptides, protein–peptide interactions, antiviral agents, drug design, receptor-binding domain (RBD), solid-phase peptide synthesis, polymyxin-like peptidomimetics

## Abstract

Though the bulk of the COVID-19 pandemic is behind, the search for effective and safe anti-SARS-CoV-2 drugs continues to be relevant. A highly pursued approach for antiviral drug development involves targeting the viral spike (S) protein of SARS-CoV-2 to prevent its attachment to the cellular receptor ACE2. Here, we exploited the core structure of polymyxin B, a naturally occurring antibiotic, to design and synthesize unprecedented peptidomimetics (PMs), intended to target contemporarily two defined, non-overlapping regions of the S receptor-binding domain (RBD). Monomers **1**, **2**, and **8**, and heterodimers **7** and **10** bound to the S-RBD with micromolar affinity in cell-free surface plasmon resonance assays (*K*_D_ ranging from 2.31 μM to 2.78 μM for dimers and 8.56 μM to 10.12 μM for monomers). Although the PMs were not able to fully protect cell cultures from infection with authentic live SARS-CoV-2, dimer **10** exerted a minimal but detectable inhibition of SARS-CoV-2 entry in U87.ACE2^+^ and A549.ACE2.TMPRSS2^+^ cells. These results validated a previous modeling study and provided the first proof-of-feasibility of using medium-sized heterodimeric PMs for targeting the S-RBD. Thus, heterodimers **7** and **10** may serve as a lead for the development of optimized compounds, which are structurally related to polymyxin, with improved S-RBD affinity and anti-SARS-CoV-2 potential.

## 1. Introduction

Three years have passed since the beginning of the Corona Virus Disease COVID-19 pandemic caused by Severe Acute Respiratory Syndrome Coronavirus 2 (SARS-CoV-2), which left hundreds of millions of people infected, while claiming millions of victims and causing heavy economic and social burdens worldwide [1,2,3]. Widespread vaccination and current clinical management have strongly reduced both the mortality and severity of the disease [1,2,4]; however, serious concerns remain, including the following: the rise of viral resistance and reduced sensitivity to vaccines [5,6]; difficulties in effectively protecting vulnerable segments of the population, including the elderly and patients with multiple comorbidities [7,8,9]; and the unclear mechanisms of chronic-lasting effects of the disease observed in a percentage of infected people (long-COVID effects) [10]. Thus, we are still in need of effective and safe therapeutic drugs to accompany vaccines, together with molecular constructs, which are tailored precisely to target the key interactions between viral and human proteins [11,12,13]. One such key protein–protein interaction (PPI) involves the receptor-binding domain (RBD) of the viral trimeric glycoprotein spike (S) and the cell surface-exposed human Angiotensin-Converting Enzyme 2 (hACE2) protein, which triggers attachment and subsequent entry of the virus particle into the host cell [5,6,14,15].

Proteins involved in the attachment and entry of the virus have been successfully explored as therapeutic targets for diverse viruses, including HIV-1 [16,17]; indeed, right from the onset of COVID-19, targeting the surface-exposed, extracellular S-RBD:hACE2 interface emerged as one of the first therapeutic approaches to be pursued, because of its potential in halting the SARS-CoV-2 viral infection at a very early stage [5,11]. In addition, this timely recognition event is unique unlike other ensuing interactions, such as the proteolytic cleavage of the spike protein by human proteases, either at the cell surface (viral entry via direct plasma membrane fusion) and/or within endosomes (endocytic pathway) [5,11]. Importantly, the RBD is the primary target of both disease-/vaccine-elicited antibodies and therapeutic monoclonal antibodies, proving the notion that targeting the viral entry can be effective for preventing or stopping virus infectivity [18,19].

**Figure 1:**
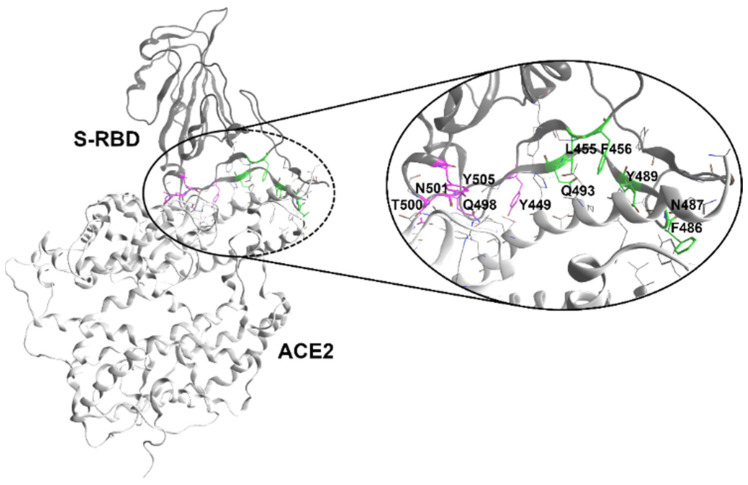
X-ray structure (PDB code 6M0J) of the complex between the human ACE2 (light gray ribbons, **bottom**) and SARS-CoV-2 S-RBD (dark gray ribbons, **top**). The S hot spot residues previously identified by computational alanine scanning [42] are represented in green (binding site BS1) and magenta (binding site BS2).

The S-RBD consists of a core structure and a receptor-binding motif (Figure 1), the latter being responsible for the direct interaction with hACE2. When the S trimer presents its pre-fusion “down” conformation, the receptor-binding motif is nearly inaccessible to the receptor hACE2, while the transition of the RBD from the “down” to the “up” conformation reshapes it to a hACE2 recognizable state, with the “one-RBD-up” being a particularly stable intermediate state that is critical for full engagement with the human receptor [5,14].

In order to target the somewhat extended S-RBD:hACE2 PPI surface, besides the development of large-sized clinically approved neutralizing antibodies (≥140 kDa) [4,20,21], other therapeutically promising protein-based molecules have been identified, including nanobodies (i.e., single domain-antibodies, ca. 70 kDa) [5,22], recombinant human soluble ACE2 (ca. 130 kDa) [13,23], and mini-proteins (56- to 160-mers, ca. 6–36 kDa) [24,25].

On the other hand, several short- or medium-sized peptides, ranging from linear heptapeptides to stapled 30-mers, have been developed, based on the rational design of the binding motifs of either hACE2 or S-RBD [26,27,28,29,30,31,32,33,34,35]. Though the structure of these peptides is much simpler and shorter than that of the protein-based ligands, several showed a binding affinity for S-RBD in the low micromolar or even nanomolar range, as well as a low micromolar inhibitory activity toward pseudo-typed and/or authentic SARS-CoV-2 in vitro. Admittedly, these peptides do not possess the binding affinity and antiviral efficacy of either antibodies, nanobodies, or of mini-proteins; nevertheless, they are proof of the potential that simplified and easy-to-make molecules have in attacking this crucial virus–receptor interaction.

Thus, the use of designed medium-sized peptidomimetics (PMs) [36] in targeting the S-RBD:hACE2 interaction is an appealing strategy, as these PMs should incorporate the qualities of natural peptide-based structures displaying the characteristic weaknesses of both antibodies and peptides/mini-proteins to a lesser degree. In other words, PMs should display traits that are common to natural peptide-based structures such as high specificity and binding affinity toward the biological target and consequently poor off-target effects; reliable and modular preparation; and fairly predictable and tunable ADME behavior. PMs should also, ideally, display the weaknesses of antibodies to a lesser degree, such as costly and batch-dependent quality production, immunogenicity, large size, poor solubility, and difficult adaptation to rapid antigenic viral mutations [20,21,37]. Furthermore, proteolytic degradation and scarce bioavailability, which mar peptides/mini-proteins, should be minimal in PMs [38,39,40,41].

**Figure 2:**
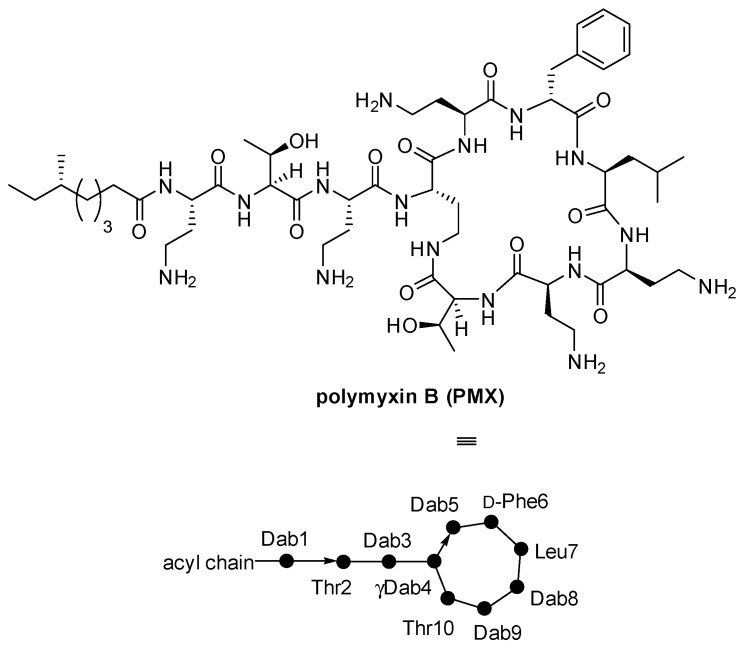
Molecular structure (**top**) and simplified cartoon (**bottom**) of natural polymyxin B (PMX). Filled circles denote amino acid residues; Dab = L-α,γ-diaminobutanoic acid; γDab = cyclized Dab involving the γ-amino function.

In a recent study aimed at in silico drug repurposing for SARS-CoV-2 that employed X-ray-derived structures of the S-RBD:hACE2 interface and computational alanine scanning, we determined the key residues in the S-RBD that majorly contributed to the binding-free energy of the protein interface: two clusters of hot spots were identified, binding site 1 (BS1) and binding site 2 (BS2), which were localized in two distinct and relatively limited regions on the border rim of the targeted interface (Figure 1) [42]. In the same work, a virtual screening (VS) campaign revealed polymyxin B (PMX, Figure 2), a naturally occurring antibiotic, to be a potential binder for both BS1 and BS2 sub-regions.

Based on these precedents, this work aimed to develop novel, medium-sized polymyxin-inspired peptidomimetics that could simultaneously bind to the sub-regions BS1 and BS2 of SARS-CoV-2 S and hinder the interaction with hACE2, thus possibly impeding the entry of SARS-CoV-2 into human cells.

Starting from the in silico design of polymyxin-based peptidomimetics, a collection of six novel heterodimeric PMs, crafted from the covalent conjugation of ten BS1- and BS2-targeted monomers, was identified and synthesized. The binding affinity of these compounds toward the S-RBD of SARS-CoV-2 was determined by surface plasmon resonance (SPR) experiments, and their ability to inhibit infection of authentic SARS-CoV-2 virus in in vitro cellular assays was determined, as described in the next paragraphs.

## 2. Results

As stated in the introduction, the design of our dimeric PM ligands was founded on a previously carried out VS of FDA-approved drugs for the spike RBD [42]. To be specific, starting from the crystal structure of the SARS-CoV-2 S-RBD bound to human ACE2 (PDB code 6M0J), two potential binding regions (BS1 and BS2) were identified by computational alanine scanning (Figure 1). The vVS identified several cyclopeptides as potential binders of either BS1 or BS2, amongst which polymyxin B (PMX, Figure 2) represented the best-ranked hit for both BS1 and BS2 and thus, it was selected for further evaluation. PMX is a naturally occurring amphiphilic lipopeptide produced by fungi (although first isolated by *Bacillus polymyxa*) as a secondary metabolite and it is currently used as a last-resort antibiotic drug for the treatment of Gram-negative multidrug-resistant bacteria, or in topical antibiotic applications [43,44,45]. Its structure features an N-terminating methyloctanoyl linear tripeptide anchored to a cycloheptapeptide ring; the presence of this cyclic restraint together with non-canonical amino acid residues, including six L-α,γ-diaminobutanoic acid residues (Dab) and one D-series residue (D-Phe), qualify it as a natural peptidomimetic [40].

Preliminary SPR assays, conducted on commercially available PMX and using immobilized RBD of the Wuhan-Hu-1 SARS-CoV-2 S protein, detected a minimum of specific binding (Figure S1 in the Supplementary Materials), supporting previous work on molecular modeling [42], and indicating PMX to be an interesting molecular starting platform for the rational design and construction of improved specific cyclic PMs. We hypothesized that the limited performance of natural PMX in recognizing the S-RBD was possibly due to both the high flexibility and lipophilicity of the 6-methyloctanoyl chain, and the highly charged nature of its cycloheptapeptide ring at physiological levels of pH. Moreover, we also hypothesized that a single monomeric ligand, in only partially targeting the S-RBD, might not be sufficient enough to cover the crucial broad spike:hACE2 interface. Based on this assumption, we sought to design more extended ligands capable of covering both BS1 and BS2.

### 2.1. In Silico Design of Polymyxin B-Based Peptidomimetics

**Figure 3:**
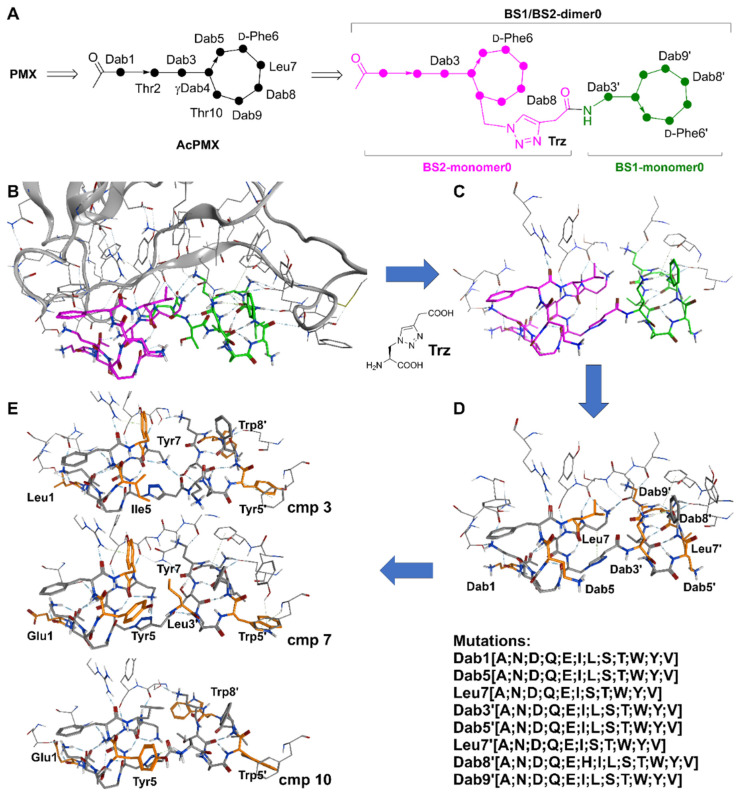
Computational workflow for the design of PMX-based ligands at S-RBD. Unless differently stated, nitrogens, carbons and oxygens are colored in blue, gray and red, respectively. (**A**) Depiction of the chemical structure of the starting dimer0, as derived by ligation of BS1/BS2-monomers0. (**B**) Selected AcPMX poses from docking focused on BS1 (carbons in green) and BS2 (carbons in magenta). (**C**) Preliminary dimer0 structure designed by connecting BS1-monomer0 and BS2-monomer0. BS2-monomer0 obtained by replacing Thr10 of AcPMX with Trz; BS1-monomer0 obtained from AcPMX by deletion of acetyl-Dab1′-Thr2′ residues. (**D**) Planned Residue Scan mutations. Amino acids involved in the RS calculations have orange-colored carbons. (**E**) Dimeric ligands selected after RS calculations followed by MD simulations. Amino acids that have been mutated with respect to dimer0 have orange-colored carbons.

The PMX structure was initially modified in silico by replacing the 6-methyloctanoyl chain with an acetyl (AcPMX) (Figure 3A). AcPMX was then subjected to a conformational search, leading to 19 conformations within a range of about 7 kcal/mol. To maximize the docking sampling ability, each conformation was independently docked to the S-RBD model by focusing on both BS1 and BS2. The results were sorted and the top-ranked poses, within 1 kcal/mol range, were evaluated. One pose for BS1 and one pose for BS2 were selected, with the intent of linking the two monomers into a dimeric structure, without altering the optimal ligand–receptor interactions predicted by docking (Figure 3B).

We envisaged the possibility of replacing Thr10 of AcPMX docked to BS2 with the non-natural amino acid Trz, namely, (*S*)-2-amino-3-[4-(carboxymethyl)-1*H*-1,2,3-triazol-1-yl]propanoic acid (Figure 3C). Trz was designed with a proper length and functionalization to be used as a linker. Moreover, the triazole ring is frequently used as an amide bond bioisostere due to its stability, low flexibility, and synthetic feasibility by click-reaction [46]. Subsequently, the linkage between the two monomers was obtained by an amide bond between the side chain carboxylate of Trz and the α-NH_2_ group of Dab3′ in BS1-bound AcPMX, following the removal of Dab1′ and Thr2′, to obtain the starting dimer0. After a geometry minimization of the complex, all the ligand–receptor interactions predicted by docking remained unaltered. The Residue Scan (RS) tool implemented in the MOE (Molecular Operating Environment) was then used for optimizing the sequence of the dimer0.

RS computes the free energy difference upon the mutation of selected residues with any other natural or non-natural amino acid included in the MOE libraries. Mutations can be single (one residue is mutated at a time) or multiple (up to five residues are mutated at a time). After the mutation, the complex was subjected to short restrained molecular dynamics (MDs) to include the effect of side chain flexibility in the estimation of the change in affinity (ΔAffinity) and in the overall complex stability (ΔStability). Since one of our objectives was to reduce the global positive charge of the ligand, we focused our attention on the Dab residues that were not involved in optimal interactions with the receptor. The apolar Leu7 and Leu7′ residues were also included in the scan to improve the ΔAffinity and ΔStability by maximizing the polar or apolar interactions. The full list of mutations evaluated by RS is given in Figure 3D. Two RS cycles were carried out, the first requesting single point mutations only. The second cycle evaluated multiple mutations, but only of those amino acids that provided improvements in ΔAffinity and ΔStability in the first RS run. More than 200 mutants were obtained, exhibiting negative values for both ΔAffinity and ΔStability. Due to the time constraints, after visual inspection, we selected 10 high-ranked peptides that had ΔAffinity comprised between −16.7 and −12.7 kcal/mol and ΔStability in the range of −3.8 and −0.8 kcal/mol, compared to dimer0 (Table S1 in Supplementary Materials). The corresponding complexes were then subjected to a 200 ns MD simulation to evaluate the dynamic stability of the complex (Figures S2–S7 in Supplementary Materials). Three heterodimers maintained interactions with both the BSs for the whole simulation, namely peptidomimetics **3**, **7**, and **10** (Figure 3E and Supplementary Materials Figures S5–S7), and these were the first set of compounds selected for the chemical synthesis and biochemical evaluation.

### 2.2. Chemical Synthesis of Polymyxin-Based Peptidomimetics

In keeping with the topic of this work, we sought to create new BS1/BS2-targeting peptidomimetic heterodimeric molecules by covalently connecting PMX-based monomers. The designed linker contained quite a short and robust triazolyl-amide moiety, which suggested possible disconnections either along the triazole ring via Huisgen [3+2] cycloaddition of azide- and alkynoyl-ending monomers [47], or along the amide bond between carboxyl- and amine-terminating monomers.

**Figure 4:**
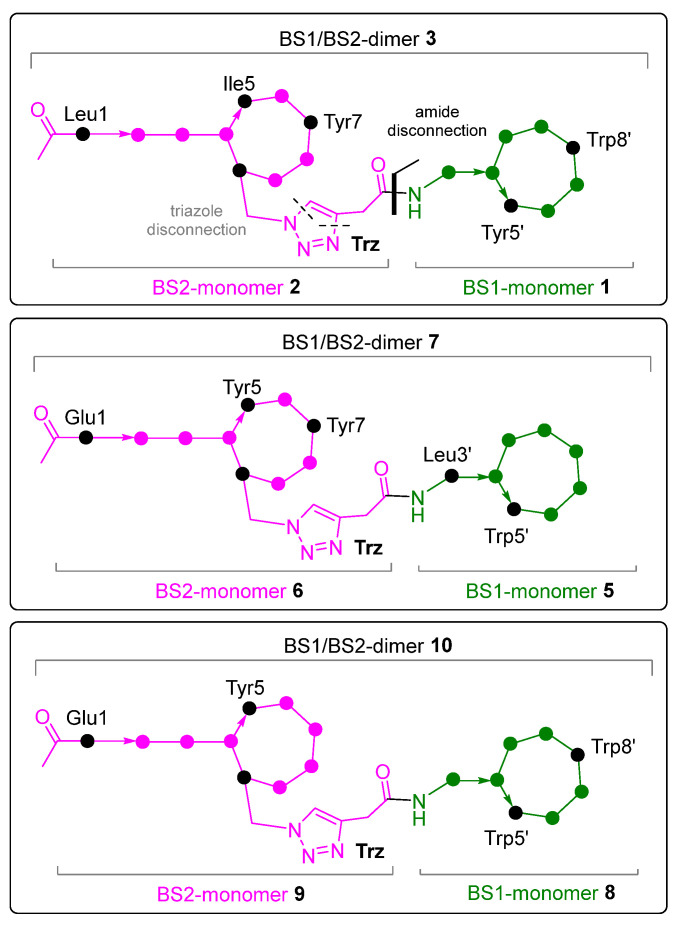
Representation of the structure of the first set of heterodimeric compounds **3**, **7**, and **10** of this work, as derived by amide ligation from the respective monomeric constituents **1**/**2**, **5**/**6**, and **8**/**9**. Filled circles and numbering denote amino acid residues as in the PMX structure in Figure 2. Magenta and green colors used to indicate BS2- and BS1-recognizing monomers, respectively, in line with Figure 3; black circles are amino acid residues modified with respect to the original PMX sequence.

**Scheme 1:**
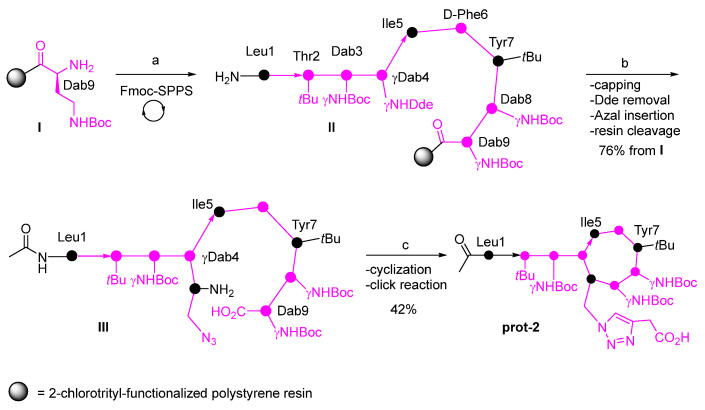
Mixed on-resin and in-solution synthesis of protected BS2-targeted monomers, as exemplified by the synthesis of protected monomer **prot-2**. Magenta color used to indicate BS2-recognizing monomer, in line with Figure 3; black circles are amino acid residues modified with respect to the original PMX sequence. Reagents and conditions: (a): (i) Fmoc-AA-OH, HATU, HOAt, collidine, DMF, 2 h; (ii) 20% piperidine, DMF, 15 min; (b): (i) Ac_2_O, DIPEA, DMF, 30 min; (ii) 4% NH_2_NH_2_/DMF, 2.5 h; (iii) Fmoc-azido-Ala-OH (Fmoc-Azal), HATU, HOAt, collidine, DMF, 2 h; (iv) 20% piperidine, DMF, 15 min; (v) 3:1:1 DCM:AcOH:TFE, 15 min; (c): (i) HATU, HOAt, collidine, 14:1 DCM:DMF (0.7 mM), rt, 5 h; (ii) 3-butynoic acid, Cu(OAc)_2_, sodium ascorbate, 2.4:1 DMF:H_2_O, rt, 6 h.

**Scheme 2:**
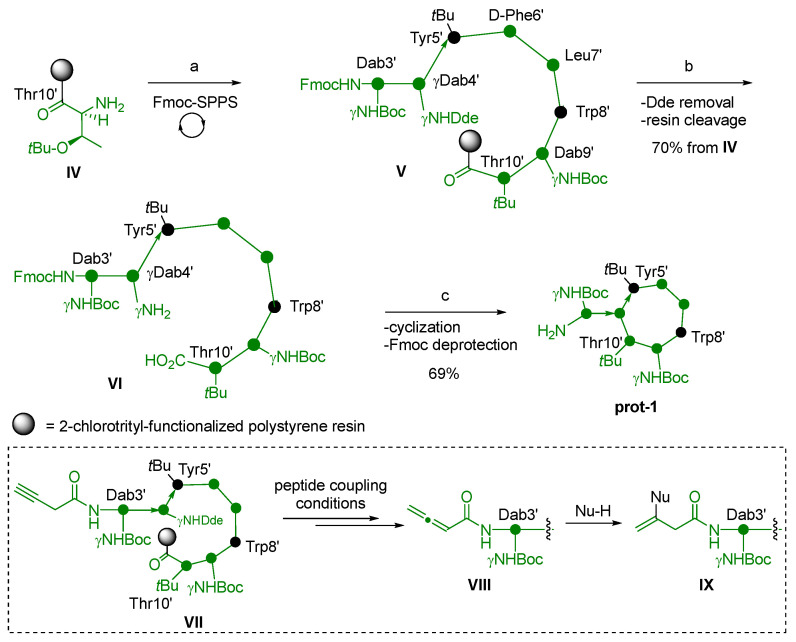
Mixed on-resin and in-solution synthesis of protected BS1-targeted monomers, as exemplified by the synthesis of protected monomer **prot-1**. Green color used to indicate BS1-recognizing monomer, in line with Figure 3; black circles are amino acid residues modified with respect to the original PMX sequence. Dashed box represents possible side reactions of homopropargyl-terminating peptide **VII** under peptide coupling conditions furnishing undesired alkyne-derived allenamide **VIII** and products **IX**. Reagents and conditions: (a) (i) Fmoc-AA-OH, HATU, HOAt, collidine, DMF, 2 h; (ii) 20% piperidine, DMF, 15 min; (b) (i) NH_2_OH(HCl), imidazole, 5:1 NMP:DMF, 3 h; (ii) 3:1:1 DCM:AcOH:TFE, 15 min; (c) (i) HATU, HOAt, collidine, 14:1 DCM:DMF, (0.7 mM), rt, 5 h; (ii) 5% piperidine, ACN, rt, 2 h.

Although the triazole-forming cycloaddition usually proved to be an appealing strategy [47], in the specific case of “short” linkers, such as those of designed target compounds **3**, **7**, and **10** (Figure 4), it was not applicable because the required 3-butinoyl terminus of the BS1-monomer portion would not have survived the standard solid- and/or in-solution peptide synthesis conditions. As consequence, the synthesis was conducted as shown in Scheme 1 and Scheme 2. It is known, in fact, that homopropargyl amides may easily furnish allenamides even under mild coupling conditions, which, in turn, are optimal Michael acceptors with nucleophiles such as thiols and others (see Scheme 2, dashed squared formulas) [48]. For this reason, the disconnection of compound **3** (**7** or **10**) called for the late-stage amide coupling between the appropriate, protected triazolyl carboxylic acid **2** (**6** or **9**) and the protected amine monomer **1** (**5** or **8**), as illustrated in Figure 4.

To access the monomeric constituents, we opted to apply uniformed, mixed solid- and in-solution phase procedures that could be directly applied to obtain all the first-generation and subsequent-generation products required [49]. As shown in Scheme 1 for the prototypic BS2-targeted protected monomer **prot-2**, the synthesis started with commercially available γ-Boc-protected (*S*)-α,γ-diaminobutanoic acid supported on 2-chlorotrityl-functionalized polystyrene resin (compound **I**), constituting the Dab9 residue of the original PMX structure. Standard Fmoc-based SPPS (solid-phase peptide synthesis) followed to afford the fully protected linear peptide **II**, which incorporated six out of seven residues of the cycloheptapeptide core, as well as the tripeptide sequence of the lateral chain. During SPPS, the deprotecting reagent for the Fmoc group was 20% piperidine in DMF, and the coupling conditions entailed the use of HATU and collidine in DMF in the presence of HOAt. Importantly, Dde (*N*-1-(4,4-dimethyl-2,6-dioxocyclohexyliden)ethyl) was used as the orthogonal protecting group of the γ-amine side chain of Dab4 due to its stability in Fmoc-SPPS [49,50].

After capping the free amine terminus within **II** via acetylation, the Dde of Dab4 was selectively removed by 4% NH_2_NH_2_ in DMF. Subsequently, Fmoc-azidoAla-OH (Fmoc-Azal) was coupled under usual solid-phase conditions, followed by Fmoc removal. The linear peptide was released from the resin with the cleavage cocktail DCM:AcOH:TFE (3:1:1), furnishing linear peptide **III** in a 76% yield from **I**. This linear peptide was subjected to in-solution cyclization under HATU/HOAt/collidine in DCM/DMF at 0.7 mM concentration. A cyclic azido-terminating compound was obtained in a 74% yield, which was finally “clicked” with 3-butynoic acid under copper(I)-catalyzed [3+2] cycloaddition conditions. After purification by reverse phase chromatography, the protected monomer **prot-2** was isolated as a colorless glassy solid in a 57% yield.

For BS1-targeted monomer **prot-1**, we again started from the solid-phase construction of the linear peptide sequence, comprising residues of the heptapeptide ring as well as the sole 3′ residue of the lateral chain (Scheme 2). Thus, commercial resin-supported protected threonine **IV**, representing the Thr10′ residue of PMX-like **prot-1**, was the starting residue for Fmoc-based SPPS, which gave linear peptide **V** via the above mentioned Fmoc-deprotection/coupling reagents. However, to preserve the orthogonal Fmoc protection at Dab3′, milder conditions than those adopted for the synthesis of **prot-2** were required to remove the Dde protection at the γ-amino function of Dab4′ (NH_2_OH·HCl, instead of NH_2_NH_2_). The linear peptide **VI** was then detached from resin (overall 70% yield for the SPPS from **IV**) and subjected to in-solution cyclization (87% yield) to consign, after Fmoc-deprotection, the required amine **prot-1** in a 79% yield (corresponding to 48% global yield for the overall sequence from **IV**).

**Scheme 3:**
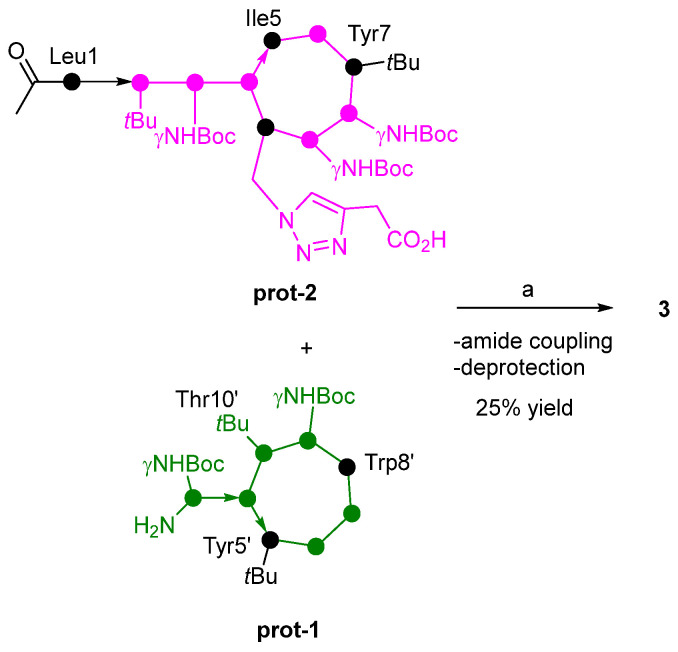
Synthesis of heterodimeric compounds via ligation of protected BS1- and BS2-targeted monomers, as exemplified by the synthesis of heterodimer **3**. Magenta and green colors used to indicate BS2- and BS1-recognizing monomers, respectively, in line with Figure 3; black circles are amino acid residues modified with respect to the original PMX sequence. Reagents and conditions: (a) (i) HATU, HOAt, collidine, DMF, 6 h; (ii) TFA:TIS:H_2_O 95:2.5:2.5, rt, 1 h.

The final amide coupling between carboxylic acid **prot-2** and amine **prot-1** proceeded using the usual HATU/HOAt reagent coupling to furnish, after global acidic deprotection, heterodimer **3** (Scheme 3). After purification by reverse-phase chromatography, heterodimer **3** was obtained in a 25% yield as a glassy solid. Compound **3** exhibited *m*/*z* (M+4H)^4+^ 568.3075, which was consistent with the calculated exact mass *m*/*z* (M+4H)^4+^ 568.3067 and corresponded to 2269.3 Da molecular weight (Table 1). Heterodimers **7** and **10** were obtained in an analogous manner by starting from monomers **prot-6**/**prot-5** and **prot-9**/**prot-8**, respectively (Figure 4, Table 1, and synthetic procedures in the Supplementary Material).

**Figure 5:**
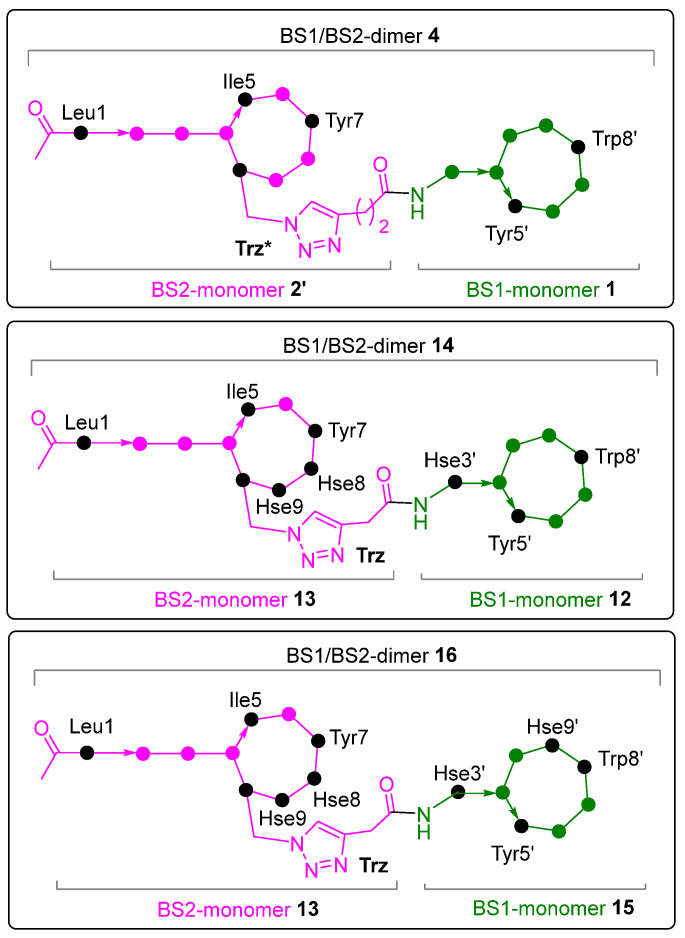
Representation of the structure of the second set of heterodimeric compounds **4**, **14**, and **16** of this work as obtained via amide ligation from the respective monomeric constituents **1**/**2′**, **12**/**13**, and **15**/**13**. Filled circles and numbering denote amino acid residues as in the PMX structure in Figure 2. Magenta and green colors used to indicate BS2- and BS1-recognizing monomers, respectively, in line with Figure 3; black circles are amino acid residues modified with respect to the original PMX sequence. Trz* = (*S*)-2-amino-3-[4-(carboxyethyl)-1*H*-1,2,3-triazol-1-yl]propanoic acid; Hse = homoserine.

To explore even further the chemical space covered by PMs, we next synthesized three additional heterodimers, namely, compounds **4**, **14**, and **16** (Figure 5). Compound **4** differs from dimer **3** just in its spacer length, as it possesses one more methylene unit separating the triazole ring from the carboxyl moiety. From the previous in silico studies (vide supra), it emerged that the two PMX-ring moieties targeting the BS1/BS2 regions were spatially very close, and they tolerated little variation, if any, in the spacer length between them. An evaluation of “elongated” dimer **4** was expected to shed light on this matter.

Another issue of interest concerned the number of basic and, at physiological pH, protonated Dab residues and how this relative proportion influenced the ability of the PMs to recognize the spike protein. For this reason, compounds **14** and **16** featured fewer basic isosteres of Dab, namely, homoserine residues (Hse), instead of certain Dab moieties. In particular, these two compounds share the common BS2-targeted monomer **13** with two Hse residues instead of the original Dab residues (Hse8 and Hse9); however, they differ in the number of Hse moieties in the corresponding BS1-components (one Hse3′ in monomer **12** and two Hse3′/Hse9′ in monomer **15**).

The chemical synthesis of products **4**, **14**, and **16** followed the same path as above, and arose from the ligation of the respective monomers **prot-1**/**prot-2′**, **prot-12**/**prot-13**, and **prot-15**/**prot-13** (Figure 5; for details on the synthesis and characterization of these products, see Table 1 and the Supporting Information). It must be pointed out that Hse-containing heterodimers **14** and **16** exhibited a highly lipophilic character, with consequent issues in solubility and problems encountered in both the HPLC purification step and during the biological assays (vide infra). Nonetheless, our uniformed, modular synthetic strategy proved reliable, and this supported our claim that it can be applied to numerous structural variants.

### 2.3. Binding Affinity of Polymyxin-Based Peptidomimetics to Spike RBD by SPR Analysis

**Figure 6:**
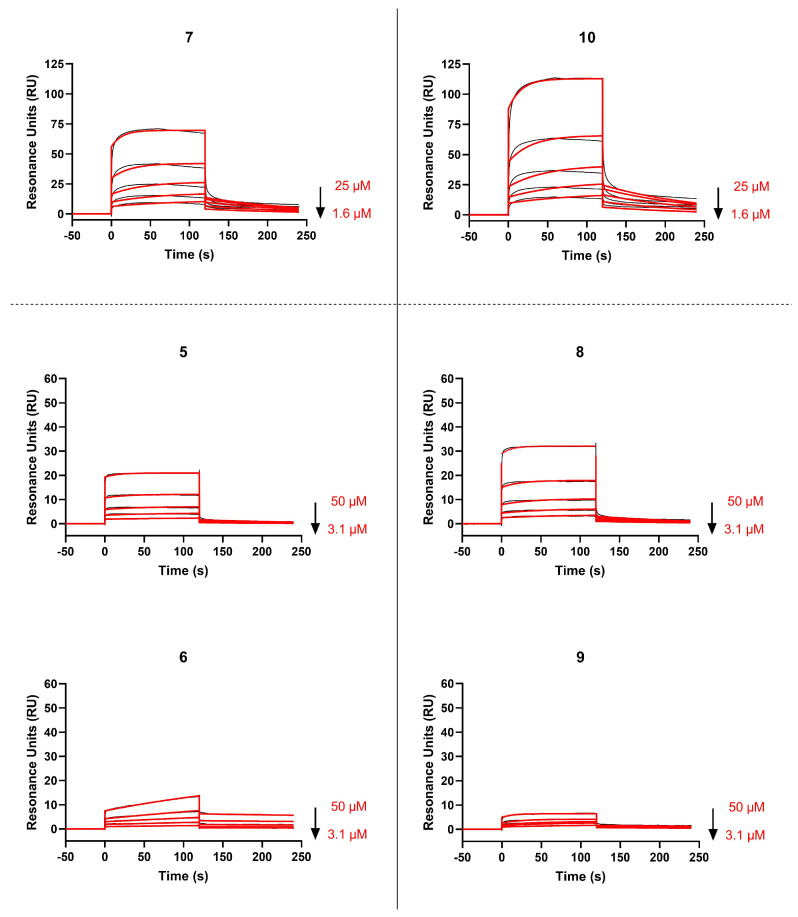
Representative surface plasmon resonance (SPR) sensorgrams showing the binding kinetics for the PMs and immobilized RBD of the Wuhan-Hu-1 spike protein (1:2 dilutions of peptidomimetics, starting from 50 or 25 µM, as indicated). Data are shown as black lines, and the best fit of the data to a 1:1 binding model is shown in red. One representative sensorgram from two independent experiments is shown for each compound. See Table 2 for kinetics values.

To evaluate the biological activity of the synthesized peptidomimetics, we first addressed the binding potential of both heterodimeric and monomeric compounds to the spike protein. More specifically, SPR was used to measure the interaction of compounds (analytes) with immobilized RBD of the Wuhan-Hu-1 SARS-CoV-2 spike protein, as previously reported [51]. This method is useful for measuring the kinetic parameters *k*_a_ (association rate constant) and *k*_d_ (dissociation rate constant), as well as the binding affinity *K*_D_ (dissociation equilibrium constant). The RBD was immobilized on a sensor chip using standard amine coupling. Different concentrations of peptidomimetic compounds (in a 1:2 serial dilution range) were, in turn, passed over the chip surface, and the respective signal changes for each compound were recorded (in Resonance Units (RU)). The resulting data were seen to fit a 1:1 binding model, and the binding affinity and kinetic parameters were determined, as detailed in Figure 6, Table 2, and Supplementary Material Figure S8.

For several compounds, we clearly measured binding to the RBD, albeit with a rather fast release (off rate). In particular, heterodimers **7** and **10** exhibited interesting *K*_D_ values in the low µMolar range (**7**, 2.31 µM; **10**, 2.78 µM), which proved superior, in terms of binding affinity, to those observed for their respective monomers. In fact, monomers **5** and **6**—constituents of **7**—showed a detectable yet returning binding (Figure 6, Table 2), whereas monomers **8** and **9**—constituents of **10**—behaved differently, with **8** displaying an interesting 8.56 µM binding affinity and **9** being devoid of any meaningful RBD binding.

An SPR evaluation of dimers **3** and **4**, on the other hand, did not produce reliable data because of the aspecific interaction with the matrix of the chip, despite the good affinity displayed by their monomeric components (**1**, 10.12 µM; **2**, 9.62 µM) (Figure S8, Supplementary Material).

Moreover, homoserine-containing compounds **12**–**16**, chosen for their lower number of basic Dab residues, did not prove to be good RBD binders either. Among them, dimer **14** exhibited a jagged signal, possibly due to scarce solubility and its tendency to give aggregates in the medium used.

### 2.4. Antiviral Effect of PMX-Inspired Peptidomimetics against SARS-CoV-2

**Figure 7:**
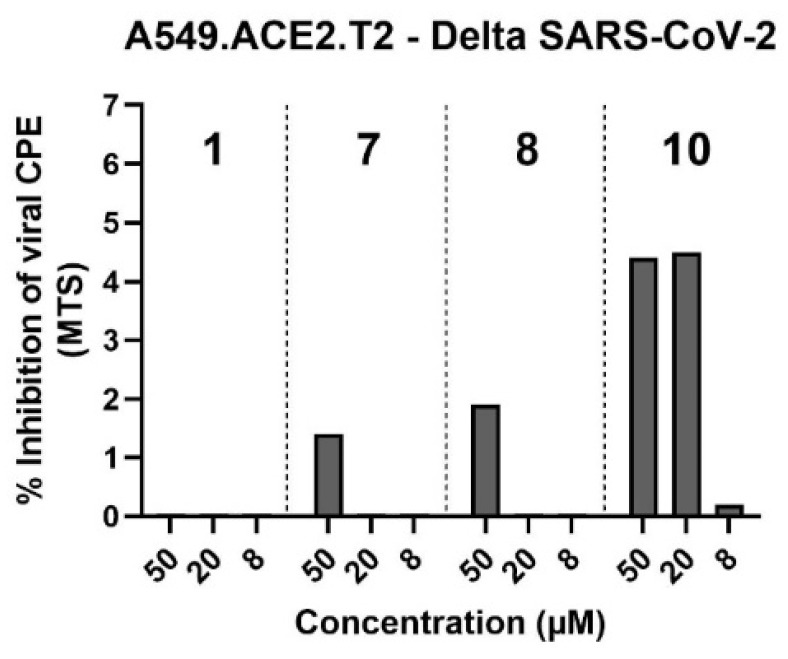
Antiviral effect of peptidomimetics in A549.ACE2.TMPRSS2 cells infected with live Delta SARS-CoV-2. A549.ACE2^+^.TMPRSS2^+^ cells were infected with SARS-CoV-2 (strain Delta) in absence or presence of compounds **1**, **7**, **8**, and **10**, incubated for 5 days, and analyzed by MTS-PES. OD values were used to calculate the % inhibition of viral replication (n = 1).

To further explore the therapeutic potential of the PMs, we next evaluated the dimeric and monomeric compounds in our antiviral assays for SARS-CoV-2. First, the highly permissive glioblastoma cell line U87.ACE2^+^ (that stably expresses the ACE2 receptor) was used for the infection with live SARS-CoV-2 (strain 20A.EU2), as previously reported [52]. As summarized in Table S2 (Supplementary Materials), the compounds did not exert cytotoxic effects up to 50 µM when tested in the U87.ACE2^+^ cells without virus (CC_50_ > 50 µM), nor could they fully protect the cells from being infected with SARS-CoV-2 (IC_50_ > 50 µM). However, dimer **10** had some minor antiviral effects at the highest concentration (50 µM) tested (11% and 2% inhibition of viral cytopathic effect in two independent experiments). Secondly, we investigated the antiviral effect of our compounds in lung epithelial A549 cells stably expressing ACE2 and TMPRSS2, accommodating a plasma membrane entry route of the virus. Similarly, treatment of the A549.ACE2^+^.TMPRSS2^+^ cells with the compounds did not fully prevent the cells from being infected with a Delta strain of SARS-CoV-2 (Table S2 of Supplementary Materials). Interestingly, for compounds **7** and **8**, a hint of antiviral effect was measured at 50 µM concentration, whereas for dimer **10**, some detectable inhibition was recorded at 20 and 50 µM treatment (Figure 7).

## 3. Discussion

The present work was founded on a previous in silico study [42], which suggested that the naturally occurring antibiotic polymyxin B (PMX) was a prime fit for two discrete and non-overlapping regions of the S-RBD:ACE interface, namely, BS1 and BS2. The PMX structure was adopted as an ideal peptidomimetic (PM) starting platform, upon which new PM molecules could be built up for exploring the chemical space around the targeted PPI. Interestingly, PMX and analogues have long been the focus of intense research into the creation of new antibiotics that are effective at overcoming antimicrobial resistance [44,45]; however, to the best of our knowledge, this is the first example where PMX is used as a starting scaffold to construct PMs (resembling yet structurally different from PMX) to combat SARS-CoV-2.

In silico design entailed three levels of intervention to the original PMX structure at the (i) marked shortening of the lipidic side chain into an acetyl moiety to diminish the overall lipophilic character and reduce conformational freedom; (ii) the optimization of the calculated affinity and stability of the complex with the RBD via systematic mutation of non-crucial amino acid residues; and, importantly, (iii) the “heterodimerization” of the PMX structure by connecting two diverse PMX-based cores via a robust linker, with the clear intent of homing in on the two BS1 and BS2 subregions simultaneously [53].

Use of the so-called “discontinued peptides” which span non-contiguous yet spatially close hACE2 fragments, is a good tactic for identifying inhibitors of the SARS-CoV-2 S:hACE2 interface [30,32,54]; this offers small- or medium-sized molecular constructs the opportunity to effectively increase their coverage of the spatially extended targeted interface.

The designed PMs were chemically synthesized via mixed solid-phase and in-solution synthesis, which led to an unprecedented collection of PMX-based heterodimers and monomers with molecular weights ranging from 1 KDa to 2 KDa. Within this collection, two heterodimers, namely, compounds **7** and **10**, exhibited the best binding affinity determined by SPR experiments toward S-RBD, respectively, 2.31 μM and 2.78 μM, which also proved at least three times greater than that of their constituting monomers. This result validated the overall design and molecular modeling studies and corroborated the hypothesis that the simultaneous coverage of the BS1 and BS2 S-RBD subregions by heterodimerization was beneficial for the binding interaction. None of the synthesized peptidomimetics were able to completely inhibit infection of authentic SARS-CoV-2 (strains 20A.EU2 and Delta) in human glioblastoma U87.ACE^+^ cells or in human lung epithelial A549.ACE2.TMPRSS2^+^ cells up to 50 µM concentrations. However, a detailed analysis of the antiviral effect of the compounds revealed some minor protection of the dimers at the highest tested concentration. Especially for dimer **10**, this limited antiviral effect was consistently detectable in both antiviral assays (in which a different combination of virus and cell line was used). This biological effect seems to correlate well with the binding potential determined by our SPR analysis. It seems plausible that, within the quite dynamic and complex cellular environment, the low micromolar binding affinity of our best candidates toward monomeric S-RBD is not enough to warrant efficient competitive neutralization of the trimeric spike in authentic virus particles. Of note, none of the synthesized PMs exerted cytotoxicity up to 50 µM when tested in the non-infected U87 and A549 cell lines (Supplementary Table S2).

In conclusion, heterodimeric compounds **7** and **10** provide a first map of the chemical space around a simplified PMX-resembling core needed to address the S-RBD:hACE2 interaction and they may serve as a useful guide in the search for structurally optimized molecules with an improved S-RBD affinity and anti-SARS-CoV-2 potential. Thus, the rational variation of the constituting amino acid residues, as well as turning the heterodimeric core into a multivalent entity (e.g., trimerization), could be promising options. Finally, such optimized S-RBD binders could serve as directing units within designed covalent conjugates for the targeted delivery of drugs or visualizing tools in the environment of SARS-CoV2-infected cells.

## 4. Materials and Methods

### 4.1. Molecular Modeling

The model of the spike receptor-binding domain (RBD) was prepared as previously described [42]. An initial ligand was designed in MOE [55] based on the structure of polymyxin B where the 6-methyloctanoyl group was replaced by acetyl (AcPMX). AcPMX was subjected to a low-mode conformational search [56] using the Amber10EHT force field and the Born solvation model for water, as implemented in MOE. A total of 19 unique conformations were obtained within an energy range of 6.9 kcal/mol. AcPMX was then docked to the RBD. Two separate runs were carried out, which directed the docking onto each of the two potential binding sites, BS1 and BS2, previously identified by computational alanine scanning [42]. The docking was performed with MOE, using the Triangle Matcher algorithm and the London dG scoring, followed by Rigid Receptor refinement of the top 1000 poses generated in the previous step and rescoring with the GBVI/WSA function [57]. To maximize the sampling, the docking was repeated with each of the previously obtained 19 conformations of AcPMX. The lowest energy poses for both BS1 and BS2 were used to design the dimeric ligand. The two monomers were modified for conjugation within the receptor, keeping all the ligand–receptor interactions generated by docking unaltered. With its optimal side chain length, flexibility and functionalization, and synthetic feasibility, the (*S*)-2-amino-3-[4-(carboxymethyl)-1*H*-1,2,3-triazol-1-yl]propanoic acid (Trz) amino acid was designed to be the linker. Thus, the BS2 monomer was designed from AcPMX by mutating the cyclic Thr to Trz (AcPMX-Trz). The BS1 monomer was obtained by removing the first diaminobutyric acid (Dab) and Thr from the linear peptide chain, thus leaving the α-amino group of the remaining Dab (Dab3) as the anchoring point (Dab-PMX). The starting dimer (dimer0) was then obtained by linking AcPMX-Trz and Dab-PMX through an amide bond between the Trz sidechain carboxylate and the Dab α-NH_2_. The dimer0 sequence was Ac-Dab1-Thr2-Dab3-γDab4‡-Dab5-(D)-Phe6-Leu7-Dab8-Dab9-Trz‡-Dab3′-γDab4′#-Dab5′-(D)-Phe6′-Leu7′-Dab8′-Dab9′-Thr10′#, where the ‡ and # symbols identify residues involved in cyclization. Dimer0 was then processed by multiple sequence–optimization cycles using the Residue Scan (RS) tool, implemented in MOE. The following were initially evaluated as single point mutations (only one mutation is evaluated at a time): Dab1[A;N;D;Q;E;I;L;S;T;W;Y;V], Dab5[A;N;D;Q;E;I;L;S;T;W;Y;V], Leu7[A;N;D;Q;E;I;S;T;W;Y;V], Dab3′[A;N;D;Q;E;I;L;S;T;W;Y;V], Dab5′[A;N;D;Q;E;I;L;S;T;W;Y;V], Leu7′[A;N;D;Q;E;I;S;T;W;Y;V], Dab8′[A;N;D;Q;E;H;I;L;S;T;W;Y;V], Dab9′[A;N;D;Q;E;I;L;S;T;W;Y;V]. Thr2, Dab3, (D)-Phe6, Dab8, Dab9, D-Phe6′ and Thr10′ were excluded in this first round, as they were found to be involved in ligand–receptor interactions, whereas all those mutations that provided a favorable change in both ΔAffinity and ΔStability were reconsidered for a second RS run, where up to five positions were mutated at a time. The following mutations were requested: Dab1[E;D;L;I], Dab5[Y;V;I;L], Leu7Y, Dab3′L, Dab5′[W;Y;L;I], Dab8′W, Dab9′[W;Y;L]. More than 200 mutants were obtained having negative ΔAffinity and ΔStability. The 10 best-ranked mutants for both ΔAffinity and ΔStability were selected for further evaluation by molecular dynamics (MD). Restricted electrostatic potential (RESP) charges were computed for non-natural amino acids (Trz, linear Dab, and cyclic γDab) using the R.E.D. tool [58] by following a well-established protocol [59,60,61]. Each complex was solvated by an octahedral TIP3P water box [62], extending up to 10 Å from the protein, and chlorine ions were added to neutralize the total charge. The ff14SB force field [63] was used for the simulations, and missing parameters for Trz were taken from gaff2 force field [64]. Multiple equilibration steps at constant volume and temperature (NVT) and constant pressure and temperature (NPT) ensembles, were carried out at a final temperature of 300 K, as detailed in previous works [65,66,67]. A production run of 200 ns was then performed, and trajectories were analyzed by computing the root mean squared deviation vs. time. MD simulations and trajectory analyses were performed with the Amber20 and AmberTools21 packages [68]. Three peptides remained stable within the receptor-binding site, namely compounds **3**, **7**, and **10**, which were selected as the first set for synthesis and biochemical evaluation. Variants of these peptides were later designed by following the same strategy as above.

### 4.2. Chemistry

#### 4.2.1. General Procedure A to BS1-Targeted Monomers as Exemplified by the Synthesis of Monomer 1

**Solid-Phase Synthesis.** The synthesis of linear peptide **VI** (Scheme 2) was performed using the preloaded 2-chlorotrityl-Thr(*t*Bu)-H resin **IV** (loading 0.75 mmol/g). *Resin swelling:* the resin (54.6 mg, 0.041 mmol, and 1.0 eq) was swollen in a solid-phase reaction vessel with dry DMF (5 mL) under mechanical stirring; after 40 min the solvent was drained, and the resin was washed with DMF (3×). *Peptide coupling:* A preformed solution of Fmoc-Dab(Boc)-OH (26.9 mg, 0.061 mmol, and 1.5 eq) in dry DMF (2 mL) was treated with HATU (30.8 mg, 0.081 mmol, and 2.0 eq), HOAt (11.0 mg, 0.081 mmol, and 2.0 eq) and 2,4,6-collidine (13.3 µL, 0.081 mmol, and 2.0 eq), and stirred for 5 min before being added to the resin. The mixture was shaken at room temperature for 2 h. Completion of the reaction was checked by the Kaiser test. The solution was drained and the resin was washed with DMF (2×) and DCM (2×). The couplings of Fmoc-Trp(Boc)-OH (32.1 mg, 0.061 mmol, and 1.5 eq), Fmoc-Leu-OH (21.6 mg, 0.061 mmol, and 1.5 eq), Fmoc-D-Phe-OH (23.6 mg, 0.061, and 1.5 eq), Fmoc-Tyr(*t*Bu)-OH (28.0 mg, 0.061 mmol, and 1.5 eq), Fmoc-Dab(Dde)-OH (29.9 mg, 0.061 mmol, and 1.5 eq), and Fmoc-Dab(Boc)-OH (26.9 mg, 0.061 mmol, and 1.5 eq) were carried out under the same conditions. *Fmoc cleavage:* The solution was drained and the resin was washed with DMF (2×) and DCM (2×). The resin was washed again with DMF and treated with 20% *v*/*v* piperidine in DMF (2 mL) and the mixture was stirred for 15 min. The resin was then washed with DMF (2×), DCM (2×), and DMF (1×). *Dde selective cleavage:* The solution was drained and the resin was washed with DMF (2×) and DCM (2×). The resin was washed again with DMF and treated with imidazole (0.92 g for 1 g of resin), Hydroxylamine hydrochloride (1.26 g for 1 g of resin), NMP (5 mL for 1 g of resin), and DMF (1 mL for 1 g of resin) for 3 h. The resin was then washed with DMF (2×), DCM (2×), and DMF (1×). *Resin cleavage:* The resin was treated with 3 mL of the cleavage mixture DCM:TFE:glacial AcOH (3:1:1) and kept under mechanical stirring for 15 min at room temperature. The solution was recovered and the resin was carefully washed with DCM (2×). This protocol was repeated twice. The combined solution was evaporated under reduced pressure affording the linear peptide **VI** (50.0 mg, AcOH salt, 70% yield) as a colorless glassy solid, which was used in the following step without further purification. MS (ES^+^) *m*/*z* 1664.4 [M+H]^+^.

**In-solution cyclization.** To a solution of linear peptide **VI** (50.0 mg, 0.029 mmol, and 1 eq) in dry DCM (8.3 mL), 2,4,6-collidine (14.2 µL, 0.087 mmol, and 3.0 eq) was added. The mixture was stirred under argon at room temperature for 10 min, and then it was added dropwise to a solution of HATU (33.0 mg, 0.087 mmol, amd 3.0 eq) and HOAt (11.8 mg, 0.087 mmol, and 3.0 eq) in dry DMF (2.7 mL) and dry DCM (30 mL). The reaction mixture was degassed by argon/vacuum cycles (3×) and left to stir under argon at room temperature for 5 h. After completion, the solution was concentrated under vacuum. The crude product was purified by reverse phase chromatography (eluent: from 90:10 H_2_O + 0.1% TFA:ACN to 100% ACN) affording the corresponding fully protected cyclopeptide as a colorless glassy solid (41.8 mg, 87% yield). MS (ES^+^) *m*/*z* 1646.5 [M+H]^+^. ^1^H NMR (600 MHz), CD_3_OD: δ 7.71 (m, 2H), 7.54 (m, 3H), 7.34–7.03 (m, 16H), 6.87 (m, 1H), 4.95 (m, 1H), 4.74 (m, 1H), 4.47 (m, 1H), 4.31 (m, 1H), 4.23–4.05 (m, 5H), 3.98 (m, 1H), 3.27–3.00 (m, 7H), 2.89–2.74 (m, 3H), 2.55 (m, 1H), 2.15 (m, 1H), 1.98 (m, 1H), 1.87 (m, 1H), 1.3 (m, 2H), 1.62 (m, 9H), 1.45–1.33 (m, 18H), 1.26 (m, 9H), 1.16 (m, 9H), 0.94 (m, 9H), 0.62 (m, 3H), and 0.51 (m, 3H).

**Fmoc deprotection.** A solution of 5% piperidine in ACN (2.5 mL) was added to the previous fully protected cyclopeptide and the mixture was left to stir at room temperature for 2 h. Subsequently, the solvent was removed under reduced pressure and the crude product was purified by reverse phase chromatography (eluent: from 90:10 H_2_O + 0.1% TFA:ACN to 100% ACN) affording the cyclopeptide **prot-1** as a colorless glassy solid (28.2 mg, TFA salt, and 79% yield). MS (ES^+^) *m*/*z* 1424.2 [M+H]^+^.

**Global deprotection.** Compound **prot-1** (4.8 mg, 0.0029 mmol, and 1 eq) was treated with a solution of TFA/TIS/H_2_O 95:2.5:2.5 (180 μL). After 1 h, the solvent was removed under reduced pressure and the residue was washed with Et_2_O (3×). The resulting crude was then purified by reverse phase chromatography (eluent: from 90:10 H_2_O + 0.1% TFA:ACN to 100% ACN) affording cyclopeptide BS1-monomer **1** as a colorless glassy solid (3.3 mg, 70%). HRMS (ES^+^) *m*/*z* calcd for C_51_H_72_N_12_O_10_^2+^ 506.2669 [M+2H]^2+^, found 506.2762. ^1^H NMR (600 MHz), CD_3_OD: δ 7.56 (d, *J* = 8.1 Hz, 1H), 7.33 (d, *J* = 8.1 Hz, 1H), 7.24–7.07 (m, 7H), 7.04–6.98 (m, 3H), 6.62 (d, *J* = 8.0 Hz, 1H), 4.60 (m, 1H), 4.38 (m, 1H), 4.25 (m, 1H), 4.11–3.99 (m, 3H), 3.44 (m, 1H), 3.19 (m, 1H), 3.12–2.96 (m, 6H), 2.91 (m, 2H), 2.81 (t, *J* = 11.3 Hz, 1H), 2.27–2.10 (m, 4H), 2.05–1.93 (m, 2H), 1.36–1.24 (m, 6H), 1.20 (d, *J* = 5.3 Hz, 3H), 0.98 (m, 1H), 0.87 (m, 1H), 0.56 (d, *J* = 6.6 Hz, 3H), and 0.53 (d, *J* = 6.4 Hz, 3H).

#### 4.2.2. General Procedure B to BS2-Targeted Monomers as Exemplified by the Synthesis of Monomer 2

**Solid-Phase Synthesis.** The synthesis of linear peptide **III** (Scheme 1) was performed following the procedure described for **VI**, starting from the preloaded 2-chlorotrityl-Fmoc-Dab(Boc)-H resin (215.0 mg, resin loading: 0.277 mmol/g, and 1 eq). Peptide couplings were performed using Fmoc-Dab(Boc)-OH (39.2 mg, 0.089 mmol, and 1.5 eq), Fmoc-Tyr(*t*Bu)-OH (40.9 mg, 0.089 mmol, and 1.5 eq), Fmoc-D-Phe-OH (34.5 mg, 0.089 mmol, and 1.5 eq), Fmoc-Ile-OH (31.4 mg, 0.089, and 1.5 eq), Fmoc-Dab(Dde)-OH (43.7 mg, 0.089 mmol, and 1.5 eq), Fmoc-Dab(Boc)-OH (39.2 mg, 0.089 mmol, and 1.5 eq), Fmoc-Thr(*t*Bu)-OH (35.4, 0.089 mmol, and 1.5 eq), Fmoc-Leu-OH (31.5 mg, 0.089 mmol, and 1.5 eq), and Fmoc-β-azido-Ala-OH (31.3 mg, 0.089 mmol, and 1.5 eq). *Capping procedure:* The capping reaction was performed using 50 eq of acetic anhydride, 50 eq of DIPEA in DMF (1–2 mL). The mixture was left to stir for 30 min and completion of reaction was checked by Kaiser test. *Dde cleavage:* Cleavage of the Dde protecting group was performed using a 4% solution of monohydrated hydrazine in DMF (1–2 mL). The reaction was left to stir for 2.5 h. The linear peptide was cleaved from the resin as described for compound **VI**, affording the linear peptide **III** (75.5 mg, AcOH salt, 76% yield) as a colorless glassy solid, which was used as such in the subsequent step without further purification. MS (ES^+^) *m*/*z* 1624.3 [M+H]^+^.

**In-solution cyclization.** The cyclization was performed as described for compound **prot-1**, starting from linear peptide **III** (75.5 mg, 0.049 mmol, and 1 eq). After completion, the solution was concentrated under vacuum. The crude product was purified by reverse phase chromatography (eluent: from 90:10 H_2_O + 0.1% TFA:ACN to 100% ACN) affording the corresponding cyclopeptide as a colorless glassy solid (53.6 mg, 74% yield). MS (ES^+^) *m*/*z* 1605.2 [M+H]^+^. ^1^H NMR (600 MHz), CD_3_OD: δ 7.25–7.03 (m, 7H), 6.87 (m, 2H, ArH), 4.65–4.27 (m, 8H), 4.11 (m, 1H), 4.01 (m, 1H), 3.84–3.67 (m, 2H), 3.25–2.83 (m, 14H), 2.09–1.79 (m, 8H), 1.71–1.56 (m, 4H), 1.41 (m, 27H), 1.28 (m, 9H), 1.18 (m, 9H), 1.09 (m, 3H), 0.93 (m, 3H), 0.89 (m, 3H), 0.75 (m, 3H), and 0.58 (m, 3H).

**Click reaction.** To a solution of the previous compound (20.5 mg, 0.013 mmol, and 1 eq) and 3-butynoic acid (3.2 mg, 0.038 mmol, and 3 eq) in DMF (2.4 mL), a solution of Cu(OAc)_2_ (0.75 mg, 0.004 mmol, and 0.3 eq) and sodium ascorbate (1.5 mg, 0.008 mmol, and 0.6 eq) in water (1 mL) was added. The reaction was left under stirring under argon atmosphere after 3 cycles of argon/vacuum. After 6 h, the solvent was removed under reduced pressure and the residue was washed with water (3×) and diethyl ether (3×). The crude product was purified by reverse phase chromatography (eluent: from 90:10 H_2_O + 0.1% TFA:ACN to 100% ACN) affording the cyclopeptide **prot-2** as a colorless glassy solid (12.3 mg, 57% yield). MS (ES^+^) *m*/*z* 1690.2 [M+H]^+^. ^1^H NMR (400 MHz), CD_3_OD: δ 7.90 (bs, 1H), 7.28–7.06 (m, 7H), 6.87 (m, 2H), 4.95 (m, 2H), 4.71 (m, 1H), 4.58 (m, 1H), 4.48–4.27 (m, 5H), 4.17 (m, 1H), 3.97 (m, 1H), 3.77 (m, 2H), 3.28–2.87 (m, 12H), 2.09–1.79 (m, 10H), 1.73–1.56 (m, 4H), 1.44 (m, 27H), 1.32 (m, 9H), 1.22 (m, 9H), 1.13 (m, 3H), 1.01–0.93 (m, 6H), 0.79 (m, 3H), and 0.60 (m, 3H).

**Global deprotection.** Compound **prot-2** (5.7 mg, 0.0034 mmol, and 1 eq) was treated with a solution of TFA/TIS/H_2_O 95:2.5:2.5 (190 μL). After 1 h, the solvent was removed under reduced pressure and the residue was washed with Et_2_O (3×). The resulting crude was then purified by reverse phase chromatography (eluent: from 90:10 H_2_O + 0.1% TFA:ACN to 100% ACN) affording the protected cyclopeptide BS2-monomer **2** as a colorless glassy solid (2.5 mg, 45%). HRMS (ES^+^) *m*/*z* calcd for C_59_H_72_N_17_O_15_^3+^ 426.2241 [M+3H]^3+^, found 426.2333. ^1^H NMR (600 MHz), CD_3_OD: δ 7.89 (bs, 1H), 7.26–7.13 (m, 5H), 7.00 (m, 2H), 6.68 (m, 2H), 4.93 (m, 1H), 4.45–4.17 (m, 8H), 3.74 (m, 2H), 3.13–2.90 (m, 11H), 2.39–2.14 (m, 3H), 2.14–1.87 (m, 8H), 1.72–1.57 (m, 5H), 1.39–1.24 (m, 6H), 1.18 (m, 3H), 0.94 (m, 3H), 0.89 (m, 3H), 0.73 (m, 3H), and 0.62 (m, 3H).

#### 4.2.3. General Procedure C to BS1/BS2-Targeted Heterodimers as Exemplified by the Synthesis of Dimer 3 (Scheme 3)

To a solution of compound **prot-2** (6.4 mg, 0.004 mmol, and 1 eq), HATU (2.9 mg, 0.008 mmol, and 2 eq), HOAt (1.0 mg, 0.008 mmol, and 1 eq), and 2,4,6-collidine (1.8 μL, 0.011 mmol, and 3 eq) in DMF (0.4 mL), a solution of **prot-1** (7.4 mg, 0.005 mmol, and 1.2 eq) in DMF (0.1 mL) was added. The reaction was left to stir at room temperature under nitrogen atmosphere for 6 h. Subsequently, the solvent was removed under reduced pressure and the crude was washed with water (5×). The resulting crude was then treated with a solution of TFA/TIS/H_2_O (95:2.5:2.5) (209 μL). After 1 h, the solvent was removed under reduced pressure and the solid residue was washed with Et_2_O (3×). The resulting crude was purified by reverse phase chromatography (eluent: from 90:10 H_2_O + 0.1% TFA:ACN to 100% ACN) affording the cyclopeptide BS1/BS2-dimer **3** as a colorless glassy solid (2.8 mg, 25% yield for 2 steps). HRMS (ES^+^) *m*/*z* calcd for C_110_H_161_N_29_O_24_^4+^ 568.3067 [M+4H]^4+^ found 568.3075.

### 4.3. Biology

#### 4.3.1. Surface Plasmon Resonance

SPR technology (Biacore T200, Cytiva, Marlborough, MA, USA) was used to determine the binding kinetics and affinity of the peptidomimetics to the RBD of the wild-type Wuhan-Hu-1 SARS-CoV-2 spike protein (2019-nCoV spike RBD, cat n° 40592-VNAH, SinoBiological, Beijing, China). The RBD was immobilized on a CM5 sensor chip using standard amine coupling in 10 mM HEPES (pH 7.0) at a level between 5000 and 7000 RU. Interaction studies between peptidomimetics and the RBD were performed at 25 °C in HBS-P^+^ (10 mM HEPES, 150 mM NaCl, and 0.05% surfactant P20; pH 7.4). Two-fold serial dilutions of peptidomimetics were injected at 30 µL/min using multiple cycle kinetics. Totals of 10 mM NaOH and 10 mM Glycine-HCl (pH 1.7) were used to regenerate the RBD and antibody surface, respectively. Several buffer blanks were included for double referencing. Apparent binding kinetics (*K*_D_, *k*_a_, and *k*_d_) were derived after fitting the experimental data to the 1:1 Langmuir binding model using the Biacore T200 Evaluation Software 3.1. The experiments were performed at least in duplicate.

**Viruses.** All virus-related work was conducted in the high-containment biosafety level 3 facilities of the Rega Institute at the Katholieke Universiteit (KU) Leuven (Leuven, Belgium), in accordance with institutional guidelines. Severe acute respiratory syndrome coronavirus 2 (SARS-CoV-2) isolates were recovered from nasopharyngeal swabs of RT-qPCR-confirmed human cases obtained from the University Hospital (Leuven, Belgium). SARS-CoV-2 viral stocks were prepared by inoculation of confluent Vero E6 cells or A549.ACE2^+^.TMPRSS2^+^, as described in detail [69] Titers were determined by tissue culture infectious dose 50 (TCID_50_) method of Reed and Muench on A549.ACE2^+^.TMPRSS2^+^ and U87.ACE2^+^ cells. Viral genome sequence was verified, and all infections were performed with passage 2 to 5 virus.

#### 4.3.2. Wild-Type Virus Infection and Antiviral Assays

One day prior to the experiment, A549.ACE2^+^.TMPRSS2^+^ (Invivogen, San Diego, CA, USA) or U87.ACE2^+^ cells [52] were seeded in 96-well microtiter plates. The following day, 2.5- or 5-fold serial dilutions of the test compounds were prepared in virus infection media (similar to cell culture medium, but with reduced FBS content), overlaid on cells, and virus was added to each well. Cells were incubated at 37 °C under 5% CO_2_ for the duration of the experiment. At endpoints (4 or 5 days p.i.), the virus-induced cytopathic effect (CPE) was microscopically evaluated. Inhibition was calculated by comparison to virus control wells with no inhibitor added. IC_50_ values were determined by interpolation. Four (U87.ACE2^+^ cells) or five (A549.ACE2^+^.TMPRSS2^+^cells) days after infection, the cell viability of mock- and virus-infected cells was assessed spectrophotometrically via the in situ reduction of 3-(4,5-dimethylthiazol-2-yl)-5-(3-carboxy-methoxyphenyl)-2-(4-sulfophenyl)-2*H*-tetrazolium inner salt, using the CellTiter 96 Aqueous One Solution Cell Proliferation Assay (Promega, Madison, WI, USA). The optical density (OD) of the samples was compared to appropriate cell control replicates (cells without virus and drugs) and virus control wells (cells with virus but without drugs). The concentration that inhibited SARS-CoV-2-induced cell death by 50% (IC_50_) was calculated from interpolation.

## Figures and Tables

**Table 1:** Amino acid sequence of PMX and heterodimeric compounds in this work ^(a)^.

Compd	Sequence	*m*/*z ^(^*^b)^ Found (calcd)	MW (Da)
**PMX**	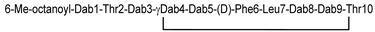	nd	1203.5
**3**	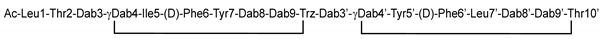	568.3075 (568.3067) [M+4H]^4+^	2269.3
**4**	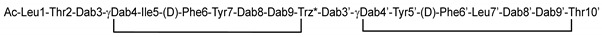	571.5606 (571.5607) [M+4H]^4+^	2283.7
**7**	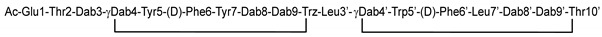	572.2990 (572.2964) [M+4H]^4+^	2285.6
**10**	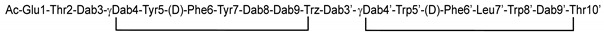	578.0528 (578.0504) [M+4H]^4+^	2308.6
**14**	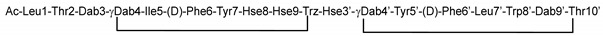	758.0576 (758.0570) [M+3H]^3+^	2272.6
**16**	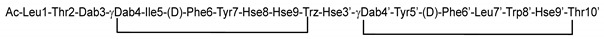	1137.0737 (1137.0737) [M+2H]^2+^	2273.6

^(a)^ Abbreviations: Trz = (*S*)-2-amino-3-[4-(carboxymethyl)-1*H*-1,2,3-triazol-1-yl]propanoic acid; Trz* = (*S*)-2-amino-3-[4-(carboxyethyl)-1*H*-1,2,3-triazol-1-yl]propanoic acid; Dab = L-α,γ-diaminobutanoic acid; γDab = cyclized Dab involving the γ-amino function; Hse = homoserine. ^(b)^ HRMS (ES^+^).

**Table 2:** Surface plasmon resonance (SPR) analysis of PMX-based peptidomimetics ^(a)^.

Ligand/Analyte	*k*_a_ (×10^3^ M^−1^ s^−1^)	*k*_d_ (×10^−3^ s^−1^)	*K*_D_ (µM)
RBD/dimer **7**	3.09 ± 0.59	6.83 ± 1.58	2.31 ± 0.95
RBD/monomer **5**	n.a.	n.a.	n.a.
RBD/monomer **6**	n.a.	n.a.	n.a.
RBD/dimer **10**	2.65 ± 0.67	7.00 ± 1.10	2.78 ± 1.12
RBD/monomer **8**	1.20 ± 0.41	9.43 ± 1.52	8.56 ± 4.16
RBD/monomer **9**	n.a.	n.a.	n.a.
RBD/dimer **3**	n.a.	n.a.	n.a.
RBD/dimer **4**	n.a.	n.a.	n.a.
RBD/monomer **1**	0.61 ± 0.05	6.12 ± 0.79	10.12 ± 2.09
RBD/monomer **2**	0.46 ± 0.02	4.39 ± 0.39	9.62 ± 1.25

^(a)^ Kinetic parameters from different repeat SPR experiments performed in this study. Given are the association rate constant (*k*_a_), the dissociation rate constant (*k*_d_), and the dissociation equilibrium constant (*K*_D_). Values are mean ± SD, n = 2. n.a.: not applicable due to non-quantifiable kinetic parameters. Ligand: immobilized RBD of the Wuhan-Hu-1 SARS-CoV-2 spike protein; analyte: peptidomimetics of this work.

## Data Availability

The data are contained within the manuscript and the Supplementary Material.

## Supplementary Materials

The supporting information can be downloaded at: https://www.mdpi.com/article/10.3390/ijms24108765/s1.
