# Supplementary material for: Novel Polymyxin-Inspired Peptidomimetics Targeting the SARS-CoV-2 Spike:hACE2 Interface
Source: Int J Mol Sci. 2023 May 15;24(10):8765. doi: 10.3390/ijms24108765 (PMC10218303; doi:10.3390/ijms24108765)
Supplement: Supplementary file 1 [file ijms-24-08765-s001.zip › ijms-2360025-supplementary.pdf]

Supplementary Material

Novel Polymyxin-Inspired Peptidomimetics Targeting the SARS-CoV-2 Spike:hACE2 Interface

Kelly Bugatti, Andrea Sartori, Lucia Battistini, Crescenzo Coppa, Emiel Vanhulle, Sam Noppen, Becky Provinciael, Lieve Naesens, Annelies Stevaert, Alessandro Contini *, Kurt Vermeire † and Franca Zanardi *,†

*Correspondence: alessandro.contini@unimi.it (A.C.); franca.zanardi@unipr.it (F.Z.)
† These authors contributed equally to this work.

Summary

1. SPR analysis of Polymyxin B (Figure S1) S2
2. Molecular modelling (Table S1, Figures S2-S7) S3
3. Chemistry S8
3.1. General methods and materials S8
3.2. Synthetic procedures S9
4. HPLC chromatograms S22
5. NMR spectra S27
6. High resolution mass spectra S42
7. SPR analysis of PMX-Based Peptidomimetics (Figure S8) S57
8. Antiviral properties of the synthesized peptidomimetics (Table S2) S58

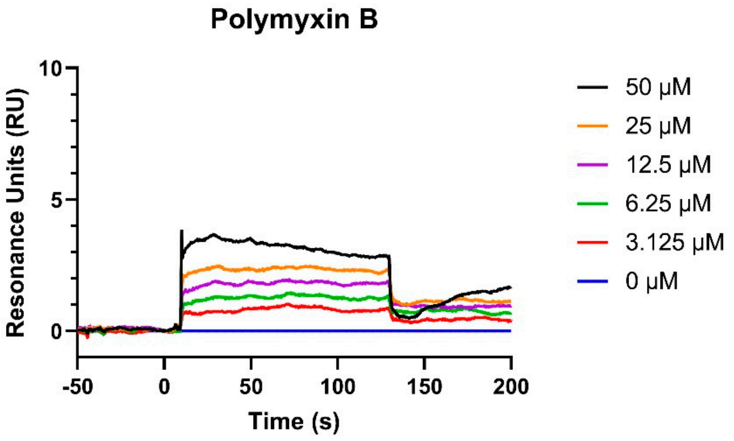

Figure S1. Surface plasmon resonance (SPR) analysis of Polymyxin B. Representative SPR sensorgram showing the binding kinetics for PMX and immobilized RBD of Wuhan-Hu-1 spike protein (1:2 dilutions of PMX, starting from 50 μM). RBD was coupled to a Cytiva CM4 chip at 527 RU. One representative sensorgram out of 2 is shown.

2. Molecular modelling

Table S1. Top 50 Mutants Obtained by Multiple "Residue Scan" Cycles with MOE Software.

Mutant	Mutation	ΔAffinity[a]	ΔStability[a]
1	2:(Dab)1[(Dab)] 2:(Dab)5V 2:L7Y 2:(Dab)13I 2:(Dab)17W	-16.7	-1.9
2	2:(Dab)1I 2:(Dab)5Y 2:(Dab)11[(Dab)] 2:(Dab)13W 2:(Dab)17W	-16.5	-3.6
3	2:(Dab)1D 2:(Dab)5I 2:(Dab)11[(Dab)] 2:(Dab)13Y 2:(Dab)17W	-15.0	-3.1
**4 (Cmp 3)**	**2:(Dab)1L 2:(Dab)5I 2:L7Y 2:(Dab)13Y 2:(Dab)16W**	**-14.7**	**-2.4**
**5 (Cmp 7)**	**2:(Dab)1E 2:(Dab)5Y 2:L7Y 2:(Dab)11L 2:(Dab)13W**	**-14.7**	**-3.6**
6	2:(Dab)1E 2:(Dab)5I 2:L7Y 2:(Dab)11[(Dab)] 2:(Dab)13W	-14.5	-1.0
7	2:(Dab)1E 2:(Dab)5L 2:L7Y 2:(Dab)13W 2:(Dab)17Y	-14.5	-2.8
8	2:(Dab)1E 2:(Dab)5V 2:L7Y 2:(Dab)13Y 2:(Dab)17[(Dab)]	-14.1	-0.8
9	2:(Dab)1D 2:(Dab)5L 2:L7Y 2:(Dab)11L 2:(Dab)13W	-13.8	-3.3
10	2:(Dab)5[(Dab)] 2:L7Y 2:(Dab)11[(Dab)] 2:(Dab)13I 2:(Dab)17W	-13.8	-2.2
**11 (Cmp 10)**	**2:(Dab)1E 2:(Dab)5Y 2:L7L 2:(Dab)13W 2:(Dab)16W**	**-13.5**	**-2.1**
12	2:(Dab)1D 2:(Dab)5L 2:L7Y 2:(Dab)11[(Dab)] 2:(Dab)13Y	-13.3	-1.2
13	2:(Dab)5[(Dab)] 2:L7L 2:(Dab)11[(Dab)] 2:(Dab)13Y 2:(Dab)17W	-13.3	-0.8
14	2:(Dab)1I 2:(Dab)5Y 2:L7Y 2:(Dab)11L 2:(Dab)13Y	-13.2	-3.8
15	2:(Dab)1E 2:(Dab)5Y 2:L7Y 2:(Dab)13W 2:(Dab)17Y	-13.0	-1.9
16	2:(Dab)1L 2:(Dab)5[(Dab)] 2:L7Y 2:(Dab)11L 2:(Dab)13I	-12.8	-3.1
17	2:(Dab)1D 2:L7Y 2:(Dab)11L 2:(Dab)13L 2:(Dab)17Y	-12.7	-1.8
18	2:(Dab)1D 2:L7Y 2:(Dab)11L 2:(Dab)13Y 2:(Dab)17W	-12.7	-1.8
19	2:(Dab)1D 2:(Dab)5V 2:L7Y 2:(Dab)13Y 2:(Dab)17W	-12.7	-1.5
20	2:(Dab)1L 2:L7Y 2:(Dab)11[(Dab)] 2:(Dab)13W 2:(Dab)17W	-12.7	-0.5
21	2:(Dab)1D 2:(Dab)5I 2:L7Y 2:(Dab)11L 2:(Dab)17W	-12.6	-3.1
22	2:(Dab)1D 2:L7Y 2:(Dab)11[(Dab)] 2:(Dab)13L 2:(Dab)17W	-12.6	-1.3
23	2:(Dab)1D 2:(Dab)5Y 2:L7Y 2:(Dab)11L 2:(Dab)17W	-12.5	-3.8
24	2:(Dab)1D 2:(Dab)5V 2:L7Y 2:(Dab)11L 2:(Dab)17W	-12.5	-3.4
25	2:(Dab)1E 2:(Dab)5Y 2:L7Y 2:(Dab)11L 2:(Dab)17W	-12.5	-3.7
26	2:(Dab)1D 2:(Dab)5I 2:L7Y 2:(Dab)11[(Dab)] 2:(Dab)13W	-12.5	-0.8
27	2:(Dab)1E 2:L7Y 2:(Dab)11[(Dab)] 2:(Dab)13L 2:(Dab)17W	-12.4	-1.5
28	2:(Dab)1L 2:(Dab)5[(Dab)] 2:L7Y 2:(Dab)11L 2:(Dab)13W	-12.3	-2.3
29	2:(Dab)1L 2:(Dab)5[(Dab)] 2:L7Y 2:(Dab)13W 2:(Dab)17Y	-12.3	-1.0
30	2:(Dab)1E 2:(Dab)5V 2:L7Y 2:(Dab)11L 2:(Dab)17W	-12.1	-3.5
31	2:(Dab)1L 2:(Dab)5L 2:L7Y 2:(Dab)11L 2:(Dab)13Y	-12.1	-3.4
32	2:(Dab)1D 2:L7Y 2:(Dab)11L 2:(Dab)13L 2:(Dab)17W	-12.0	-3.1
33	2:(Dab)1[(Dab)] 2:L7Y 2:(Dab)11L 2:(Dab)13Y 2:(Dab)17Y	-12.0	-2.3
34	2:(Dab)1L 2:(Dab)5Y 2:L7Y 2:(Dab)11L 2:(Dab)17W	-11.9	-4.0
35	2:(Dab)1E 2:L7L 2:(Dab)11L 2:(Dab)13[(Dab)] 2:(Dab)17Y	-11.9	-2.0
36	2:(Dab)1E 2:(Dab)5L 2:(Dab)11[(Dab)] 2:(Dab)13W 2:(Dab)17W	-11.9	-1.7
37	2:(Dab)1E 2:(Dab)5L 2:L7Y 2:(Dab)13W 2:(Dab)17[(Dab)]	-11.9	-0.6
38	2:(Dab)1D 2:(Dab)5L 2:(Dab)11[(Dab)] 2:(Dab)13W 2:(Dab)17W	-11.9	-1.5
39	2:(Dab)5I 2:L7Y 2:(Dab)11[(Dab)] 2:(Dab)13W 2:(Dab)16W	-11.9	-1.0
40	2:(Dab)1E 2:(Dab)5I 2:(Dab)11[(Dab)] 2:(Dab)13W 2:(Dab)17W	-11.9	-2.1
41	2:(Dab)1E 2:(Dab)5I 2:L7Y 2:(Dab)11L 2:(Dab)17W	-11.8	-3.2
42	2:(Dab)1E 2:(Dab)5Y 2:L7Y 2:(Dab)11L 2:(Dab)13L	-11.8	-3.5
43	2:(Dab)1E 2:(Dab)5Y 2:(Dab)11[(Dab)] 2:(Dab)13W 2:(Dab)17W	-11.8	-1.0

44	2:(Dab)1E 2:(Dab)5L 2:L7L 2:(Dab)13W 2:(Dab)17Y	-11.8	-2.2
45	2:(Dab)1I 2:(Dab)5L 2:L7Y 2:(Dab)13[(Dab)] 2:(Dab)17W	-11.8	-1.8
46	2:(Dab)1I 2:(Dab)5[(Dab)] 2:L7Y 2:(Dab)11L 2:(Dab)13W	-11.8	-2.3
47	2:(Dab)1E 2:L7L 2:(Dab)11[(Dab)] 2:(Dab)13W 2:(Dab)17W	-11.8	-1.5
48	2:(Dab)1E 2:(Dab)5L 2:L7Y 2:(Dab)13I 2:(Dab)17W	-11.8	-1.6
49	2:(Dab)1D 2:(Dab)5Y 2:L7Y 2:(Dab)13W 2:(Dab)17[(Dab)]	-11.8	-1.1
50	2:(Dab)1D 2:(Dab)5V 2:(Dab)11[(Dab)] 2:(Dab)13W 2:(Dab)17W	-11.8	-1.9

[a] ΔAffinity and ΔStability (kcal/mol) are relative energies referred to the dimer0 peptide.

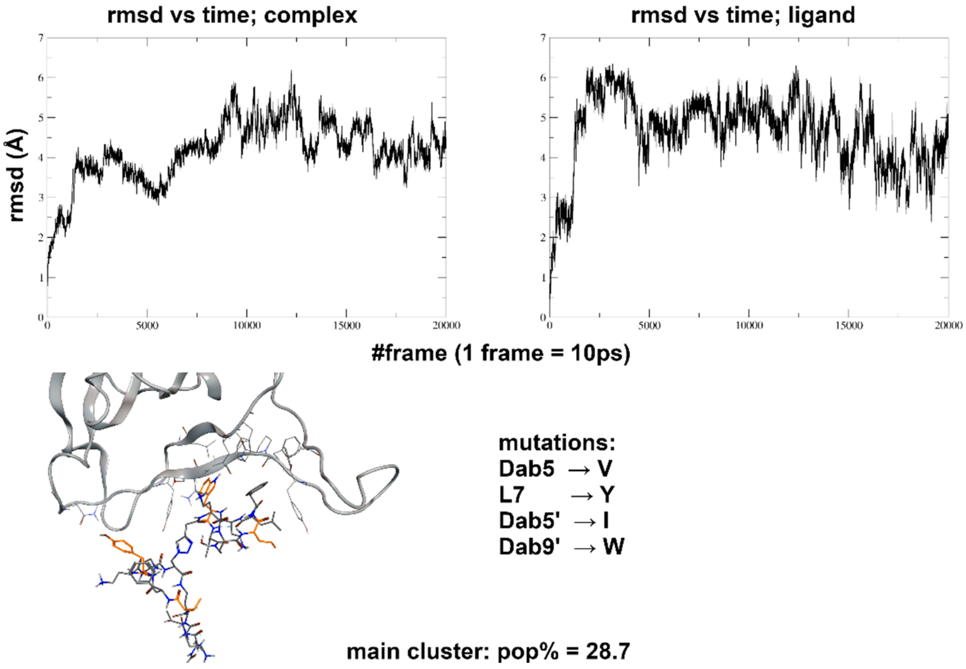

Figure S2. Top: backbone rmsd *vs* time profiles for the 200 ns MD simulation of Mutant 1 (Table S1). Bottom: Structure of the representative geometry of the most populated cluster derived by cluster analysis of the 100-200 ns segment of the MD trajectory.

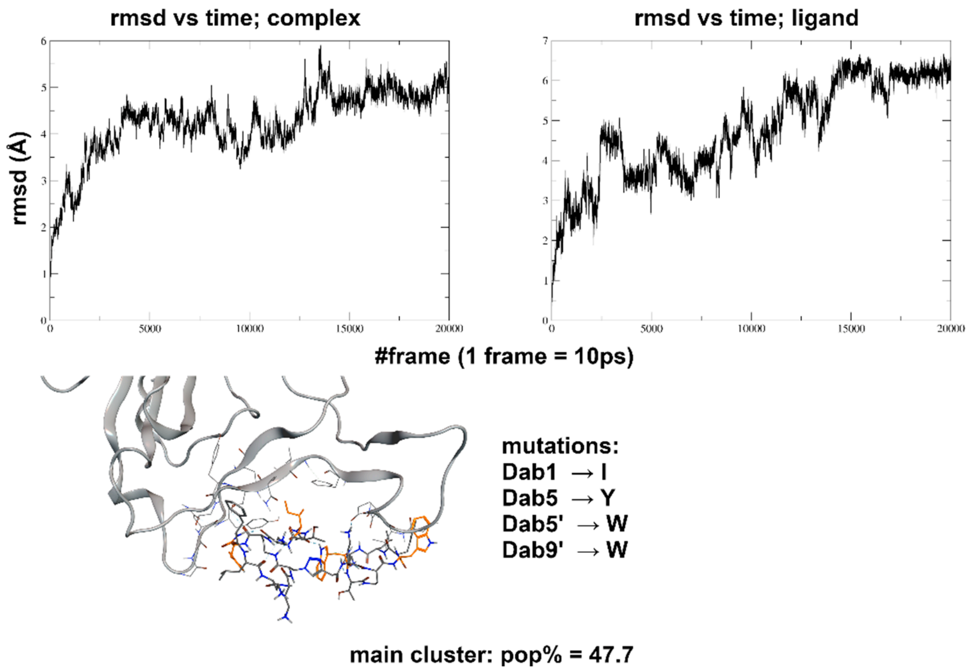

Figure S3. Top: backbone rmsd *vs* time profiles for the 200 ns MD simulation of Mutant 2 (Table S1). Bottom: Structure of the representative geometry of the most populated cluster derived by cluster analysis of the 100-200 ns segment of the MD trajectory.

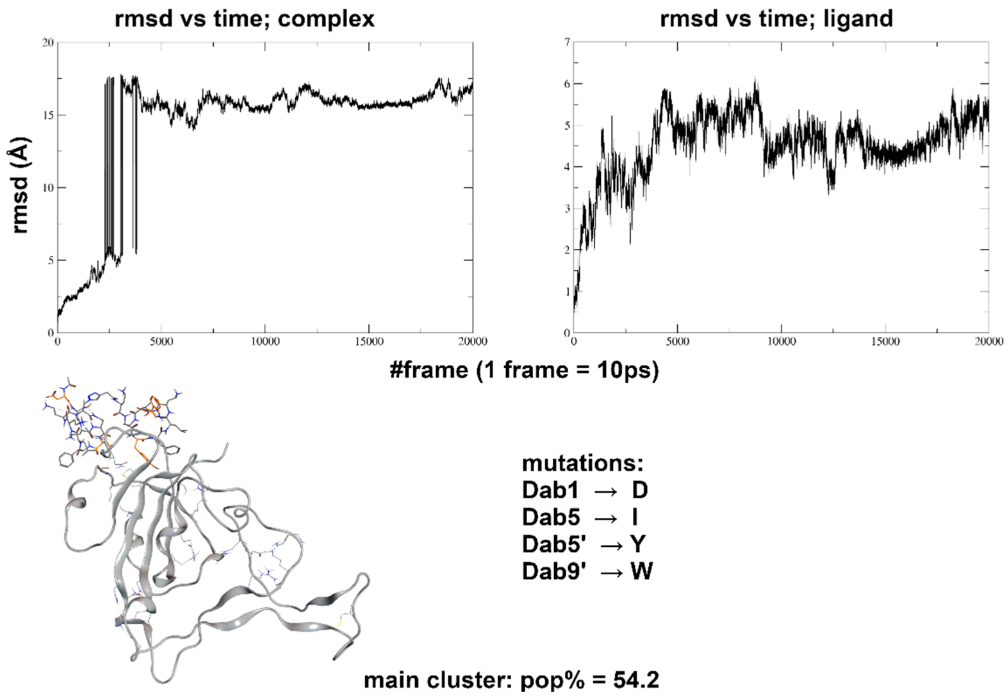

Figure S4. Top: backbone rmsd *vs* time profiles for the 200 ns MD simulation of Mutant 3 (Table S1). Bottom: Structure of the representative geometry of the most populated cluster derived by cluster analysis of the 100-200 ns segment of the MD trajectory.

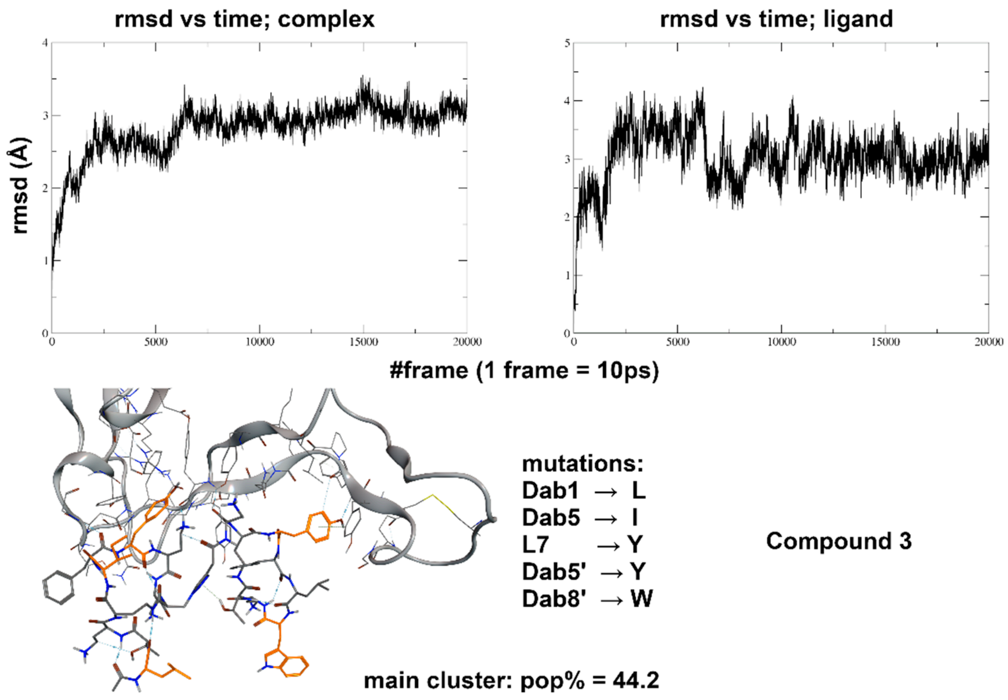

Figure S5. Top: backbone rmsd *vs* time profiles for the 200 ns MD simulation of Mutant 4 (alias compound **3**, Table S1). Bottom: Structure of the representative geometry of the most populated cluster derived by cluster analysis of the 100-200 ns segment of the MD trajectory.

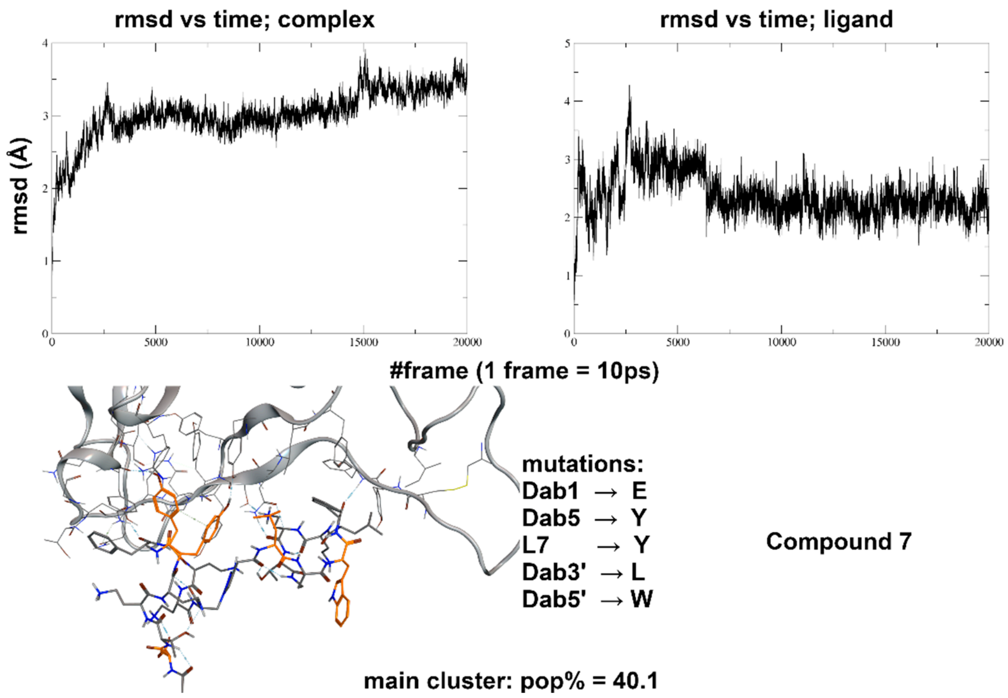

Figure S6. Top: backbone rmsd vs time profiles for the 200 ns MD simulation of Mutant 5 (alias Compound **7**, Table S1). Bottom: Structure of the representative geometry of the most populated cluster derived by cluster analysis of the 100-200 ns segment of the MD trajectory.

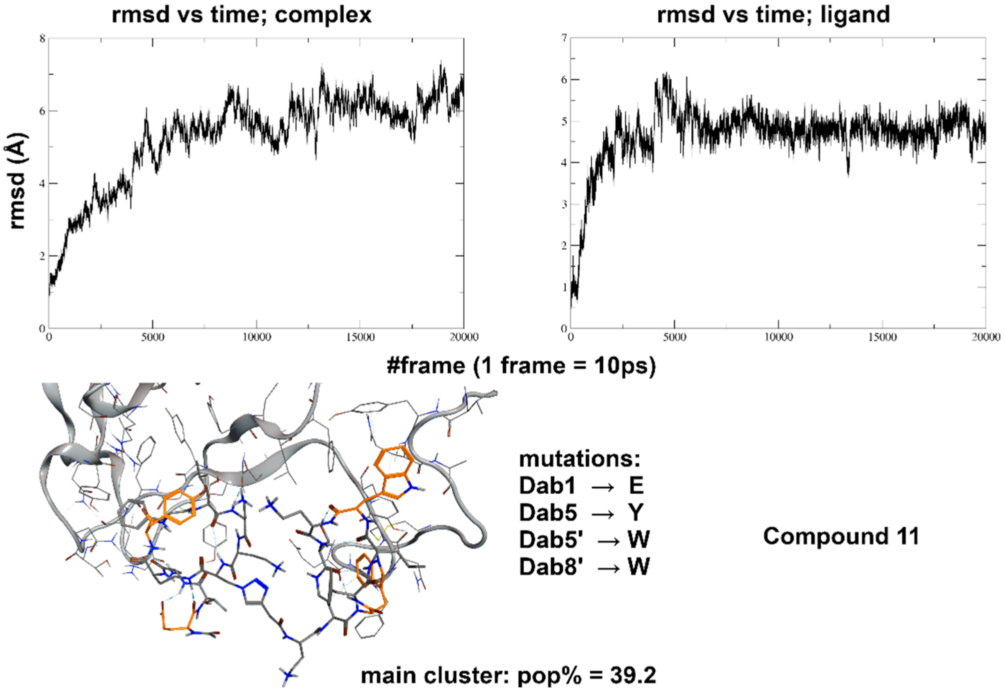

Figure S7. Top: backbone rmsd vs time profiles for the 200 ns MD simulation of Mutant 11 (alias Compound **10**, Table S1). Bottom: Structure of the representative geometry of the most populated cluster derived by cluster analysis of the 100-200 ns segment of the MD trajectory.

3. Chemistry

3.1. General methods and materials

General. All chemicals were of the highest commercially available quality and were used without further purification. Solvents were dried by standard procedures and reactions requiring anhydrous conditions were performed under nitrogen or argon atmosphere. All the reagents were purchased from Merck-Sigma-Aldrich and Fluorochem. The automated flash chromatography and HPLC solvents respond to ACS standard, and they were used without further purification. Analytical thin layer chromatography (TLC) was performed on silica gel 60 F_{254} pre-coated plates with visualization under short-wavelength UV light and by dipping the plates with molybdate reagent (aqueous H_2SO_4 solution of ceric sulphate/ammonium molybdate) followed by heating. Flash column chromatography was performed using 40-63 µm silica gel and the indicated solvent mixtures. Automated flash column chromatography was carried out with the Biotage Isolera One system using KP-C18-HS cartridges (reverse phase). ESI-mass spectra were recorded on UHPLC/ESI-MS system (ACQUITY Ultra Performance LC; ESI, positive ions, Single Quadrupole analyzer) and are reported in the form of (*m/z*). Routine NMR spectra were recorded on Avance 400 (Bruker) or 600 (Jeol) NMR spectrometers. Chemical shifts (δ) are reported in parts per million (ppm) with TMS ($CDCl_3$), CD_2HOD, and HOD resonance peaks set at 0, 3.31, and 4.80 ppm, respectively. Multiplicities are indicated as s (singlet), d (doublet), t (triplet), q (quartet), m (multiplet), and b (broad). Coupling constants, *J*, are reported in Hertz. ^{1}H NMR assignments were corroborated by 1D and 2D experiments (^{1}H-^{1}H COSY). High resolution mass analysis (ESI) was performed on LTQ ORBITRAP XL Thermo apparatus. The high-resolution mass analysis of dimers **3**, **7** and **10** were calculated considering one ^{13}C carbon isotope, since it corresponded to the most intense signal.

Abbreviations. ACN, acetonitrile; Ac_2O, acetic anhydride; AcOH, acetic acid; Boc, *tert*-butoxycarbonyl; 2,4,6-collidine, 2,4,6-trimethylpiridine; $Cu(OAc)_2$, copper(II) acetate; DCE, 1,2-dichloroethane; DCM, dichloromethane; Dde [*N*-1-(4,4-dimethyl-2,6-dioxocyclohexyliden)ethyl]; DIPEA, diisopropylethylamine; DMF, *N,N*-dimethylformamide; EtOAc, ethyl acetate; HATU, 1-[bis(dimethylamino)methylene]-1*H*-1,2,3-triazolo[4,5-b]pyridinium 3-oxide hexafluorophosphate, otherwise known as hexafluorophosphate *O*-(7-azabenzotriazol-1-yl)-*N,N,N',N'*-tetramethyl uronium; HOAt, 1-hydroxy-7-azabenzotriazole; NMP, N-methyl-2-pyrrolidone; TFE, 2,2,2-trifluoroethanol; TIS, triisopropylsilane.

Materials. H-Thr(*t*Bu)-2-ClTrt resin, 2-Cl-Trt chloride resin, Fmoc-Dab(Boc)-OH, Fmoc-Dab(Dde)-OH, Fmoc-Leu-OH, Fmoc-Phe-OH, Fmoc-D-Phe-OH, Fmoc-Tyr(*t*Bu)-OH, Fmoc-D-Tyr(*t*Bu)-OH, Fmoc-Ile-OH, Fmoc-Trp(Boc)-OH, Fmoc-Thr(*t*Bu)-OH, Fmoc-Glu(*t*Bu)-OH, Fmoc-Hse(Trt)-OH, Fmoc-β-azido-Ala-OH, 2,4,6-collidine, glacial acetic acid, DIPEA, HATU, HOAt, DMP, NMP, imidazole, monohydrated hydrazine, monohydrated hydroxylamine, trifluoroethanol, 3-butynoic acid, 4-pentynoic acid, triisopropylsilane were commercially available and were used as such without further purification.

General procedure for Kaiser test. A few drops of *solution A* (80% phenol solution in ethanol), *solution B* (6% ninhydrin solution in ethanol) and *solution C* (98:2 – pyridine/KCN aq. 0.1 mM) were added to a small sample of the resin [pre-washed with MeOH (2x)], and then heated to 100 °C for 2 min. If resin beads maintained their yellow colour, quantitative coupling was achieved. In case of blue resin beads, the coupling step was not fully completed, and it was then repeated.

General procedure for loading resin. 2-chlorotrityl resin (1 eq) was swelled for 30 min in DCM. Then, the resin was washed and a solution of Fmoc-Aaa-OH (0.5 eq) and DIPEA (1.5 eq) in DCM (1-2 mL) was added. The mixture was left to stir for 12 h; then the resin was washed with DCM (2x) and a solution of DCM/MeOH/DIPEA (85:10:5) (3x10 min). The loading of the final resin was measured by photometric analysis (see above).

General procedure for calculating the resin loading. The prepared resin (10 mg) was carefully weighed and put in an Eppendorf tube. Then, 800 µL of DMF were added and the resin was allowed swelling for 15 min. Then, piperidine (200 µL) was added, and the resulting solution was efficiently mixed and left standing for 15 min at room

temperature. Subsequently, this solution (100 µL) was transferred in 1 cm quartz cuvette and DMF (900 µL) was added. The absorbance (A_{301}) was detected at 301 nm versus a DMF blank. The final loading (L) was calculated using the following formula:

L = (A_{301} x V x d)/(Ec x w x M)

L = resin loading (mmol/gram)
A_{301} = absorbance at 301 nm
V = Volume of cleavage solution (1 mL)
d = dilution (10)
E_c = Extinction coefficient = 7800 mL/mmol*cm
W = Width of the cuvette (1 cm)
M = weight of the resin sample

General HPLC method. The purity of all the final compounds was tested using Shimadzu System (LC-30), equipped with an analytical C-18 column (Supelco, DiscoveryBio Wide Pore c18-10, 25 cm x 4.6 mm, 10 µm), using the following method:

Time (min)	H_2O+0.1% TFA	CH_3OH	Flow (mL/min)
0	90%	10%	1
22	60%	40%	1
25	60%	40%	1
30	90%	10%	1

3.2. Synthetic procedures

General Procedure A to BS1-targeted monomers as exemplified by the synthesis of monomer 1.

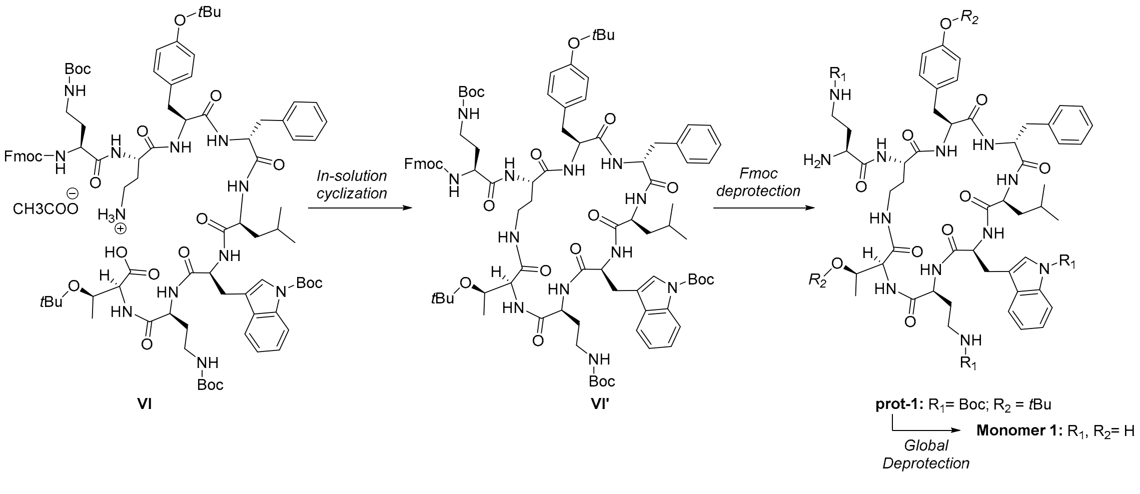

Solid Phase Synthesis. The synthesis of linear peptide **VI** (Scheme 2, main text) was performed using the preloaded 2-chlorotrityl-Thr(*t*Bu)-H resin **IV** (loading 0.75 mmol/g). *Resin swelling*: the resin (54.6 mg, 0.041 mmol, 1.0 eq) was swollen in a solid phase reaction vessel with dry DMF (5 mL) under mechanical stirring; after 40 min the solvent was drained, and the resin was washed with DMF (3×). *Peptide coupling:* A preformed solution of Fmoc-Dab(Boc)-OH (26.9 mg, 0.061 mmol, 1.5 eq) in dry DMF (2 mL) was treated with HATU (30.8 mg, 0.081 mmol, 2.0 eq), HOAt (11.0 mg, 0.081 mmol, 2.0 eq) and 2,4,6-collidine (13.3 µL, 0.081 mmol, 2.0 eq) and stirred for 5 min before being adding to the resin. The mixture was shaken at room temperature for 2 h. Completion of the reaction was checked by the Kaiser test. The solution was drained and the resin was washed with DMF (2×) and DCM (2×). The couplings of Fmoc-Trp(Boc)-OH (32.1 mg, 0.061 mmol, 1.5 eq), Fmoc-Leu-OH (21.6 mg, 0.061 mmol, 1.5 eq), Fmoc-D-Phe-OH (23.6 mg,

0.061, 1.5 eq), Fmoc-Tyr(*t*Bu)-OH (28.0 mg, 0.061 mmol, 1.5 eq), Fmoc-Dab(Dde)-OH (29.9 mg, 0.061 mmol, 1.5 eq), Fmoc-Dab(Boc)-OH (26.9 mg, 0.061 mmol, 1.5 eq) were carried out under the same conditions. *Fmoc cleavage:* The solution was drained and the resin was washed with DMF (2×) and DCM (2×). The resin was washed again with DMF and treated with 20% v/v piperidine in DMF (2 mL) and the mixture was stirred for 15 min. The resin was then washed with DMF (2x), DCM (2x) and DMF (x1). *Dde selective cleavage:* The solution was drained and the resin was washed with DMF (2×) and DCM (2×). The resin was washed again with DMF and treated with imidazole (0.92 g for 1g of resin), Hydroxylamine hydrochloride (1.26 g for 1g of resin), NMP (5 mL for 1 g of resin) and DMF (1 mL for 1g of resin) for 3h. The resin was then washed with DMF (2x), DCM (2x) and DMF (x1). *Resin cleavage*: the resin was treated with 3 mL of the cleavage mixture DCM/TFE/glacial AcOH (3:1:1) and kept under mechanical stirring for 15 min at room temperature. The solution was recovered and the resin was carefully washed with DCM (2×). This protocol was repeated twice. The combined solution was evaporated under reduced pressure affording the linear peptide **VI** (50.0 mg, AcOH salt, 70% yield) as a colourless glassy solid, which was used in the following step without further purification. MS (ES$^+$) *m/z* 1664.4 $[M+H]^+$.

In-solution cyclization. To a solution of linear peptide **VI** (50.0 mg, 0.029 mmol, 1 eq) in dry DCM (8.3 mL), 2,4,6-collidine (14.2 µL, 0.087 mmol, 3.0 eq) was added. The mixture was stirred under argon at room temperature for 10 min, then it was added dropwise to a solution of HATU (33.0 mg, 0.087 mmol, 3.0 eq) and HOAt (11.8 mg, 0.087 mmol, 3.0 eq) in dry DMF (2.7 mL) and dry DCM (30 mL). The reaction mixture was degassed by argon/vacuum cycles (3×) and left to stir under argon at room temperature for 5 h. After completion, the solution was concentrated under vacuum. The crude product was purified by reverse phase chromatography (eluent: from 90:10 H_2O+0.1%TFA:ACN to 100% ACN) affording the corresponding fully protected cyclopeptide **VI'** as a colourless glassy solid (41.8 mg, 87 % yield). MS (ES$^+$) *m/z* 1646.5 $[M+H]^+$. ^{1}H NMR (600 MHz), CD_3OD: δ 7.71 (m, 2H), 7.54 (m, 3H), 7.34-7.03 (m, 16H), 6.87 (m, 1H), 4.95 (m, 1H), 4.74 (m, 1H), 4.47 (m, 1H), 4.31 (m, 1H), 4.23-4.05 (m, 5H), 3.98 (m, 1H), 3.27-3.00 (m, 7H), 2.89-2.74 (m, 3H), 2.55 (m, 1H), 2.15 (m, 1H), 1.98 (m, 1H), 1.87 (m, 1H), 1.3 (m, 2H), 1.62 (m, 9H), 1.45-1.33 (m, 18H), 1.26 (m, 9H), 1.16 (m, 9H), 0.94 (m, 9H), 0.62 (m, 3H), 0.51 (m, 3H).

Fmoc deprotection. A solution of 5% piperidine in ACN (2.5 mL) was added to the previous fully protected cyclopeptide and the mixture was left to stir at room temperature for 2h. Subsequently, the solvent was removed under reduced pressure and the crude product was purified by reverse phase chromatography (eluent: from 90:10 H_2O+0.1%TFA:ACN to 100% ACN) affording the cyclopeptide **prot-1** as a colourless glassy solid (28.2 mg, TFA salt, 79% yield). MS (ES$^+$) *m/z* 1424.2 $[M+H]^+$.

Global deprotection. Compound **prot-1** (4.8 mg, 0.0029 mmol, 1 eq) was treated with a solution of TFA/TIS/H_2O 95:2.5:2.5 (180 µL). After 1 h, the solvent was removed under reduced pressure and the residue was washed with Et_2O (3x). The resulted crude was then purified by reverse phase chromatography (eluent: from 90:10 H_2O+0.1%TFA:ACN to 100% ACN) affording cyclopeptide **BS1-monomer 1** as a colourless glassy solid (3.3 mg, 70%). HRMS (ES$^+$) *m/z* calcd for $C_{51}H_{72}N_{12}O_{10}^{2+}$ 506.2669 $[M+2H]^{2+}$, found 506.2762. ^{1}H NMR (600 MHz), CD_3OD: δ 7.56 (d, *J* = 8.1 Hz, 1H), 7.33 (d, *J* = 8.1 Hz, 1H), 7.24-7.07 (m, 7H), 7.04-6.98 (m, 3H), 6.62 (d, *J* = 8.0 Hz, 1H), 4.60 (m, 1H), 4.38 (m, 1H), 4.25 (m, 1H), 4.11-3.99 (m, 3H), 3.44 (m, 1H), 3.19 (m, 1H), 3.12-2.96 (m, 6H), 2.91 (m, 2H), 2.81 (t, *J* = 11.3 Hz, 1H), 2.27-2.10 (m, 4H), 2.05-1.93 (m, 2H), 1.36-1.24 (m, 6H), 1.20 (d, *J* = 5.3 Hz, 3H), 0.98 (m, 1H), 0.87 (m, 1H), 0.56 (d, *J* = 6.6 Hz, 3H), 0.53 (d, *J* = 6.4 Hz, 3H).

Synthesis of monomer 5

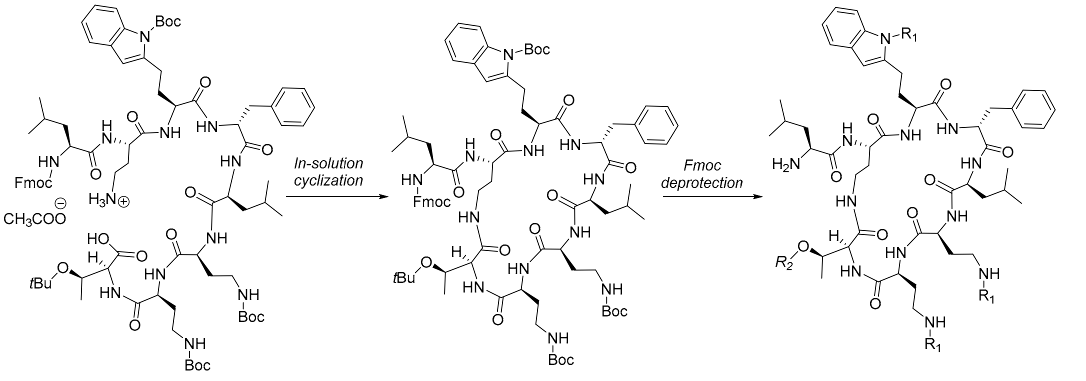

X **X'**

prot-5: R_1= Boc; R_2 = *t*Bu → **Monomer 5:** R_1, R_2= H (Global Deprotection)

Solid Phase Synthesis. The synthesis of linear peptide **X** (see the above scheme) was performed following the general procedure A. Peptide couplings were performed using Fmoc-Dab(Boc)-OH (36.9 mg, 0.084 mmol, 1.5 eq), Fmoc-Dab(Boc)-OH (36.9 mg, 0.084 mmol, 1.5 eq), Fmoc-Leu-OH (29.7 mg, 0.084 mmol, 1.5 eq), Fmoc-D-Phe-OH (32.5 mg, 0.084, 1.5 eq), Fmoc-Trp(Boc)-OH (44.2 mg, 0.084 mmol, 1.5 eq), Fmoc-Dab(Dde)-OH (41.2 mg, 0.084 mmol, 1.5 eq), Fmoc-Leu-OH (29.4, 0.084 mmol, 1.5 eq). The resin was cleaved affording the linear peptide **X** (78.9 mg, AcOH salt, 81% yield) as a colourless glassy solid, which was used as such in the following step without further purification. MS (ES^+) *m/z* 1558.9 $[M+H]^+$.

In-solution cyclization. The cyclization was performed following the general procedure A, starting from linear peptide **X** (78.9 mg, 0.049 mmol, 1 eq). After the purification, the protected cyclopeptide **X'** was collected as a colourless glassy solid (58.6 mg, 78% yield). MS (ES^+) *m/z* 1539.8 $[M+H]^+$.

Fmoc deprotection: Compound **X'** was deprotected following the general procedure A. After purification, the cyclopeptide **prot-5** was obtained as a colourless glassy solid (43.8 mg, TFA salt, 80% yield). MS (ES^+) *m/z* 1318.8 $[M+H]^+$.

Global deprotection: Compound **prot-5** (10.6 mg, 0.0074 mmol, 1 eq) was deprotected as described for **prot-1**. After purification, cyclopeptide **BS1-monomer 5** was obtained as a colourless glassy solid (6.1 mg, 63%). HRMS (ES^+) *m/z* calcd for $C_{48}H_{74}N_{12}O_9^{2+}$ 481.2773 $[M+2H]^{2+}$, found 481.2874. ^1H NMR (600 MHz), CD_3OD: δ 7.56 (d, *J* = 8.3 MHz, 1H), 7.32 (d, *J* = 8. MHz, 1H), 7.24-6.99 (m, 7H), 4.58 (m, 1H), 4.47 (m, 1H), 4.42 (t, *J* = 7.3 MHz, 1H), 4.26 (t, *J* = 7.9 MHz, 1H), 4.17 (m, 1H), 4.07 (m, 3H), 3.85 (m, 1H), 3.56 (m, 1H), 3.23 (m, 1H), 3.12-2.99 (m, 7H), 2.74 (m, 1H), 2.66 (bt, *J* = 11.7 MHz, 1H), 2.53 (m,1H), 2.28 (m, 1H), 2.15 (m, 2H), 2.05-1.93 (m, 2H), 1.77-1.62 (m, 2H), 1.46 (m, 1H), 1.27 (m, 3H), 1.19 (d, *J* = 6.7 MHz, 3H), 0.98 (d, *J* = 6.2 MHz, 3H), 0.87 (d, *J* = 5.9 MHz, 3H), 0.62 (m, 3H), 0.54 (m, 3H).

Synthesis of monomer 8

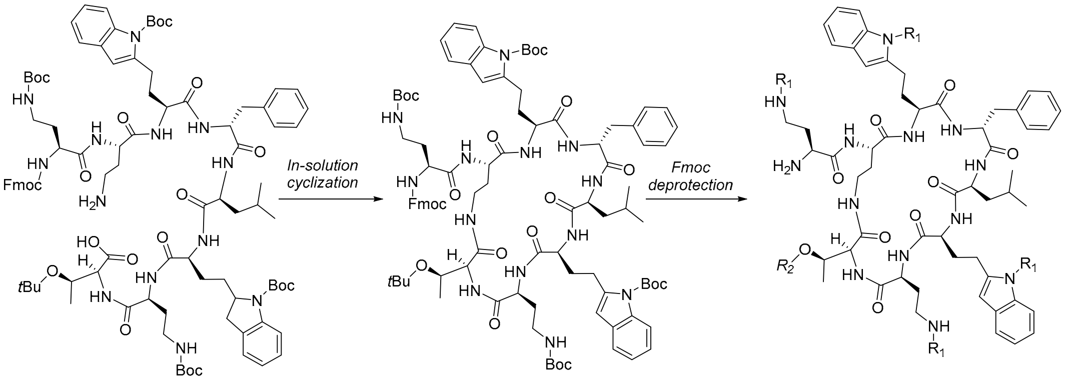

XI **XI'**

prot-8: R_1= Boc; R_2 = *t*Bu → **Monomer 8:** R_1, R_2= H (Global Deprotection)

Solid Phase Synthesis. The synthesis of linear peptide **XI** was performed by following the general procedure A. Peptide couplings were performed using Fmoc-Dab(Boc)-OH (36.9 mg, 0.084 mmol, 1.5 eq), Fmoc-Trp(Boc)-OH (44.2 mg, 0.084 mmol, 1.5 eq), Fmoc-Leu-OH (29.7 mg, 0.084 mmol, 1.5 eq), Fmoc-D-Phe-OH (32.5 mg, 0.084, 1.5 eq), Fmoc-Trp(Boc)-OH (44.2 mg, 0.084 mmol, 1.5 eq), Fmoc-Dab(Dde)-OH (41.2 mg, 0.084 mmol, 1.5 eq). The resin was cleaved affording the linear peptide **XI** (99.0 mg, AcOH salt, 99% yield) as a colourless glassy solid, which was used as such in the subsequent step without further purification. MS (ES^+) *m/z* 1731.9 $[M+H]^+$.

In-solution cyclization. The cyclization was performed by following the general procedure A, starting from linear peptide **XI** (99.0 mg, 0.055 mmol, 1 eq). After the purification step, the protected cyclopeptide **XI'** was obtained as a colourless glassy solid (66.0 mg, 70 % yield). MS (ES^+) *m/z* 1741.0 $[M+H]^+$.

Fmoc deprotection: Compound **XI'** was deprotected following the general procedure A. After the purification step, the cyclopeptide **prot-8** was obtained as a colourless glassy solid (45.6 mg, TFA salt, 74% yield). MS (ES^+) *m/z* 1491.8 $[M+H]^+$. ^{1}H NMR (600 MHz), CD_3OD: δ 8.10 (m, 2H), 7.67 (m, 3H), 7.38-7.18 (m, 8H), 7.04 (m, 2H), 4.77 (m, 1H), 4.55 (m, 1H), 4.32-4.18 (m, 2H), 4.09 (m, 1H), 4.02-3.90 (m, 2H), 3.53 (m, 2H), 3.30-3.09 (m, 8H), 2.76 (m, 1H), 2.59 (m, 1H), 2.19-1.88 (m, 6H), 1.65 (s, 9H), 1.60 (s, 9H), 1.45 (s, 9H), 1.43 (s, 9H), 1.26 (d, J = 5.0 Hz, 3H), 1.16 (m, 9H), 0.63 (m, 3H), 0.57 (m, 3H).

Global deprotection: Compound **prot-8** (8.3 mg, 0.0074 mmol, 1 eq) was deprotected by following the general procedure A. After the purification step, cyclopeptide **BS1-monomer 8** was obtained as a colourless glassy solid (5.3 mg, 68%). HRMS (ES^+) *m/z* calcd for $C_{53}H_{73}N_{13}O_9^{2+}$ 517.7749 $[M+2H]^{2+}$, found 517.7852. ^{1}H NMR (600 MHz), CD_3OD: δ 7.67 (d, *J* = 7.8 MHZ, 1H), 7.52 (d, *J* = 7.8 MHz, 1H), 7.31 (t, *J* = 7.8 MHz, 2H), 7.21 (m, 3H), 7.15-6.93 (m, 8H), 4.57 (t, *J* = 7.1 MHz, 1H), 4.29 (m, 1H), 4.26 (m, 1H), 4.20 (m, 1H), 4.07-3.94 (m, 5H), 3.65 (m, 1H), 3.47 (d, *J* = 6.1 MHz, 2H), 3.23-2.94 (m, 6H), 2.74 (m, 2H), 2.27-15 (m, 4H), 2.12-1.98 (m, 2H), 1.27 (d, *J* = 5.4 MHz, 3H), 1.21 (m, 3H), 0.98 (m, 1H), 0.60 (d, *J* = 7.1 MHz, 3H), 0.54 (m, *J* = 6.1 MHz, 3H).

Synthesis of monomer 12

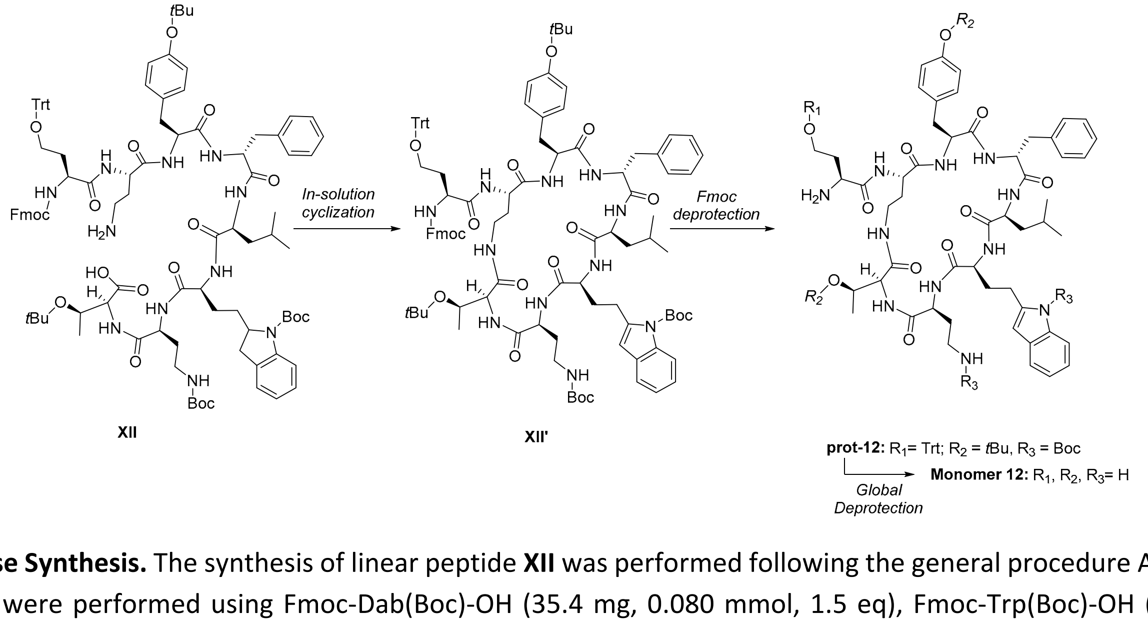

Solid Phase Synthesis. The synthesis of linear peptide **XII** was performed following the general procedure A. Peptide couplings were performed using Fmoc-Dab(Boc)-OH (35.4 mg, 0.080 mmol, 1.5 eq), Fmoc-Trp(Boc)-OH (43.2 mg, 0.080 mmol, 1.5 eq), Fmoc-Leu-OH (28.2 mg, 0.080 mmol, 1.5 eq), Fmoc-D-Phe-OH (31.1 mg, 0.080, 1.5 eq), Fmoc-Tyr(*t*Bu)-OH (36.9 mg, 0.080 mmol, 1.5 eq), Fmoc-Dab(Dde)-OH (39.4 mg, 0.080 mmol, 1.5 eq), Fmoc-Hse(Trt)-OH (45.9, 0.080 mmol, 1.5 eq). The resin was cleaved affording the linear peptide **XII** (79.9 mg, AcOH salt, 80% yield) as a colourless glassy solid, which was used as such in the subsequent step without further purification. MS (ES^+) *m/z* 1823.9 $[M+H]^+$.

In-solution cyclization. The cyclization was performed following the general procedure A, starting from linear peptide **XII** (79.9 mg, 0.043 mmol, 1 eq). After purification, the protected cyclopeptide **XII'** was obtained as a colourless glassy solid (53.7 mg, 70% yield). MS (ES^+) *m/z* 1802.9 $[M+H]^+$.

Fmoc deprotection: Compound **XII'** was deprotected following the general procedure A. After purification, the cyclopeptide **prot-12** was obtained as a colourless glassy solid (45.9 mg, TFA salt, 91% yield). MS (ES$^+$) *m/z* 1581.8 $[M+H]^+$.

Global deprotection: Compound **prot-12** (8.8 mg, 0.005 mmol, 1 eq) was deprotected following the general procedure A. After purification, the cyclopeptide **BS1-monomer 12** was obtained as a colourless glassy solid (2.8 mg, 47%). HRMS (ES$^+$) *m/z* calcd for $C_{51}H_{70}N_{10}O_{12}^{2+}$ 507.2590 $[M+2H]^{2+}$, found 507.2677. ^{1}H NMR (400 MHz) CD_3OD: δ 7.63 (d, *J* = 7.9 Hz, 1H), 7.34 (d, *J* = 7.5 Hz, 1H), 7.30-7.19 (m, 4H), 7.14 (m, 5H), 7.10-7.01 (m, 4H), 6.67 (d, *J* = 85 Hz, 2H), 4.65 (m, 2H), 4.47(m, 1H), 4.37 (m, 1H), 4.14 (m, 2H), 4.10-4.01 (m, 3H), 3.79 (t, *J* = 5.8 Hz, 3H), 3.66 (m, 1H), 3.51 (m, 2H), 3.15-3.81 (m, 8H), 2.30 (m, 1H), 1.42-1.30 (m, 4H), 1.26 (d, *J* = 6.2 Hz, 3H), 0.61(m, 3H), 0.58 (m, 3H).

Synthesis of monomer 15

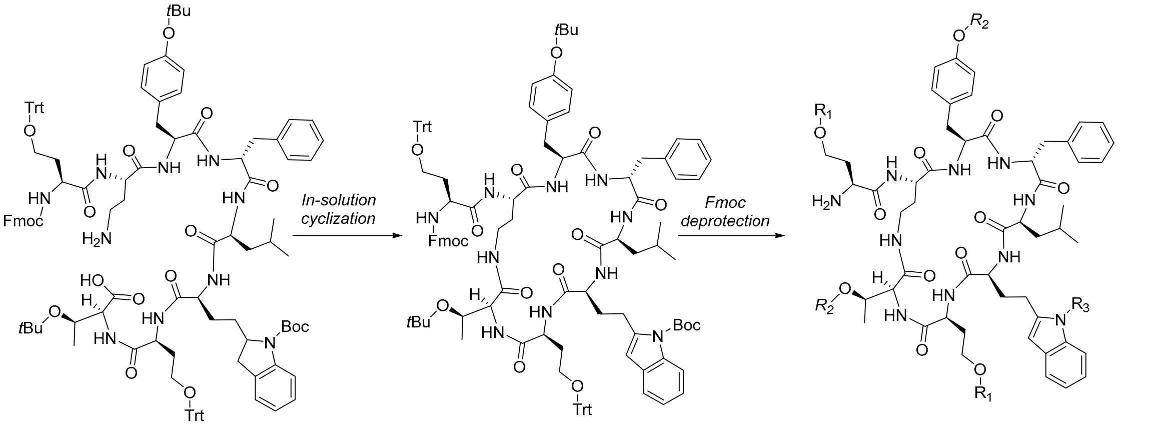

XIII **XIII'**

prot-15: R_1= Trt; R_2 = *t*Bu, R_3 = Boc
→ Global Deprotection **Monomer 15:** R_1, R_2, R_3= H

Solid Phase Synthesis. The synthesis of linear peptide **XIII** was performed by following the general procedure A. Peptide couplings were performed using Fmoc-Hse(Trt)-OH (46.9 mg, 0.080 mmol, 1.5 eq), Fmoc-Trp(Boc)-OH (43.2 mg, 0.080 mmol, 1.5 eq), Fmoc-Leu-OH (28.2 mg, 0.080 mmol, 1.5 eq), Fmoc-D-Phe-OH (31.1 mg, 0.080, 1.5 eq), Fmoc-Tyr(*t*Bu)-OH (36.9 mg, 0.080 mmol, 1.5 eq), Fmoc-Dab(Dde)-OH (39.4 mg, 0.080 mmol, 1.5 eq), Fmoc-Hse(Trt)-OH (45.9 mg, 0.080 mmol, 1.5 eq). The resin was cleaved affording the linear peptide **XIII** (96.3 mg, AcOH salt, 89% yield) as a colourless glassy solid, which was used as such in the subsequent step without further purification. MS (ES^+) *m/z* 1989.1 $[M+Na]^+$.

In-solution cyclization. The cyclization was performed following the general procedure A, starting from linear peptide **XIII** (96.3 mg, 0.048 mmol, 1 eq). After purification, the protected cyclopeptide **XIII'** was obtained as a colourless glassy solid (58.0 mg, 63 % yield). MS (ES^+) *m/z* 1967.9 $[M+Na]^+$.

Fmoc deprotection: Compound **XIII'** was deprotected following the general procedure A. After purification, the cyclopeptide **prot-15** was obtained as a colourless glassy solid (58.9 mg, TFA salt, 63% yield). MS (ES^+) *m/z* 1724.8 $[M+H]^+$.

Global deprotection. Compound **prot-15** (12.0 mg, 0.0071 mmol, 1 eq) was deprotected following the general procedure A. After purification, the cyclopeptide **BS1-monomer 15** was obtained as a colourless glassy solid (5.0 mg, 53%). HRMS (ES^+) *m/z* calcd for $C_{51}H_{71}N_{11}O_{11}^{2+}$ 507.2497 $[M+2H]^{2+}$, found 507.2587. ^1H NMR (400 MHz), CD_3OD: δ 7.63 (d, *J* = 7.7 Hz, 1H), 7.38 (d, *J* = 7.2 Hz, 1H), 7.28-7.19 (m, 4H), 7.14 (m, 3H), 7.03 (m, 3H), 6.67 (d, *J* = 8.8 Hz, 2H), 4.66-4.55 (m, 2H), 4.43 (m, 1H), 4.29 (m, 2H), 4.21 (m, 1H), 4.15-4.03 (m, 3H), 3.78 (t, *J* = 5.4 Hz, 3H), 3.61 (m, 2H), 3.51 (m, 1H), 3.44 (d, *J* = 7.5 Hz, 2 H), 3.11 (m, 1H), 3.01-2.84 (m, 4H), 2.13-1.87 (m, 7H), 1.46-1.30 (m, 6H), 1.22 (d, *J* = 6.9 Hz, 3H), 0.91(m, 1H), 0.68 (m, 3H), 0.62 (m, 3H).

General Procedure B to BS2-targeted monomers as exemplified by the synthesis of monomer 2.

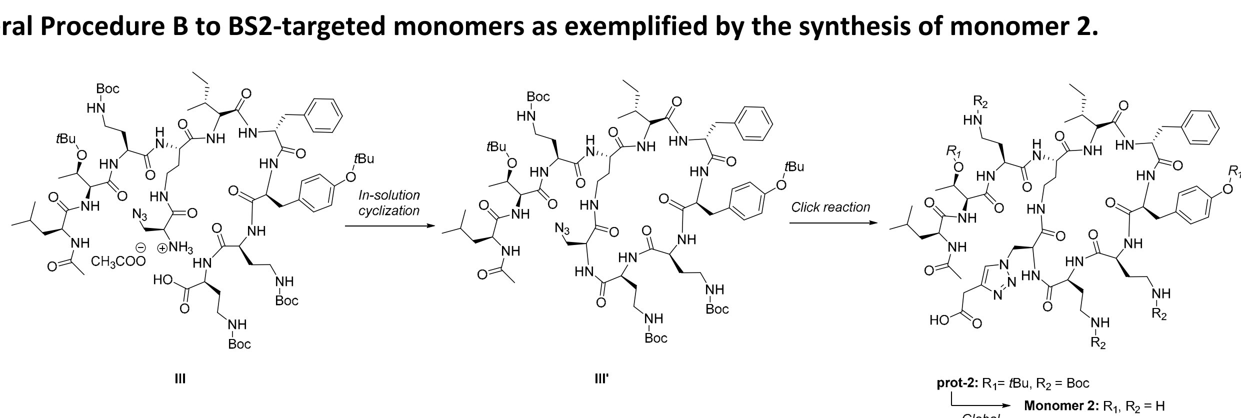

III **III'**

prot-2: R_1= *t*Bu, R_2 = Boc
→ Global Deprotection **Monomer 2:** R_1, R_2 = H

Solid Phase Synthesis. The synthesis of linear peptide **III** (see also Scheme 1 in the main text) was performed following the procedure described for **VI**, starting from the preloaded 2-chlorotrityl-Fmoc-Dab(Boc)-H resin (215.0 mg, resin loading: 0.277 mmol/g, 1 eq). Peptide couplings were performed using Fmoc-Dab(Boc)-OH (39.2 mg, 0.089 mmol, 1.5 eq), Fmoc-Tyr(*t*Bu)-OH (40.9 mg, 0.089 mmol, 1.5 eq), Fmoc-D-Phe-OH (34.5 mg, 0.089 mmol, 1.5 eq), Fmoc-Ile-OH (31.4 mg, 0.089, 1.5 eq), Fmoc-Dab(Dde)-OH (43.7 mg, 0.089 mmol, 1.5 eq), Fmoc-Dab(Boc)-OH (39.2 mg, 0.089 mmol, 1.5 eq), Fmoc-Thr(*t*Bu)-OH (35.4, 0.089 mmol, 1.5 eq), Fmoc-Leu-OH (31.5 mg, 0.089 mmol, 1.5 eq), Fmoc-β-azido-Ala-OH (31.3 mg, 0.089 mmol, 1.5 eq). *Capping procedure:* The capping reaction was performed using 50 eq of acetic anhydride, 50 eq of DIPEA in DMF (1-2 mL). The mixture was left to stir for 30 min and completion of reaction was checked by Kaiser test. *Dde cleavage:* Cleavage of the Dde protecting group was performed using a 4% solution of monohydrated hydrazine in DMF (1-2 mL). The reaction was left to stir for 2.5 h. The linear peptide was cleaved from the resin as described for compound **VI**, affording the linear peptide **III** (75.5 mg, AcOH salt, 76% yield) as a colourless glassy solid, which was used as such in the subsequent step without further purification. MS (ES^+) *m/z* 1624.3 $[M+H]^+$.

In-solution cyclization. The cyclization was performed as described for compound **prot-1**, starting from linear peptide **III** (75.5 mg, 0.049 mmol, 1 eq). After completion, the solution was concentrated under vacuum. The crude product was purified by reverse phase chromatography (eluent: from 90:10 H_2O+0.1%TFA:ACN to 100% ACN) affording the corresponding cyclopeptide **III'** as a colourless glassy solid (53.6 mg, 74% yield). MS (ES^+) *m/z* 1605.2 $[M+H]^+$. ^1H NMR (600 MHz), CD_3OD: δ 7.25-7.03 (m, 7H), 6.87 (m, 2H, ArH), 4.65-4.27 (m, 8H), 4.11 (m, 1H), 4.01 (m, 1H), 3.84-3.67 (m, 2H), 3.25-2.83 (m, 14H), 2.09-1.79 (m, 8H), 1.71-1.56 (m, 4H), 1.41 (m, 27H), 1.28 (m, 9H), 1.18 (m, 9H), 1.09 (m, 3H), 0.93 (m, 3H), 0.89 (m, 3H), 0.75 (m, 3H), 0.58 (m, 3H).

Click reaction: To a solution of the previous compound **III'** (20.5 mg, 0.013 mmol, 1 eq) and 3-butynoic acid (3.2 mg, 0.038 mmol, 3 eq) in DMF (2.4 mL), a solution of $Cu(OAc)_2$ (0.75 mg, 0.004 mmol, 0.3 eq) and sodium ascorbate (1.5 mg, 0.008 mmol, 0.6 eq) in water (1 mL) was added. The reaction was left under stirring under argon atmosphere after 3 cycles of argon/vacuum. After 6 h, the solvent was removed under reduced pressure and the residue was washed with water (3x) and diethyl ether (3x). The crude product was purified by reverse phase chromatography (eluent: from 90:10 H_2O+0.1%TFA:ACN to 100% ACN) affording the cyclopeptide **prot-2** as a colourless glassy solid (12.3 mg, 57% yield). MS (ES^+) *m/z* 1690.2 $[M+H]^+$. ^1H NMR (400 MHz), CD_3OD: δ 7.90 (bs, 1H), 7.28-7.06 (m, 7H), 6.87 (m, 2H), 4.95 (m, 2H), 4.71 (m, 1H), 4.58 (m, 1H), 4.48-4.27 (m, 5H), 4.17 (m, 1H), 3.97 (m, 1H), 3.77 (m, 2H), 3.28-2.87 (m, 12H), 2.09-1.79 (m, 10H), 1.73-1.56 (m, 4H), 1.44 (m, 27H), 1.32 (m, 9H), 1.22 (m, 9H), 1.13 (m, 3H), 1.01-0.93 (m, 6H), 0.79 (m, 3H), 0.60 (m, 3H).

Global deprotection. Compound **prot-2** (5.7 mg, 0.0034 mmol, 1 eq) was treated with a solution of TFA/TIS/H_2O 95:2.5:2.5 (190 μL). After 1 h, the solvent was removed under reduced pressure and the residue was washed with Et_2O (3x). The resulting crude was then purified by reverse phase chromatography (eluent: from 90:10 H_2O+0.1%TFA:ACN to 100% ACN) affording cyclopeptide **BS2-monomer 2** as a colourless glassy solid (2.5 mg, 45%). HRMS (ES^+) *m/z* calcd for $C_{59}H_{72}N_{17}O_{15}^{3+}$ 426.2241 $[M+3H]^{3+}$, found 426.2333. ^1H NMR (600 MHz), CD_3OD: δ 7.89 (bs, 1H), 7.26-7.13 (m, 5H), 7.00 (m, 2H), 6.68 (m, 2H), 4.93 (m, 1H), 4.45-4.17 (m, 8H), 3.74 (m, 2H), 3.13-2.90 (m, 11H), 2.39-2.14 (m, 3H), 2.14-1.87 (m, 8H), 1.72-1.57 (m, 5H), 1.39-1.24 (m, 6H), 1.18 (m, 3H), 0.94 (m, 3H), 0.89 (m, 3H), 0.73 (m, 3H), 0.62 (m, 3H).

Synthesis of monomer 6

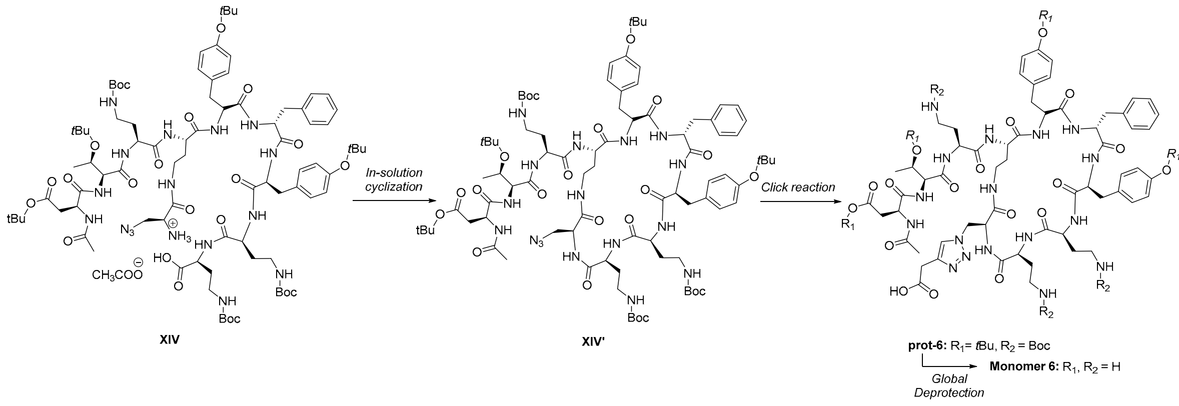

Solid Phase Synthesis. The synthesis of linear peptide **XIV** was performed following the general procedure B. Peptide couplings were performed using Fmoc-Dab(Boc)-OH (35.5 mg, 0.081 mmol, 1.5 eq), Fmoc-Tyr(*t*Bu)-OH (37.0 mg, 0.081 mmol, 1.5 eq), Fmoc-D-Phe-OH (31.2 mg, 0.081 mmol, 1.5 eq), Fmoc-Tyr(*t*Bu)-OH (37.0 mg, 0.081, 1.5 eq), Fmoc-Dab(Dde)-OH (39.5 mg, 0.081 mmol, 1.5 eq), Fmoc-Dab(Boc)-OH (35.5 mg, 0.081 mmol, 1.5 eq), Fmoc-Thr(*t*Bu)-OH (32.0, 0.081 mmol, 1.5 eq), Fmoc-Glu(*t*Bu)-OH (34.3 mg, 0.081 mmol, 1.5 eq), Fmoc-βazido-Ala-OH (28.4 mg, 0.081 mmol, 1.5 eq). The resin was cleaved affording the linear peptide **XIV** (93.4 mg, AcOH salt, 93% yield) as a colourless glassy solid, which was used as such in the subsequent step without further purification. MS (ES$^+$) *m/z* 1801.9 [M+H]$^+$.

In-solution cyclization. The cyclization was performed flowing the general procedure B, starting from linear peptide **XIV** (93.4 mg, 0.050 mmol, 1 eq). After the purification, the protected cyclopeptide **XIV'** was collected as a colourless glassy solid (43.2 mg, 48% yield). MS (ES$^+$) *m/z* 1704.9 [M+H]$^+$.

Click reaction: The click reaction was performed flowing the general procedure B, starting from compound **XIV'** (21.7 mg, 0.012 mmol, 1 eq) and 3-butynoic acid (3.1 mg, 0.037 mmol, 3 eq) in DMF (2.2 mL); a solution of $Cu(OAc)_2$ (0.74 mg, 0.004 mmol, 0.3 eq) and sodium ascorbate (1.4 mg, 0.008 mmol, 0.6 eq) in water (1 mL) was then added. After purification, the cyclopeptide **prot-6** was obtained as a colourless glassy solid (22.2 mg, 97% yield). MS (ES$^+$) *m/z* 1866.2 [M+H]$^+$. ^{1}H NMR (600 MHz), CD_3OD: δ 7.91 (bs, 1H), 7.28-7.14 (m, 3H), 7.05-6.91 (m, 6H), 6.87-6.68 (m, 4H), 4.47-4.28 (m, 8H), 4.12 (m, 2H), 4.01 (m, 1H), 3.79 (m, 1H), 3.71 (m, 1H), 3.43 (m, 1H), 3.25-3.02 (m, 8H), 2.86 (m, 3H), 2.74 (m, 1H), 2.59 (m, 1H), 2.33 (m, 2H), 2.19 (m, 1H), 2.09-1.79 (m, 12H), 1.44 (m, 18H), 1.39 (m, 18H), 1.27 (m, 18H), 1.18 (m, 9H), 1.10 (d, *J* = 6.8 Hz, 3H).

Global deprotection: Compound **prot-6** (6.0 mg, 0.0032 mmol, 1 eq) was deprotected following the general procedure B. After purification, the cyclopeptide **BS2-monomer 6** was obtained as a colourless glassy solid (4.0 mg, 74%). HRMS (ES$^+$) *m/z* calcd for $C_{61}H_{86}N_{17}O_{18}^{3+}$ 448.2034 [M+3H]$^{3+}$, found 448.2140. ^{1}H NMR (600 MHz), CD_3OD: δ 7.88 (bs, 1H), 7.39-7.25 (m, 4H), 7.11 (m, 2H), 6.95 (m, 2H), 6.78 (m, 1H), 6.62 (m, 4H), 4.93 (m, 1H), 4.45-4.17 (m, 10H), 3.72 (m, 2H), 2.56 (m, 1H), 3.13-2.90 (m, 8H), 2.88-2.65 (m, 4H), 2.45 (m, 2H), 2.24 (m, 2H), 2.25-1.85 (m, 11H), 1.39-1.24 (m, 3H), 1.22 (m, 3H).

Synthesis of monomer 9

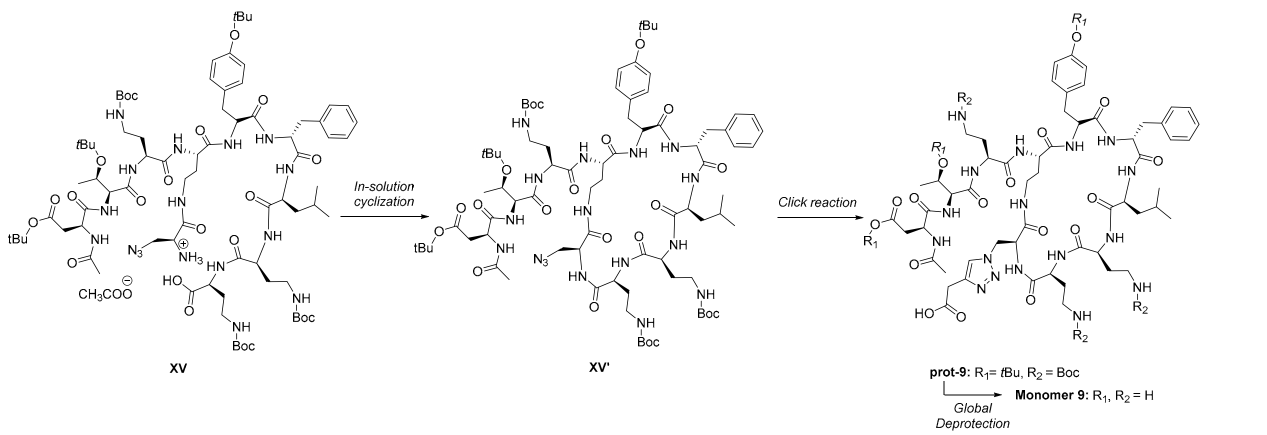

Solid Phase Synthesis. The synthesis of linear peptide **XV** was performed following general procedure B. Peptide couplings were performed using Fmoc-Dab(Boc)-OH (37.7 mg, 0.086 mmol, 1.5 eq), Fmoc-Leu-OH (30.2 mg, 0.086 mmol, 1.5 eq), Fmoc-D-Phe-OH (33.1 mg, 0.086 mmol, 1.5 eq), Fmoc-Tyr(*t*Bu)-OH (39.2 mg, 0.086, 1.5 eq), Fmoc-Dab(Dde)-OH (42.0 mg, 0.086 mmol, 1.5 eq), Fmoc-Dab(Boc)-OH (37.6 mg, 0.086 mmol, 1.5 eq), Fmoc-Thr(*t*Bu)-OH (33.9, 0.086 mmol, 1.5 eq), Fmoc-Glu(*t*Bu)-OH (36.4 mg, 0.086 mmol, 1.5 eq), Fmoc-β-azido-Ala-OH (30.1 mg, 0.086 mmol, 1.5 eq). The resin was cleaved, affording the linear peptide **XV** (99.0 mg, AcOH salt, 99% yield) as a colourless glassy solid, which was used as such in the subsequent step without further purification. MS (ES$^+$) *m/z* 1696.1 [M+H]$^+$.

In-solution cyclization. The cyclization was performed following the general procedure B, starting from linear peptide **XV** (99.0 mg, 0.057 mmol, 1 eq). After purification, the protected cyclopeptide **XV'** was collected as a colourless glassy solid (61.2 mg, 65% yield). MS (ES$^+$) *m/z* 1677.9 [M+H]$^+$. ^{1}H NMR (600 MHz), CD_3OD: δ 7.18 (m, 1H), 7.12-6.93 (m, 5H), 6.87-6.78 (m, 3H), 4.64 (m, 1H), 4.47-4.13 (m, 8H), 3.92 (m, 1H), 3.46 (m, 1H), 3.26-2.97 (m, 10H), 2.94-2.71 (m, 5H), 2.59 (m, 1H), 2.34 (m, 2H), 2.12-1.76 (m, 13H), 1.50-1.36 (m, 34H), 1.26 (m, 18H), 1.18 (m, 9H), 1.11 (m, 3H).

Click reaction: The click reaction was performed following the general procedure B, starting from compound **XV'** (22.4 mg, 0.013 mmol, 1 eq) and 3-butynoic acid (3.4 mg, 0.040 mmol, 3 eq) in DMF (2.5 mL); then, a solution of $Cu(OAc)_2$ (0.79 mg, 0.004 mmol, 0.3 eq) and sodium ascorbate (1.6 mg, 0.008 mmol, 0.6 eq) in water (1.1 mL) was added. After purification, the cyclopeptide **prot-9** was obtained as a colourless glassy solid (20.9 mg, 88% yield). MS (ES$^+$) *m/z* 1762.1 [M+H]$^+$.

Global deprotection. Compound **prot-9** (7.6 mg, 0.0043 mmol, 1 eq) was deprotected following the general procedure B. After purification, the cyclopeptide **BS2-monomer 9** was obtained as a colourless glassy solid (7.6 mg, 68%). HRMS (ES$^+$) *m/z* calcd for $C_{58}H_{87}N_{17}O_{17}^{2+}$ 646.8155 [M+2H]$^{2+}$, found 646.8254. ^{1}H NMR (600 MHz), CD_3OD: δ 7.87 (bs, 1H), 7.25-7.18 (m, 3H), 7.11 (m, 2H), 6.97 (m, 2H), 6.73 (m, 2H), 4.45-4.22 (m, 8H), 4.10 (m, 1H), 3.76 (m, 2H), 3.68 (m, 1H), 3.24-2.78 (m, 13H), 2.51-2.20 (m, 7H), 2.18-1.85 (m, 11H), 1.57 (m, 1H), 1.45 (m, 1H), 1.23 (m, 3H), 0.72 (m, 3H), 0.68 (m, 3H).

Synthesis of monomer 13

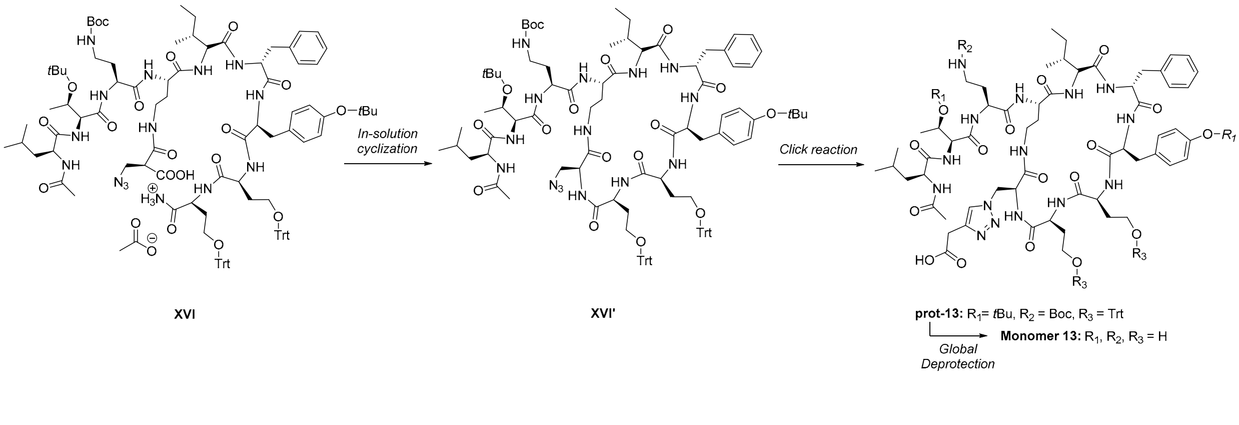

Solid Phase Synthesis. The synthesis of linear peptide **XVI** was performed following the general procedure B. Peptide couplings were performed using Fmoc-Hse(Trt)-OH (70.6 mg, 0.121 mmol, 1.5 eq), Fmoc-Tyr(*t*Bu)-OH (59.9 mg, 0.121 mmol, 1.5 eq), Fmoc-D-Phe-OH (46.9 mg, 0.121 mmol, 1.5 eq), Fmoc-Ile-OH (42.8 mg, 0.121 mmol, 1.5 eq), Fmoc-Dab(Dde)-OH (103.0 mg, 0.121 mmol, 1.5 eq), Fmoc-Dab(Boc)-OH (44.1 mg, 0.121 mmol, 1.5 eq), Fmoc-Thr(*t*Bu)-OH (48.1, 0.121 mmol, 1.5 eq), Fmoc-Leu-OH (42.8 mg, 0.121 mmol, 1.5 eq), Fmoc-β-azido-Ala-OH (42.6 mg, 0.121 mmol, 1.5 eq). The resin was cleaved affording the linear peptide **XVI'** (159.0 mg, AcOH salt, 99% yield) as a colourless glassy solid, which was usedas such in the subsequent step without further purification. MS (ES$^+$) *m/z* 1938.1 [M+H]$^+$.

In-solution cyclization. The cyclization was performed following the general procedure B, starting from linear peptide **XVI** (159.0 mg, 0.081 mmol, 1 eq). After purification, the protected cyclopeptide **XVI'** was obtained as a colourless glassy solid (140.1 mg, 90% yield). MS (ES$^+$) *m/z* 1913.1 [M+Na]$^+$.

Click reaction: The click reaction was performed following the general procedure B, starting from compound **XVI'** (55.0 mg, 0.029 mmol, 1 eq) and 3-butynoic acid (7.3 mg, 0.087 mmol, 3 eq) in DMF (5.2 mL); then, a solution of $Cu(OAc)_2$ (1.7 mg, 0.009 mmol, 0.3 eq) and sodium ascorbate (3.5 mg, 0.0018 mmol, 0.6 eq) in water (2.3 mL) was added. After purification, the cyclopeptide **prot-13** was obtained as a colourless glassy solid (27.2 mg, 47 % yield). MS (ES$^+$) *m/z* 1997.0 [M+Na]$^+$.

Global deprotection: compound **prot-13** (22.3 mg, 0.011 mmol, 1 eq) was deprotected following the general procedure B. After purification, the cyclopeptide **BS2-monomer 13** was obtained as a colourless glassy solid (1.1 mg, 8%). HRMS (ES$^+$) *m/z* calcd for $C_{59}H_{89}N_{15}O_{17}^{2+}$ 639.8202 [M+2H]$^{2+}$, found 639.8286.

General Procedure C to BS1/BS2-targeted heterodimers as exemplified by the synthesis of dimer 3 (see also Scheme 3, main text).

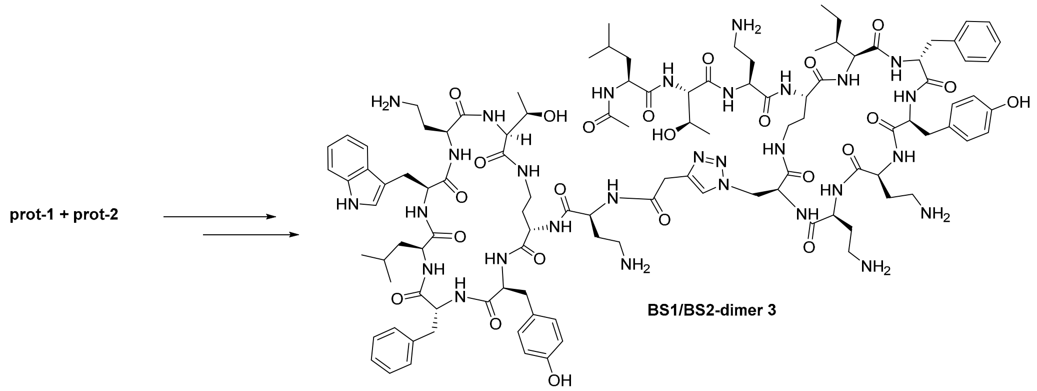

To a solution of compound **prot-2** (6.4 mg, 0.004 mmol, 1 eq), HATU (2.9 mg, 0.008 mmol, 2 eq), HOAt (1.0 mg, 0.008 mmol, 1 eq) and 2,4,6-collidine (1.8 µL, 0.011 mmol, 3 eq) in DMF (0.4 mL), a solution of **prot-1** (7.4 mg, 0.005 mmol, 1.2 eq) in DMF (0.1 mL) was added. The reaction was left stirring at room temperature under nitrogen atmosphere for 6 h. Subsequently, the solvent was removed under reduced pressure and the crude was washed with water (5x). The resulting crude was then treated with a solution of TFA/TIS/H_2O (95:2.5:2.5) (209 µL). After 1 h the solvent was removed under reduced pressure and the solid residue was washed with Et_2O (3x). The resulting crude was purified by reverse phase chromatography (eluent: from 90:10 H_2O+0.1%TFA:ACN to 100% ACN) affording the cyclopeptide **BS1/BS2-dimer 3** as a colourless glassy solid (2.8 mg, 25% yield for 2-steps). HRMS (ES$^+$) *m/z* calcd for $C_{110}H_{161}N_{29}O_{24}^{4+}$ 568.3067 [M+4H]$^{4+}$, found 568.3075.

Synthesis of BS1/BS2-dimer 4

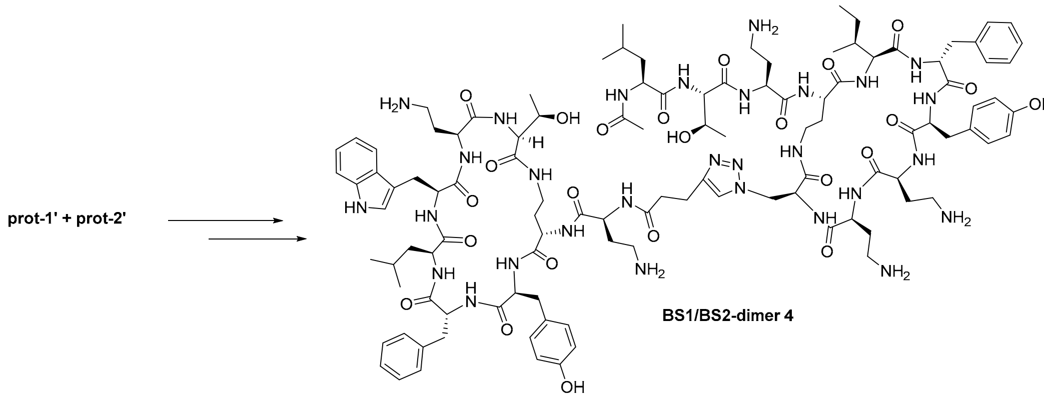

Exact Mass: 2282,2113

Compound **BS1/BS2-dimer 4** was synthesized following the general procedure C, starting from **prot-1'** (9.0 mg, 0.006 mmol, 1 eq) and **prot-2'** (9.6 mg, 0.006 mmol, 1 eq). After purification, the cyclopeptide **BS1/BS2-dimer 4** was obtained as a colourless glassy solid (6.4 mg, 36% 2-steps yield). HRMS (ES^+) *m/z* calcd for $C_{111}H_{163}N_{29}O_{24}^{4+}$ 571.5607 $[M+4H]^{4+}$, found 571.5606.

Synthesis of BS1/BS2-dimer 7

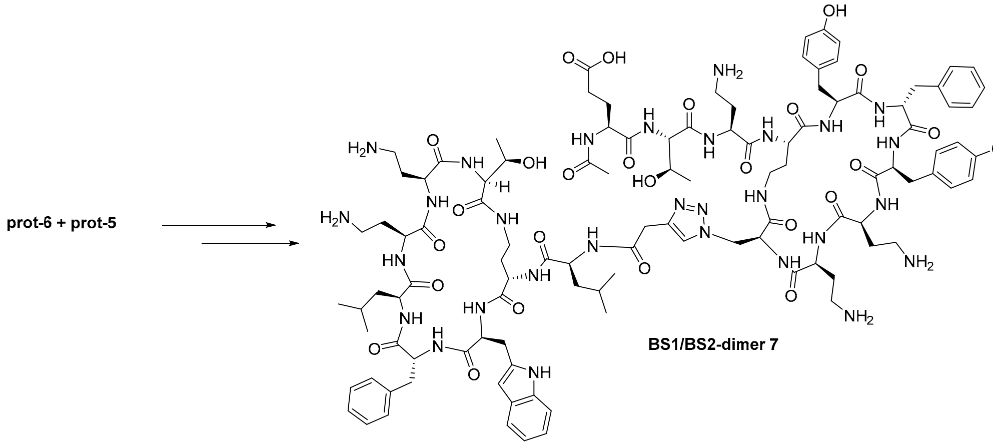

Compound **BS1/BS2-dimer 7** was synthesized following the general procedure C, starting from compound **prot-6** (10.8 mg, 0.006 mmol, 1.1 eq) and **prot-5** (10.8 mg, 0.008 mmol, 1.3 eq). After purification, the cyclopeptide **BS1/BS2-dimer 7** was obtained as a colourless glassy solid (4.6 mg, 27% 2-steps yield). HRMS (ES$^+$) *m/z* calcd for $C_{109}H_{157}N_{29}O_{26}^{4+}$ 572.2964 $[M+4H]^{4+}$, found 572.2990.

Synthesis of BS1/BS2-dimer 10

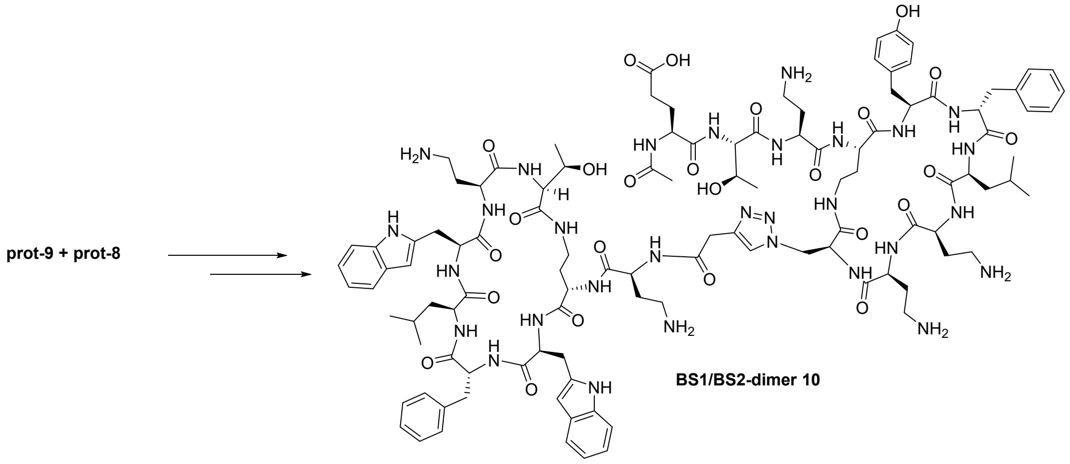

Compound **BS1/BS2-dimer 10** was synthesized following the general procedure C, starting from compound **prot-9** (9.3 mg, 0.005 mmol, 1.1 eq) and **prot-8** (7.4 mg, 0.005 mmol, 1.2 eq). After purification, the cyclopeptide **BS1/BS2-dimer 10** was obtained as a colourless glassy solid (6.4 mg, 45% 2-steps yield). HRMS (ES$^+$) *m/z* calcd for $C_{111}H_{158}N_{30}O_{25}^{4+}$ 578.0504 $[M+4H]^{4+}$, found 578.0528.

Synthesis of BS1/BS2-dimer 14

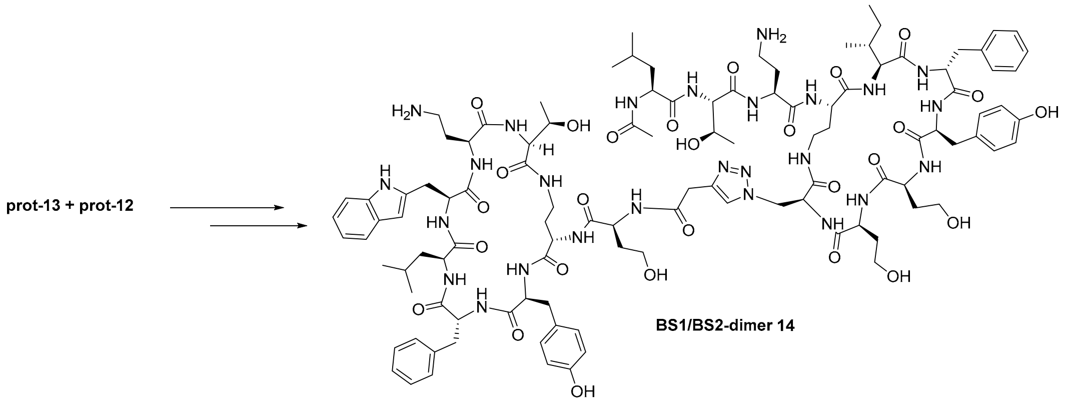

Compound **BS1/BS2-dimer 14** was synthesized following the general procedure C, starting from compound **prot-13** (11.7 mg, 0.005 mmol, 1 eq) and **prot-12** (8.3 mg, 0.005 mmol, 1.2 eq). After purification, the cyclopeptide **BS1/BS2-dimer 14** was obtained as a colourless glassy solid (2.3 mg, 18% 2-steps yield). HRMS (ES$^+$) *m/z* calcd for $C_{110}H_{157}N_{26}O_{27}{}^{3+}$ 758.0570 $[M+3H]^{3+}$, found 758.0576.

Synthesis of BS1/BS2-dimer 16

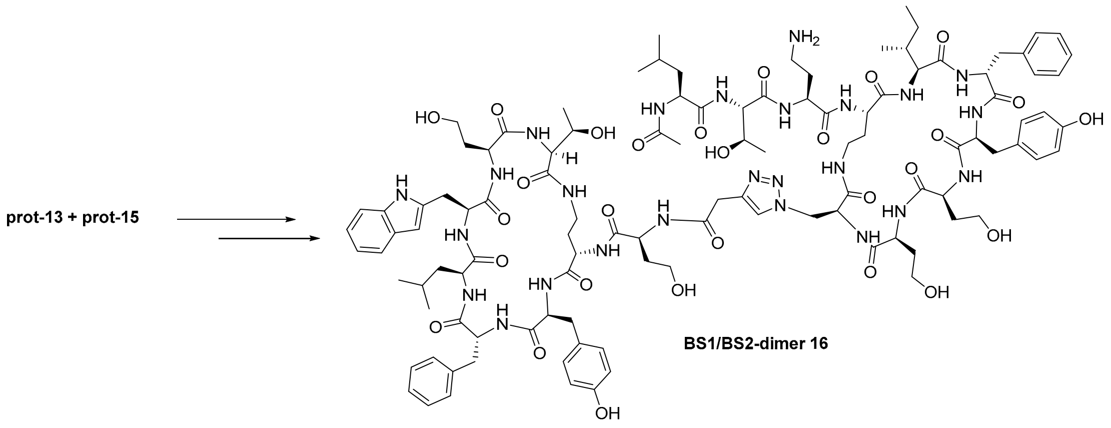

Compoung **BS1/BS2-dimer 16** was synthesized following the general procedure C, starting from **prot-13** (7.5 mg, 0.004 mmol, 1 eq) and **prot-15** (5.8 mg, 0.003 mmol, 1.2 eq). After the purification, cyclopeptide **BS1/BS2-dimer 16** was obtained as a colourless glassy solid (1.7 mg, 21% 2-steps yield). HRMS (ES$^+$) *m/z* calcd for $C_{110}H_{155}N_{25}O_{28}{}^{2+}$ 1137.0737 $[M+2H]^{2+}$, found 1137.0737.

4. HPLC chromatograms

Monomer 1

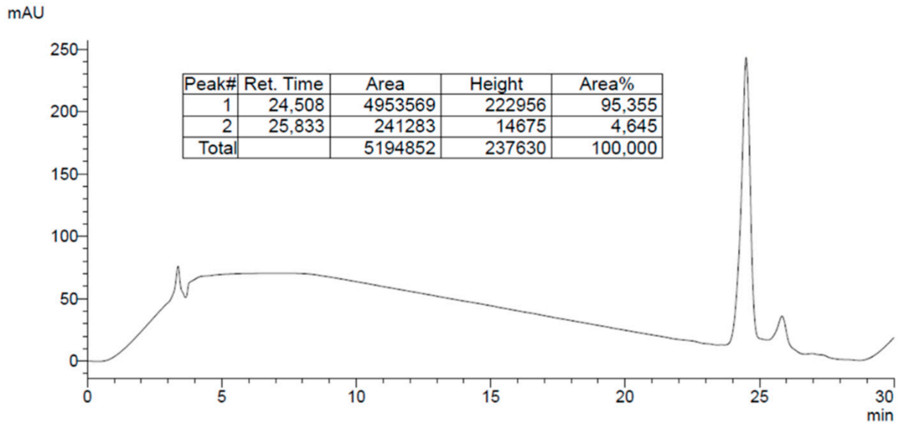

Peak#	Ret. Time	Area	Height	Area%
1	24,508	4953569	222956	95,355
2	25,833	241283	14675	4,645
Total		5194852	237630	100,000

Monomer 5

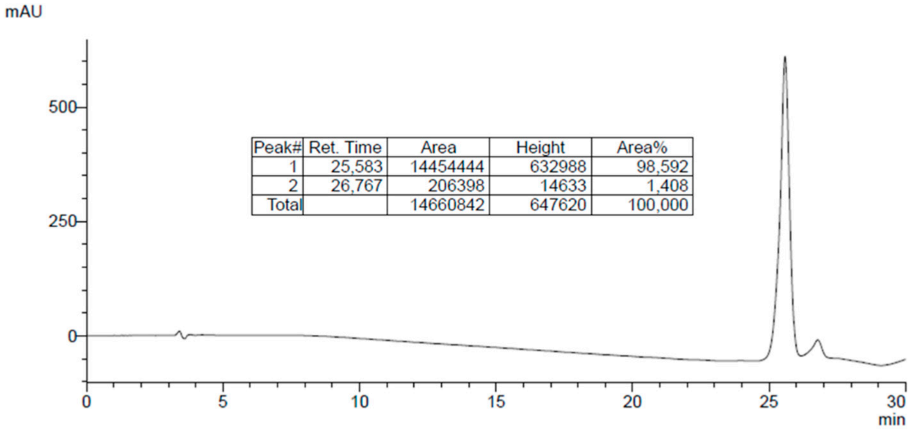

Peak#	Ret. Time	Area	Height	Area%
1	25,583	14454444	632988	98,592
2	26,767	206398	14633	1,408
Total		14660842	647620	100,000

Monomer 8

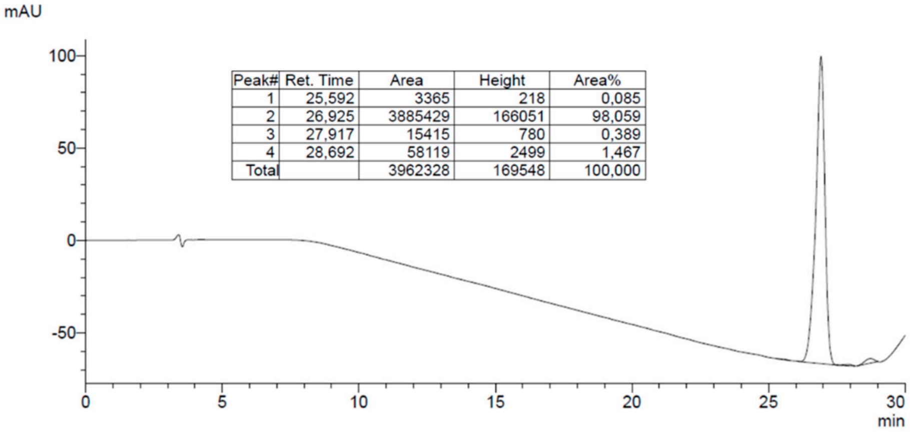

Peak#	Ret. Time	Area	Height	Area%
1	25,592	3365	218	0,085
2	26,925	3885429	166051	98,059
3	27,917	15415	780	0,389
4	28,692	58119	2499	1,467
Total		3962328	169548	100,000

Monomer 12

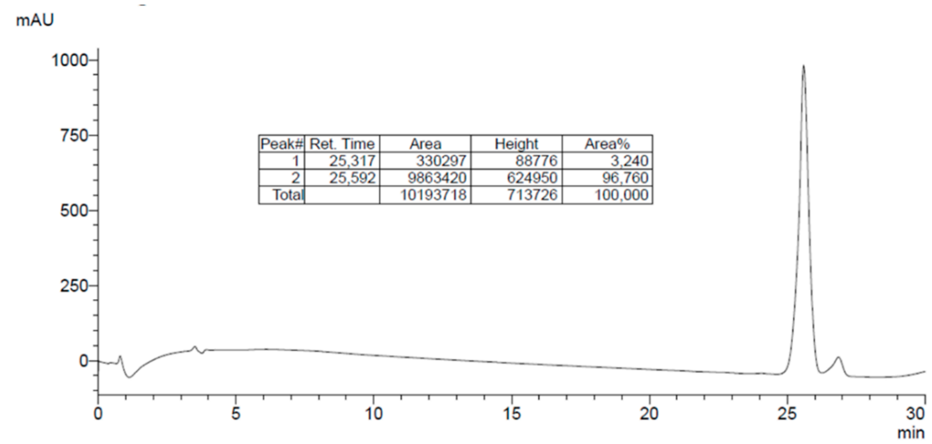

Peak#	Ret. Time	Area	Height	Area%
1	25,317	330297	88776	3,240
2	25,592	9863420	624950	96,760
Total		10193718	713726	100,000

Monomer 15

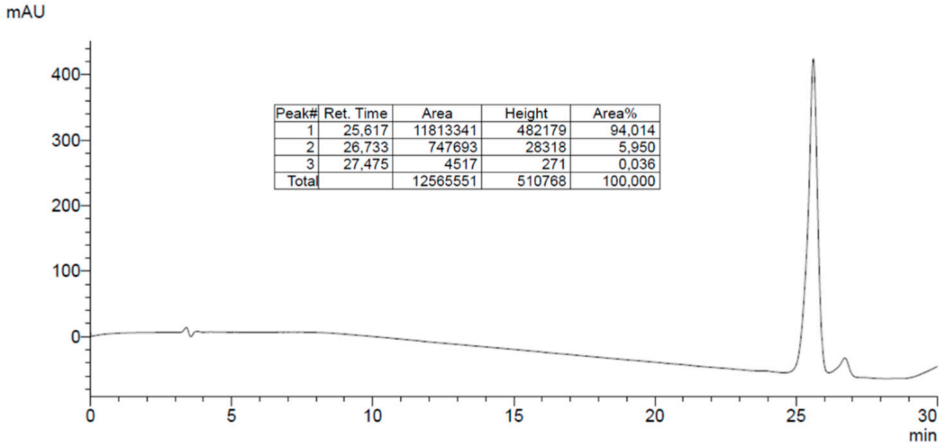

Peak#	Ret. Time	Area	Height	Area%
1	25,617	11813341	482179	94,014
2	26,733	747693	28318	5,950
3	27,475	4517	271	0,036
Total		12565551	510768	100,000

Monomer 2

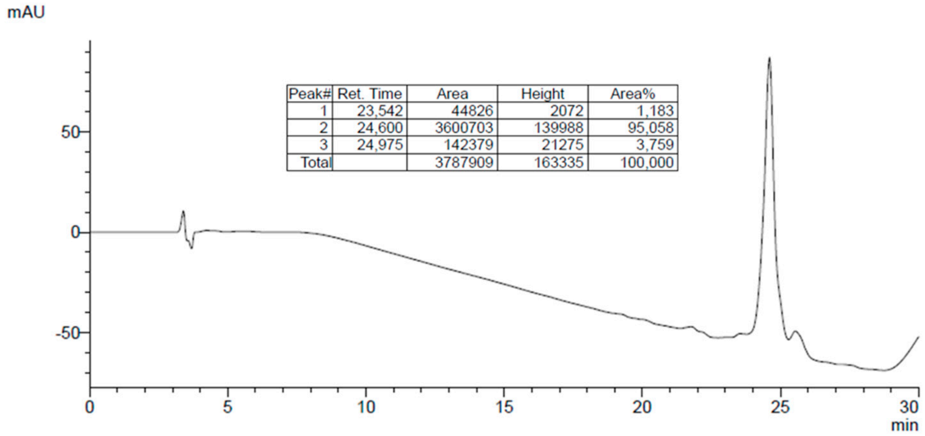

Peak#	Ret. Time	Area	Height	Area%
1	23,542	44826	2072	1,183
2	24,600	3600703	139988	95,058
3	24,975	142379	21275	3,759
Total		3787909	163335	100,000

Monomer 6

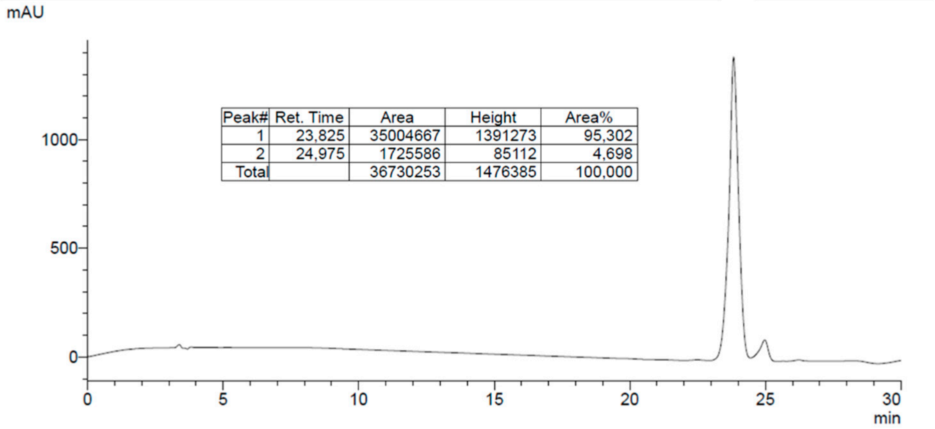

Peak#	Ret. Time	Area	Height	Area%
1	23,825	35004667	1391273	95,302
2	24,975	1725586	85112	4,698
Total		36730253	1476385	100,000

Monomer 9

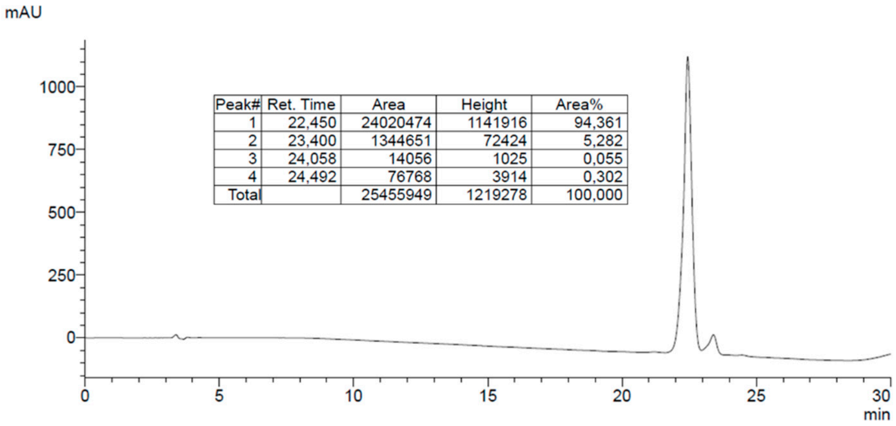

Peak#	Ret. Time	Area	Height	Area%
1	22,450	24020474	1141916	94,361
2	23,400	1344651	72424	5,282
3	24,058	14056	1025	0,055
4	24,492	76768	3914	0,302
Total		25455949	1219278	100,000

Monomer 13

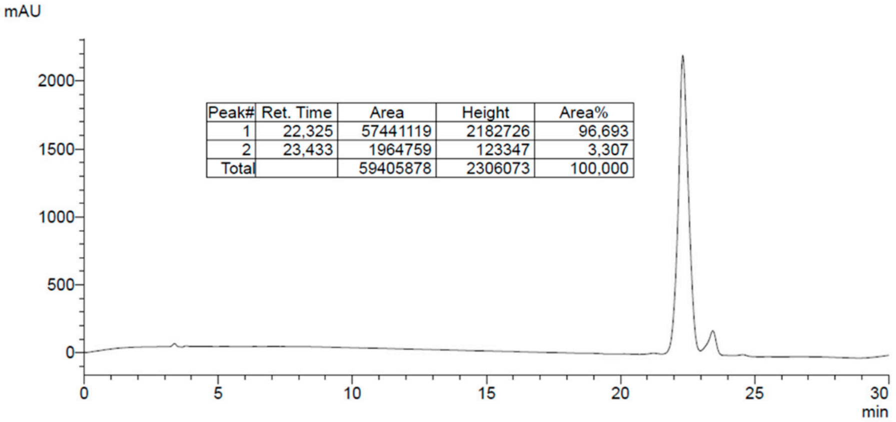

Peak#	Ret. Time	Area	Height	Area%
1	22,325	57441119	2182726	96,693
2	23,433	1964759	123347	3,307
Total		59405878	2306073	100,000

Dimer 3

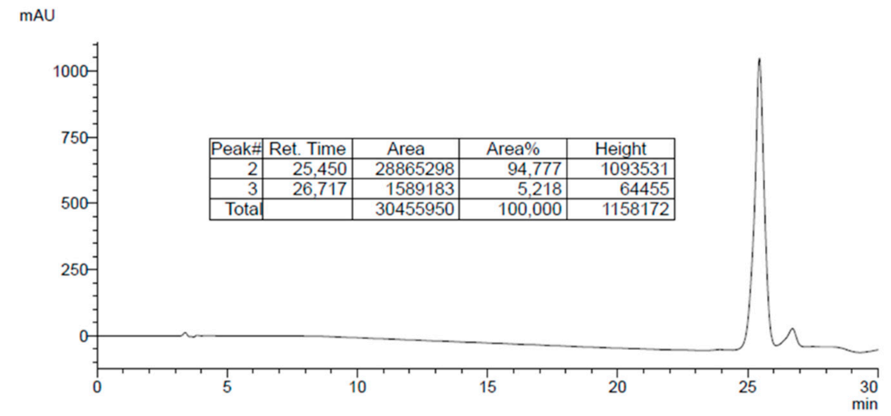

Peak#	Ret. Time	Area	Area%	Height
2	25,450	28865298	94,777	1093531
3	26,717	1589183	5,218	64455
Total		30455950	100,000	1158172

Dimer 4

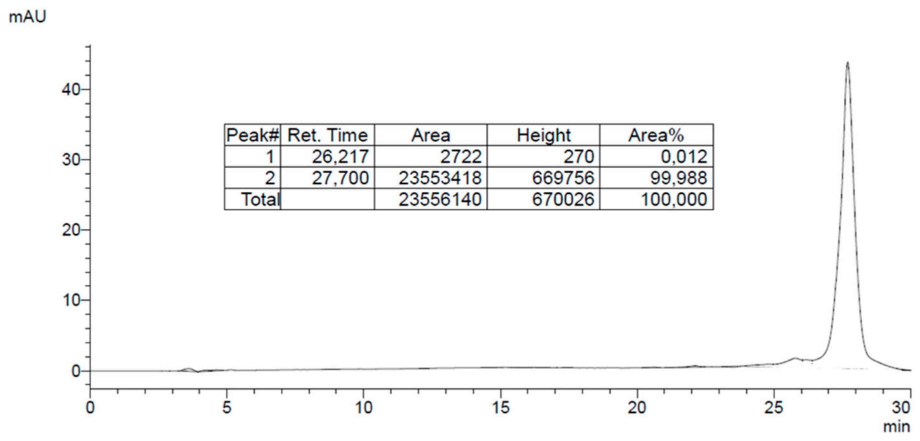

Peak#	Ret. Time	Area	Height	Area%
1	26,217	2722	270	0,012
2	27,700	23553418	669756	99,988
Total		23556140	670026	100,000

Dimer 7

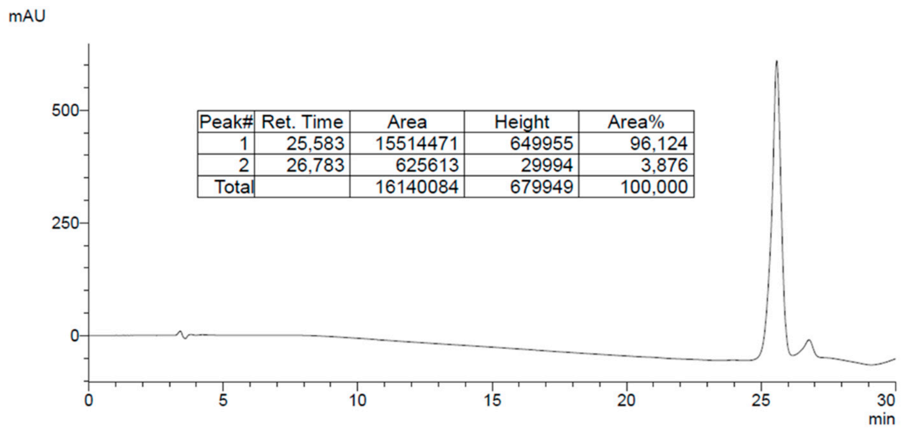

Peak#	Ret. Time	Area	Height	Area%
1	25,583	15514471	649955	96,124
2	26,783	625613	29994	3,876
Total		16140084	679949	100,000

Dimer 10

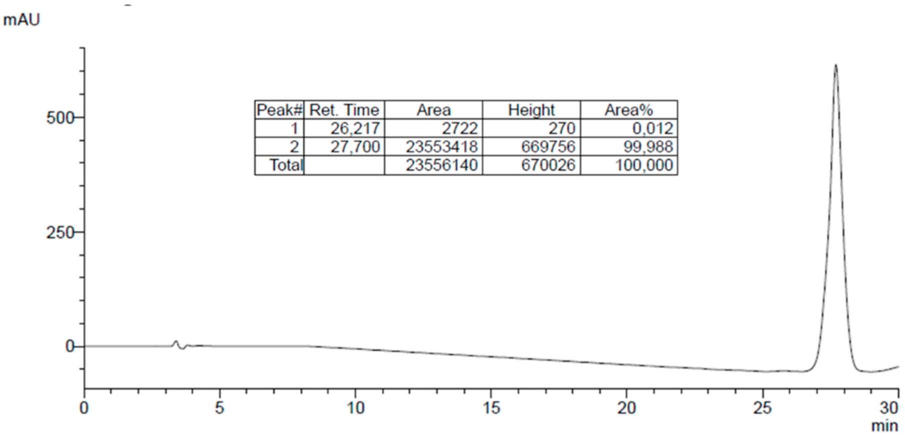

Peak#	Ret. Time	Area	Height	Area%
1	26,217	2722	270	0,012
2	27,700	23553418	669756	99,988
Total		23556140	670026	100,000

Dimer 14

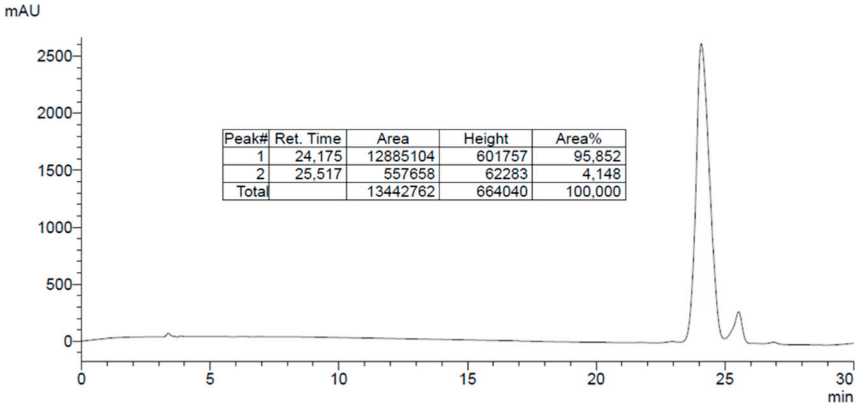

Peak#	Ret. Time	Area	Height	Area%
1	24,175	12885104	601757	95,852
2	25,517	557658	62283	4,148
Total		13442762	664040	100,000

Dimer 16

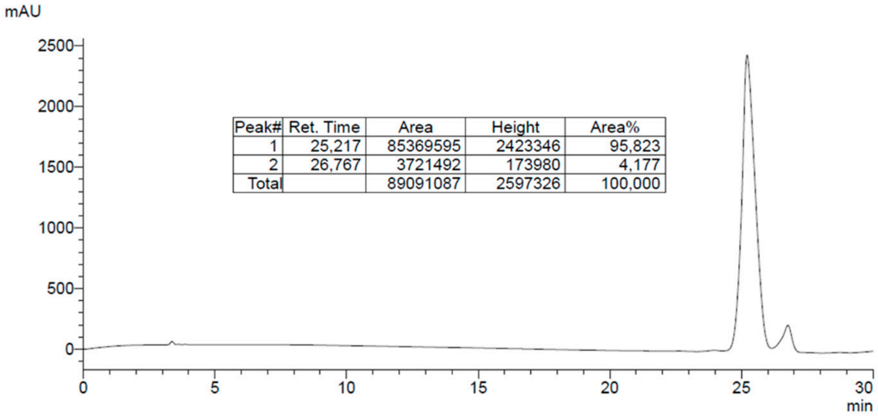

Peak#	Ret. Time	Area	Height	Area%
1	25,217	85369595	2423346	95,823
2	26,767	3721492	173980	4,177
Total		89091087	2597326	100,000

5. NMR spectra

Monomer 1, ^{1}H NMR, 600 MHz, CD_3OD

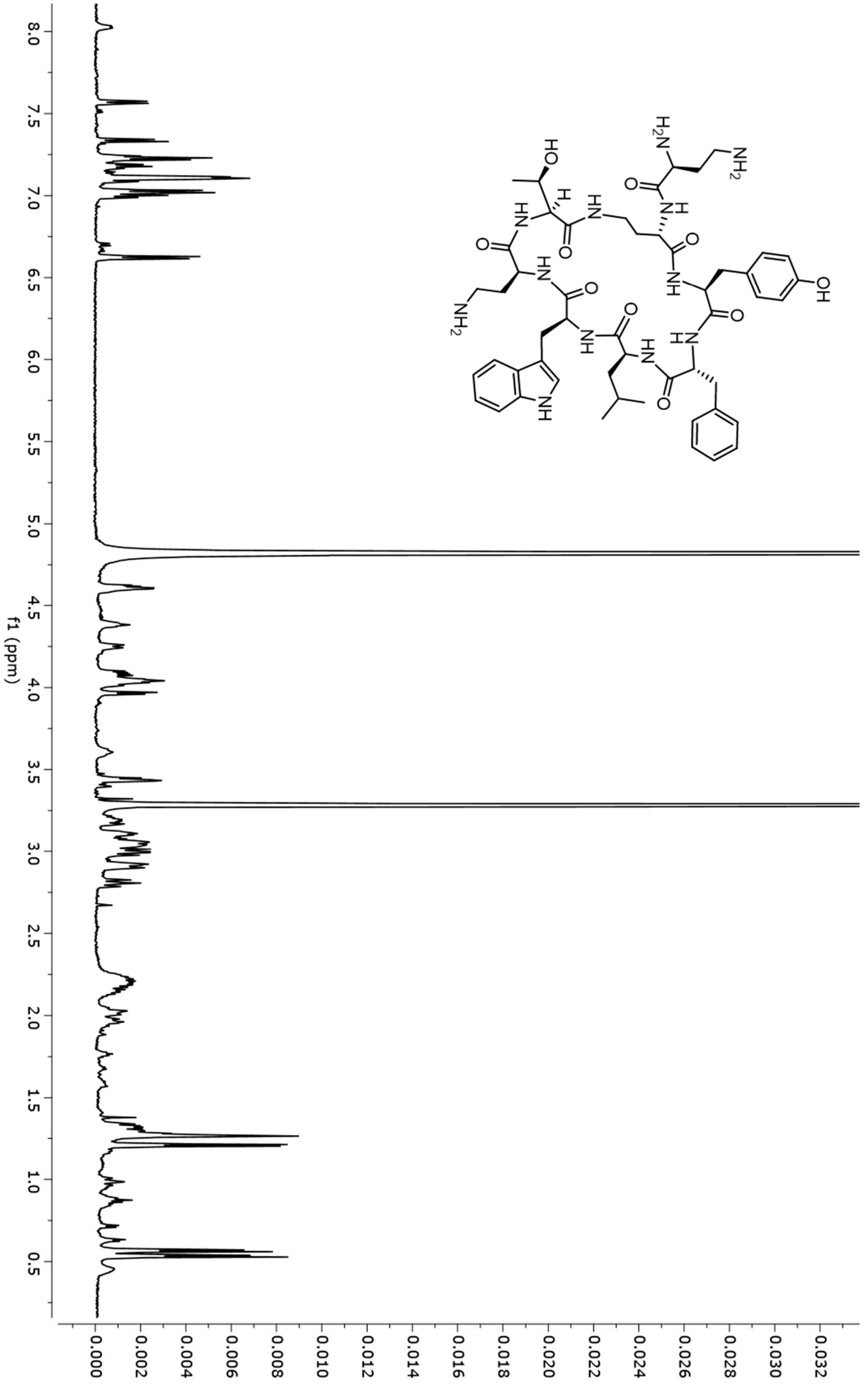

Monomer 5, ^{1}H NMR, 600 MHz, CD_3OD

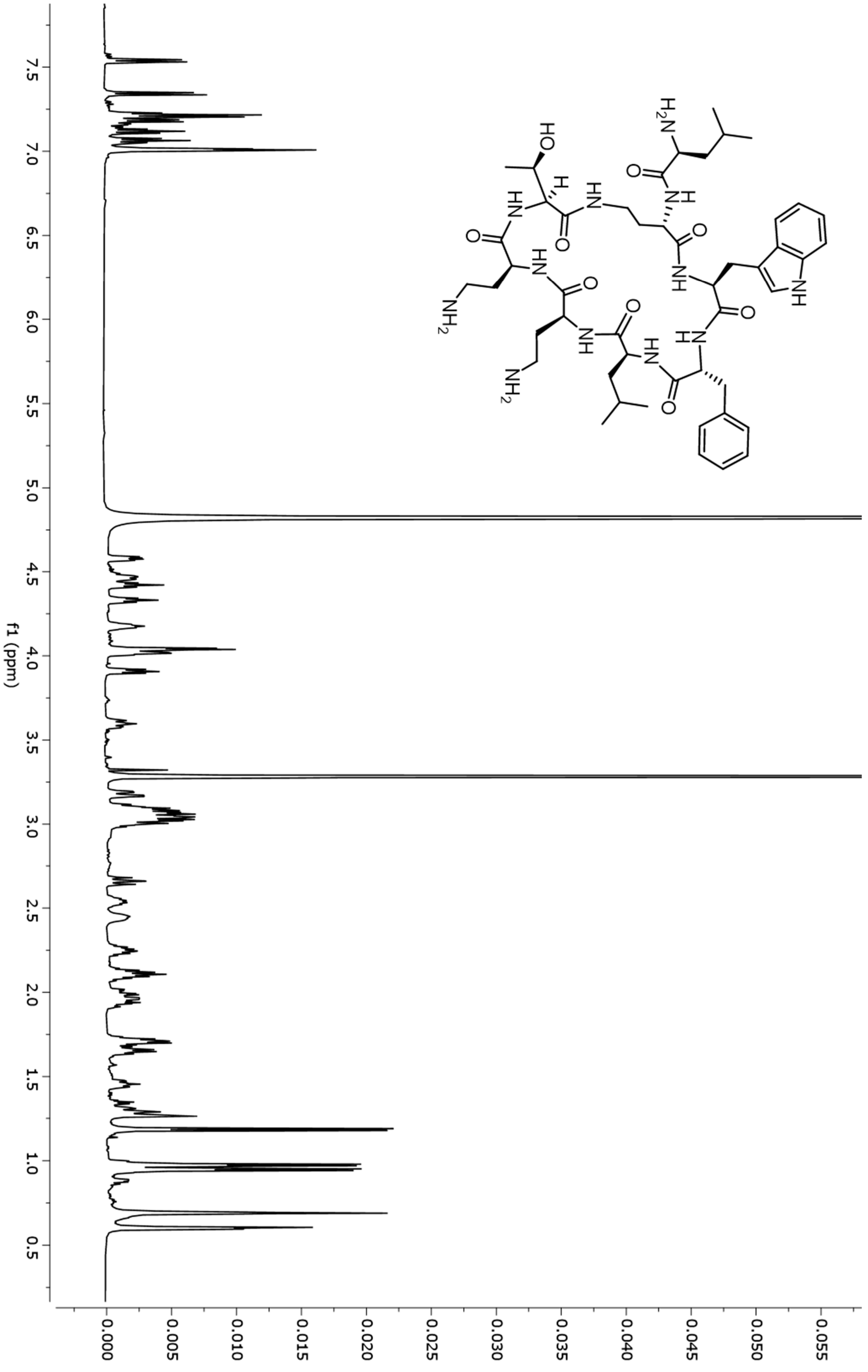

Monomer 8, ^{1}H NMR, 600 MHz, CD_3OD

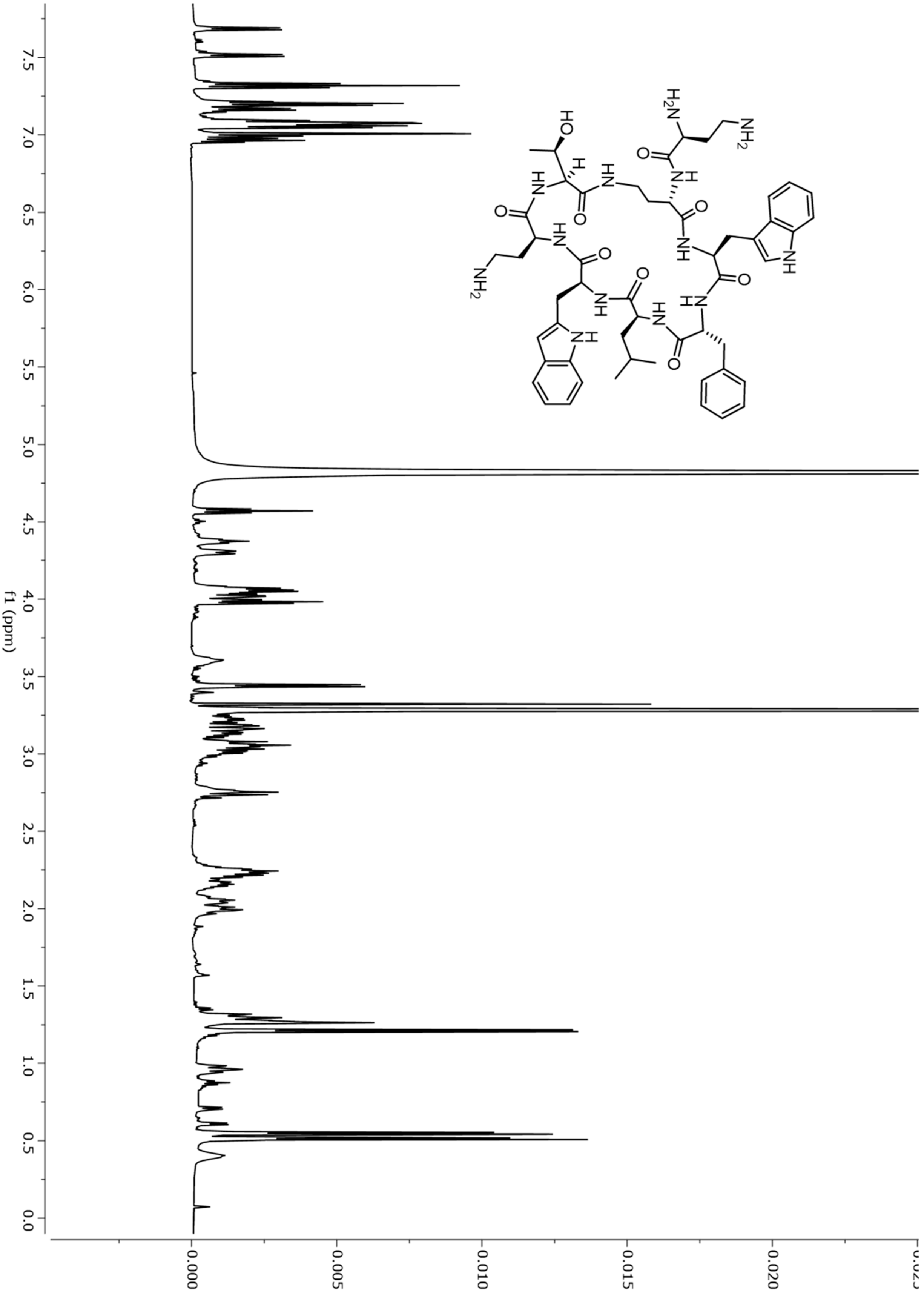

Monomer 12, ^{1}H NMR, 400 MHz, CD_3OD

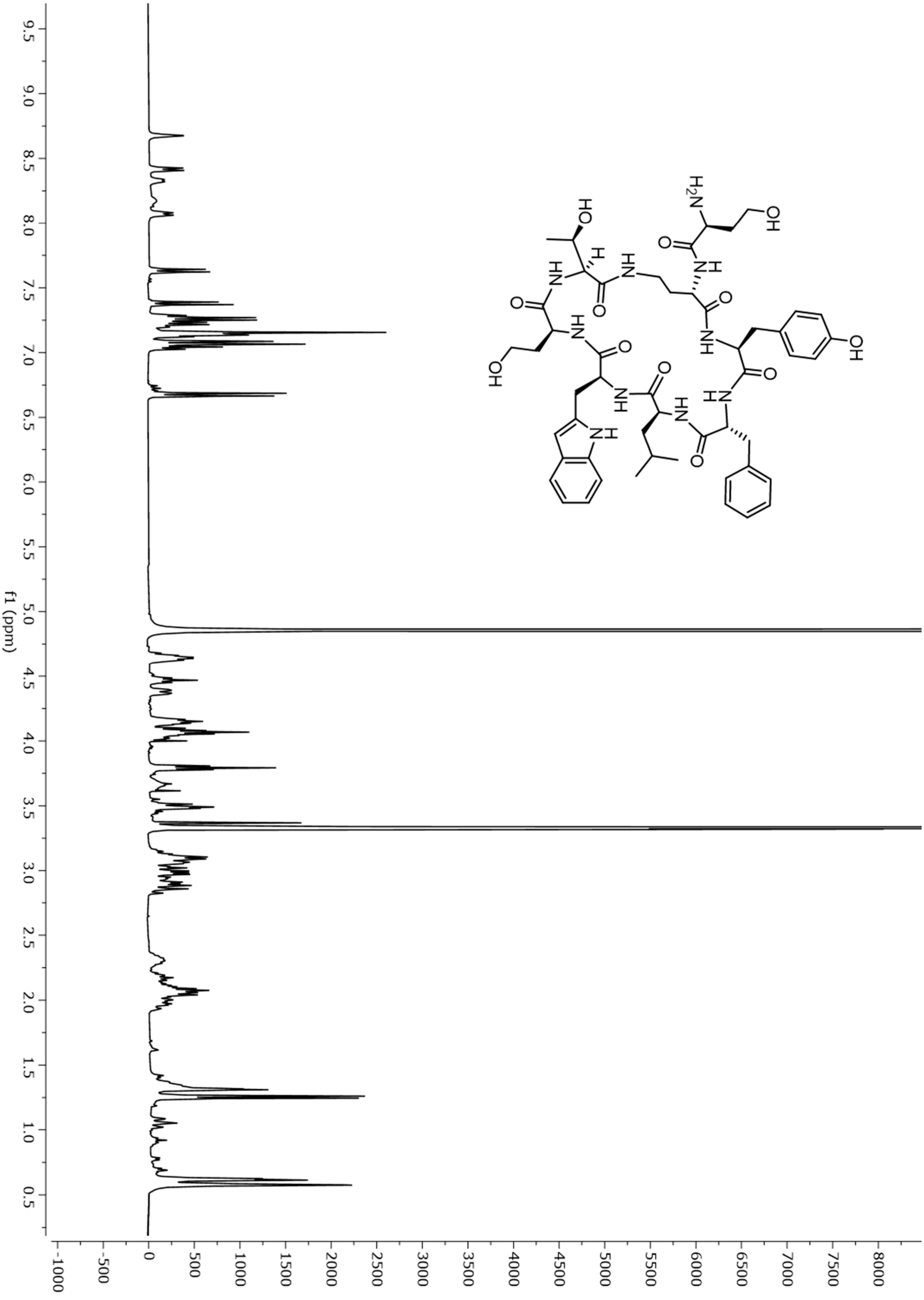

Monomer 15, ^{1}H NMR, 400 MHz, CD_3OD

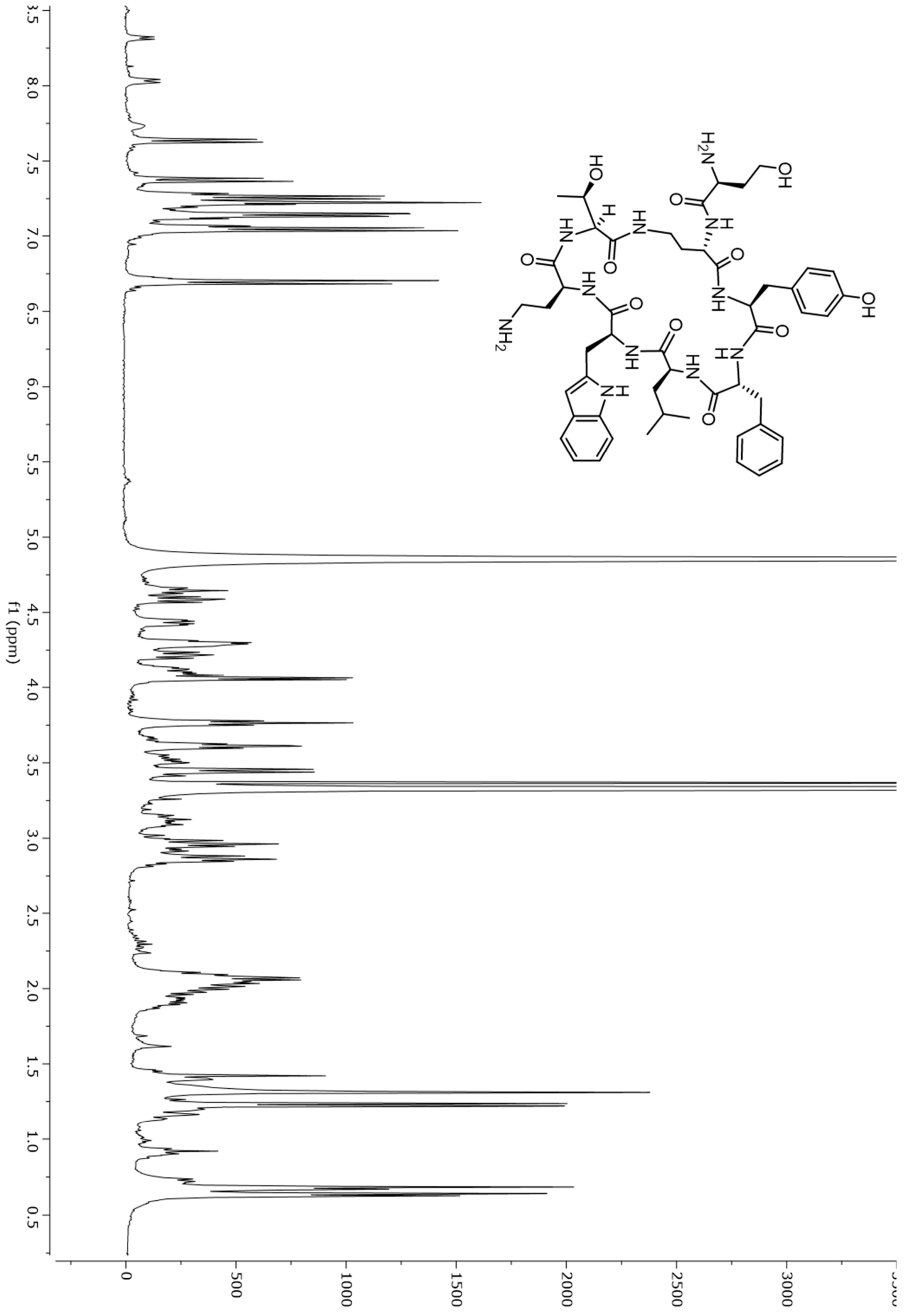

Monomer 2, ^1H NMR, 600 MHz, CD_3OD

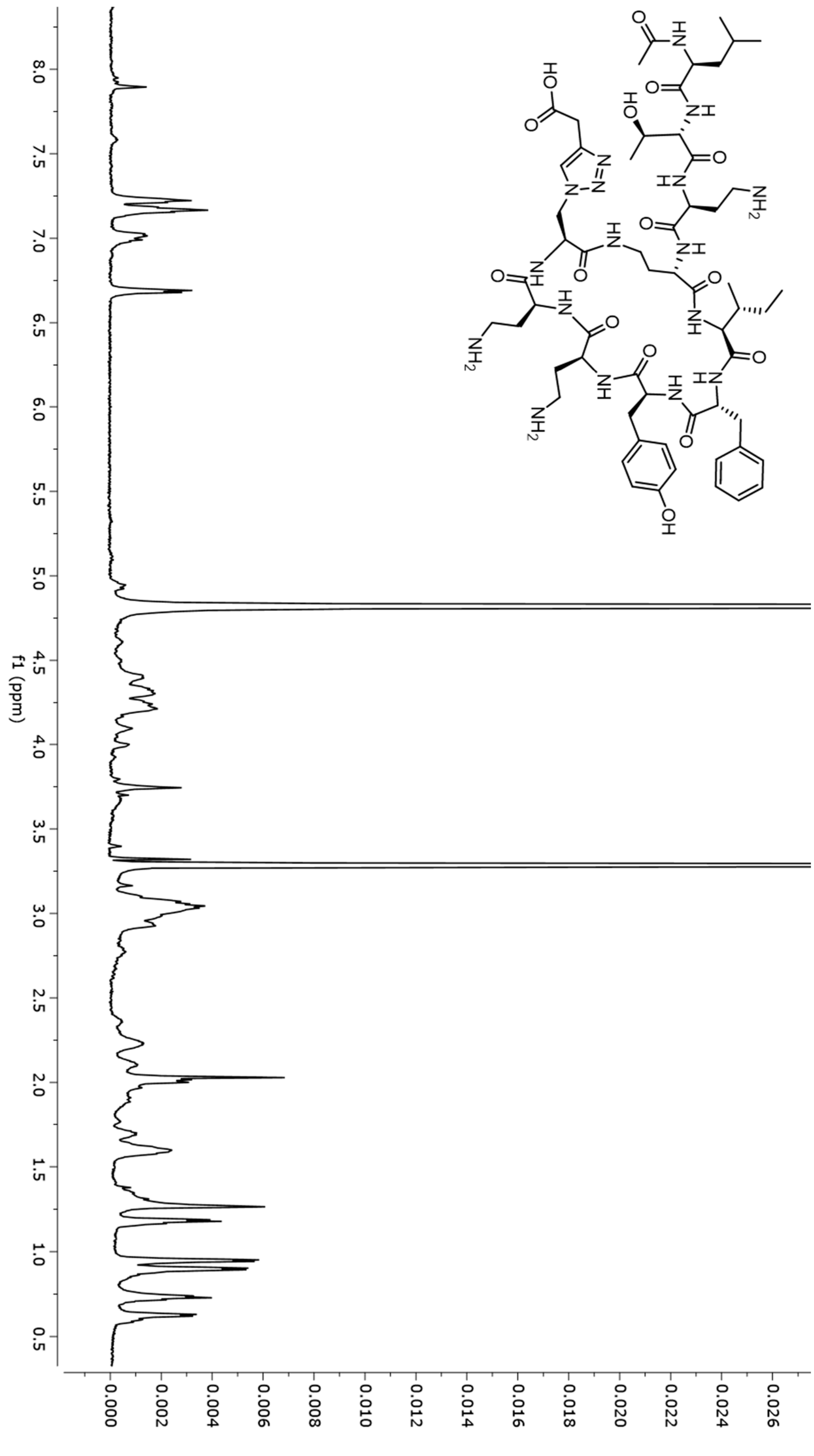

Monomer 6, ^{1}H NMR, 600 MHz, CD_3OD

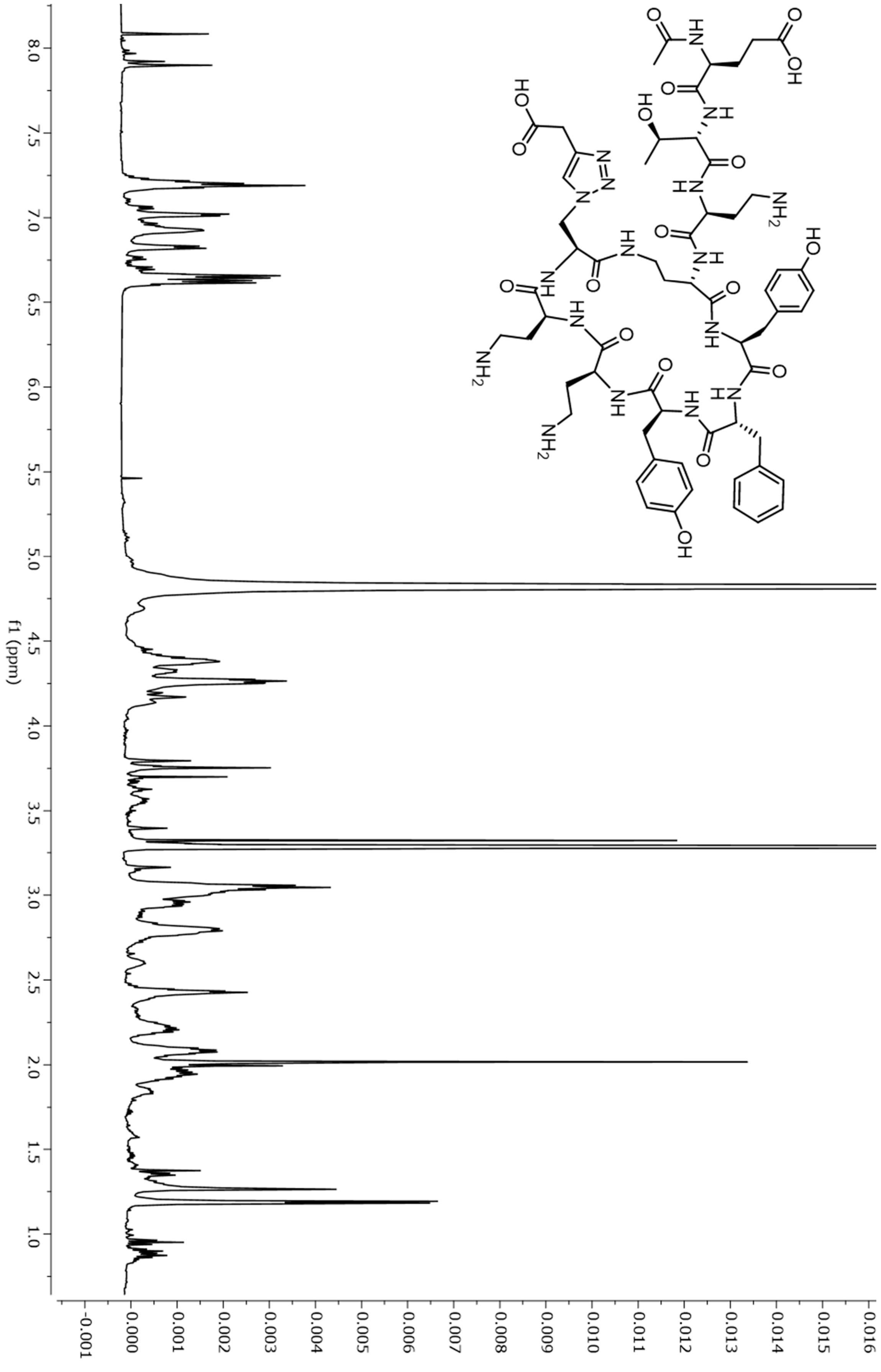

Monomer 9, ^{1}H NMR, 600 MHz, CD_3OD

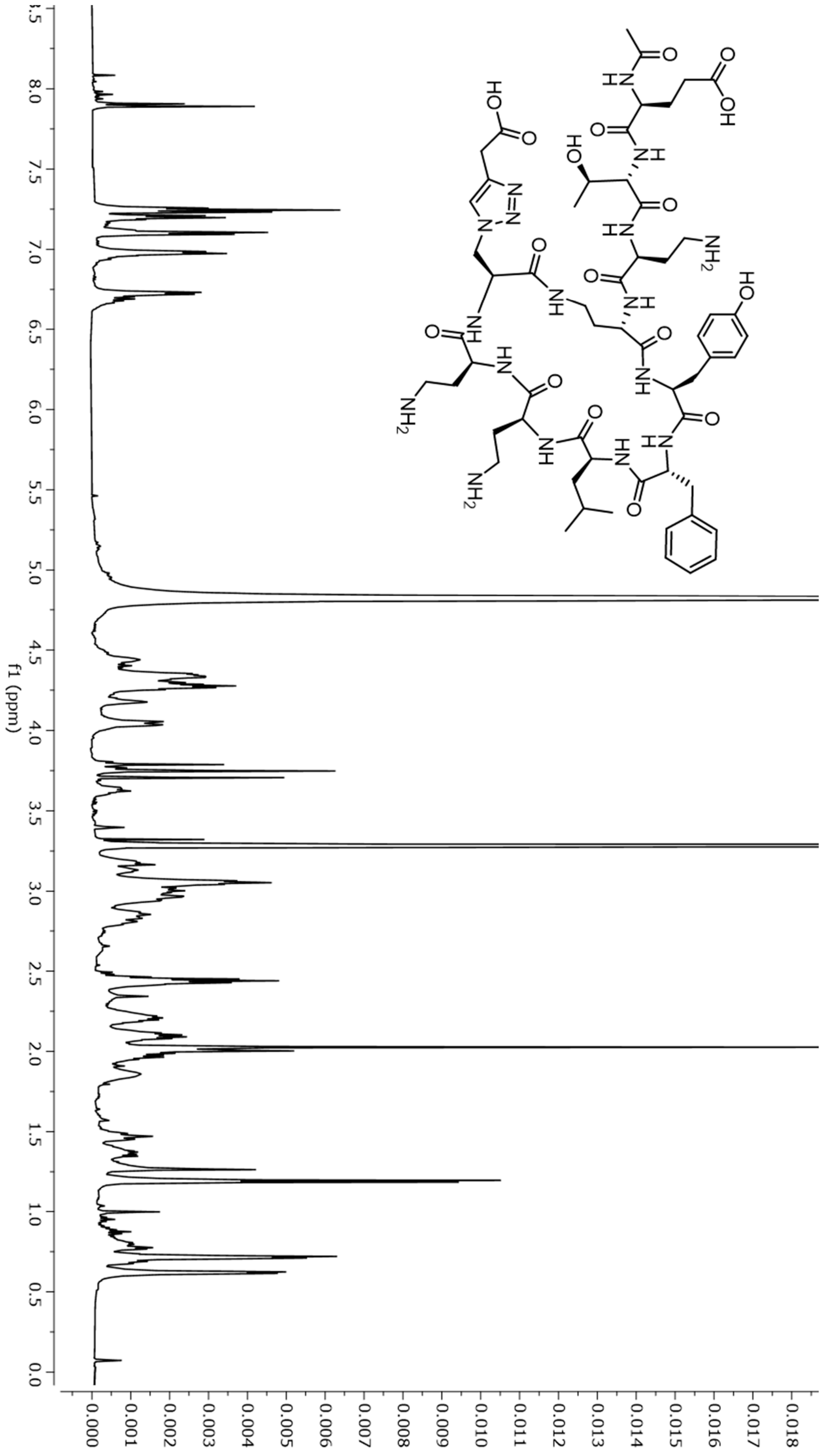

Monomer 13, ^{1}H NMR, 400 MHz, CD_3OD

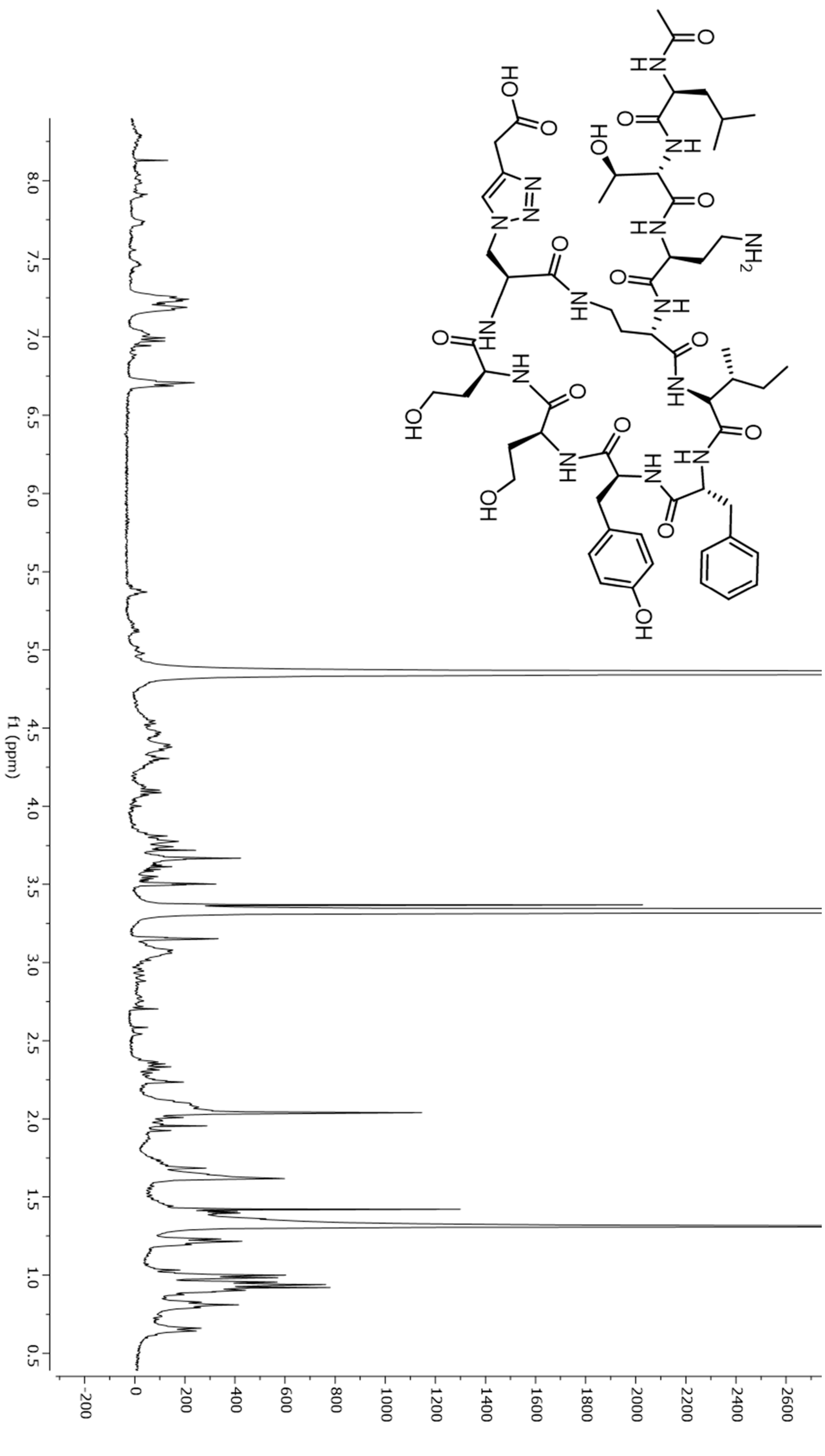

Dimer 3, ^{1}H NMR, 600 MHz, CD_3OD

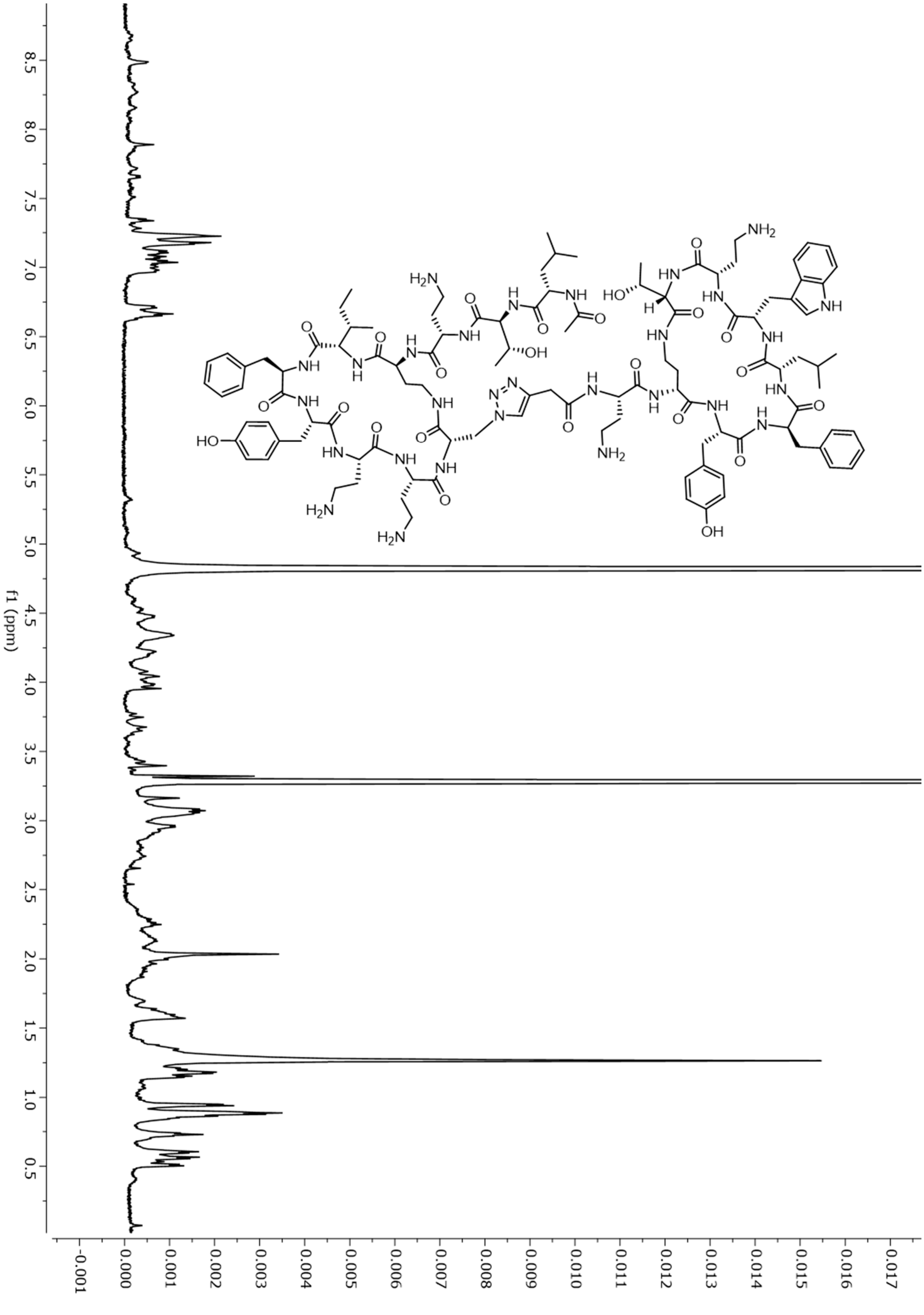

Dimer 4, ^{1}H NMR, 600 MHz, CD_3OD

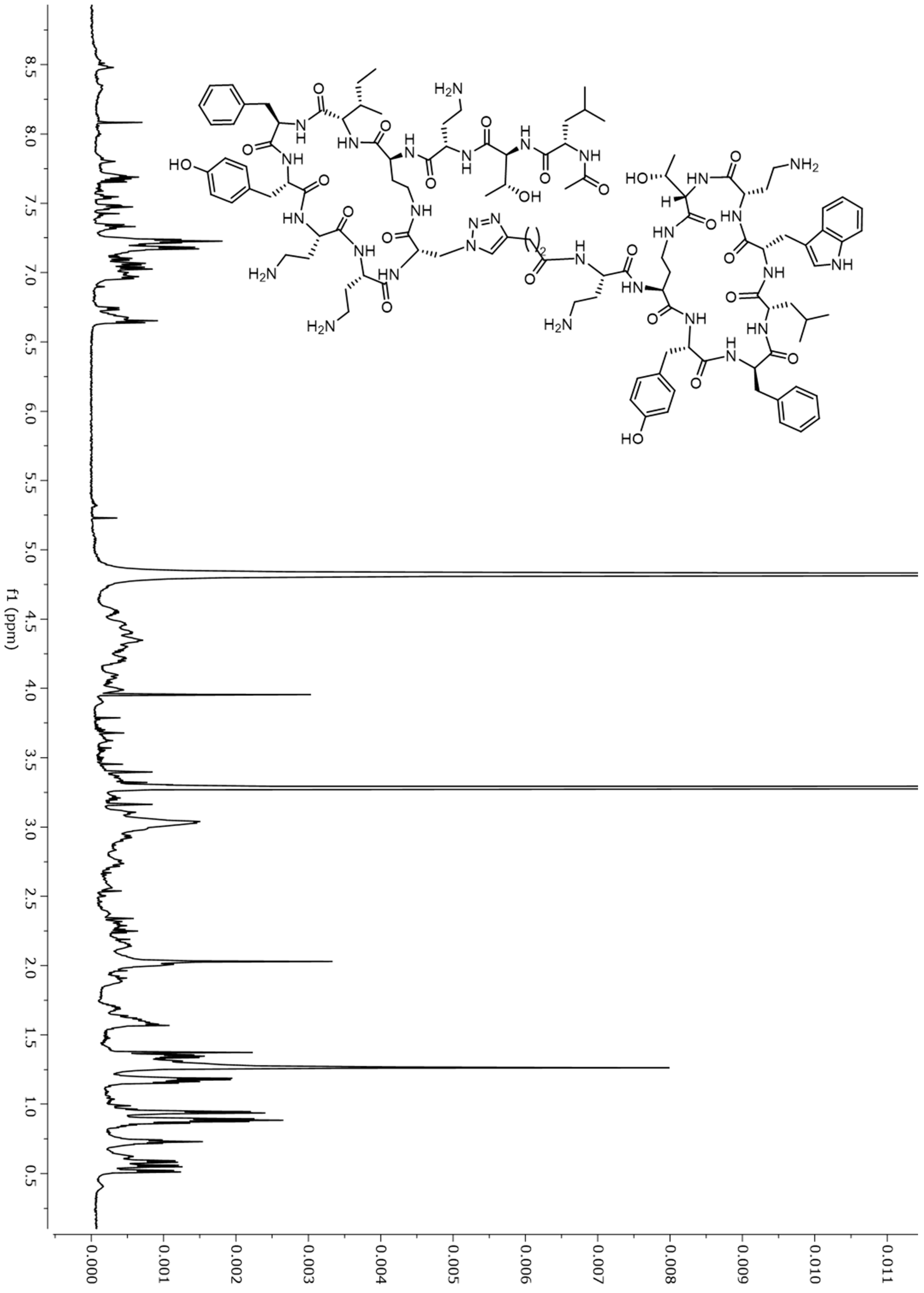

Dimer 7, ^{1}H NMR, 600 MHz, CD_3OD

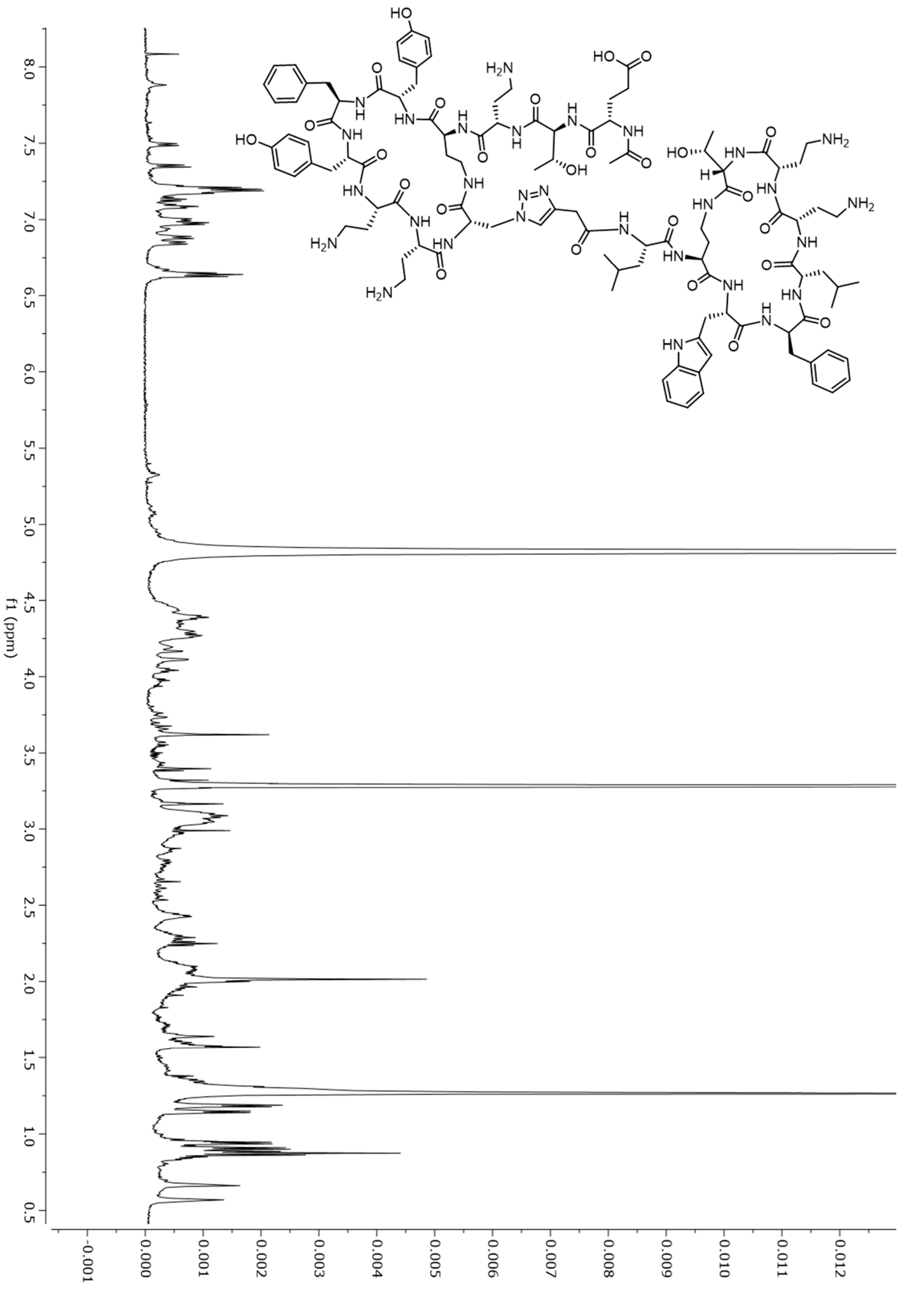

Dimer 10, ^1H NMR, 400 MHz, CD_3OD

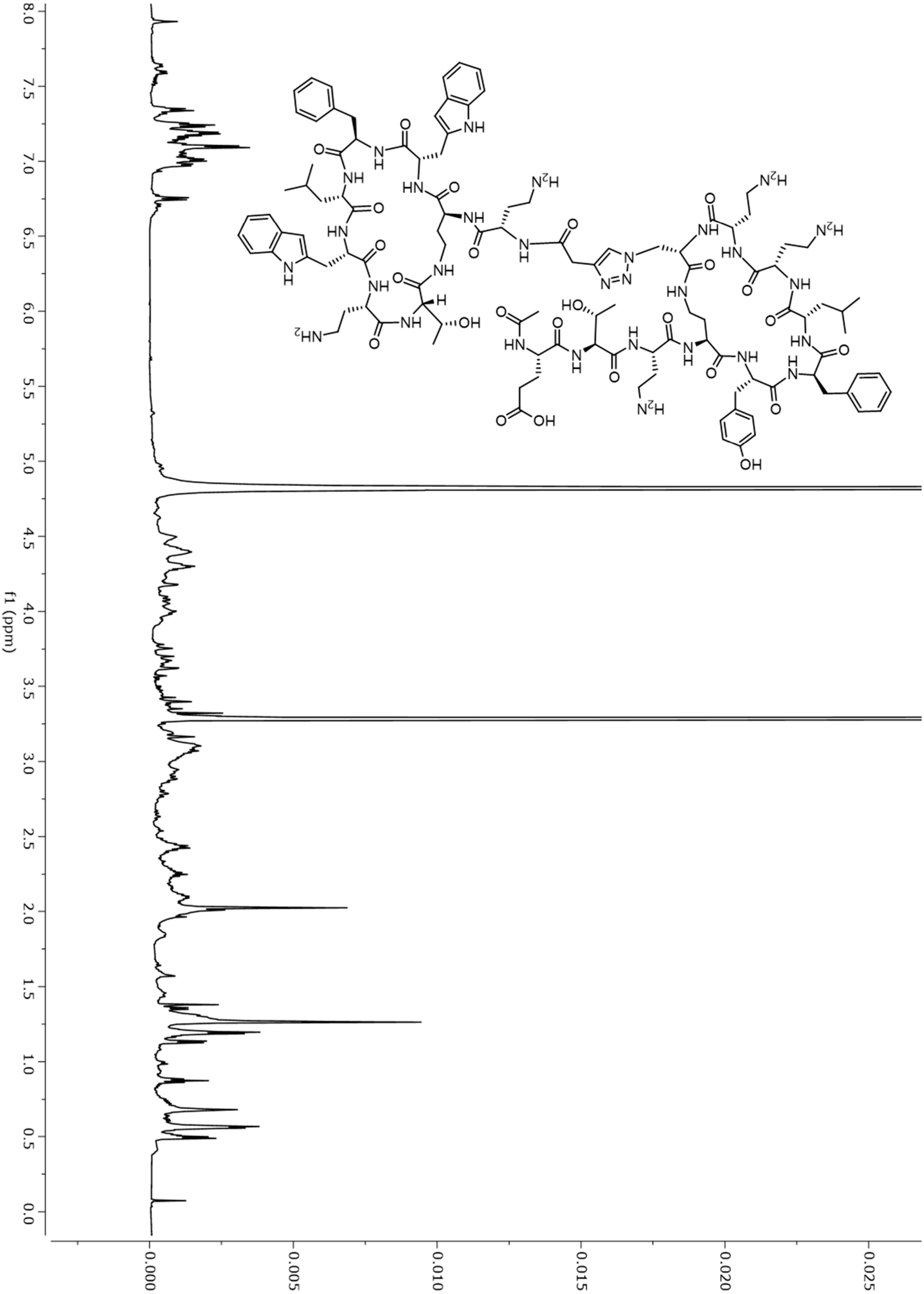

Dimer 14, ^{1}H NMR, 400 MHz, CD_3OD

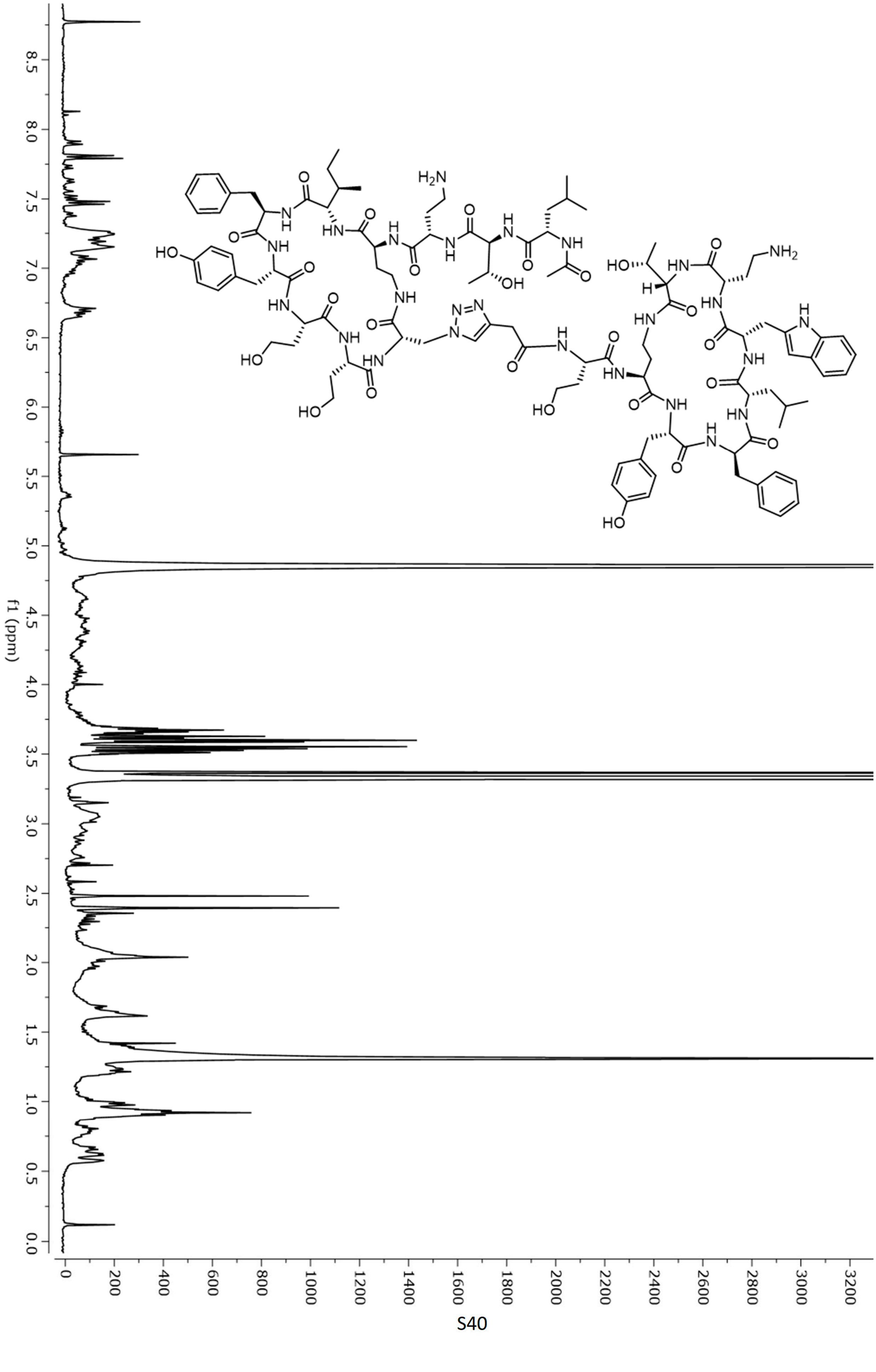

Dimer 16, ^{1}H NMR, 400 MHz, CD_3OD

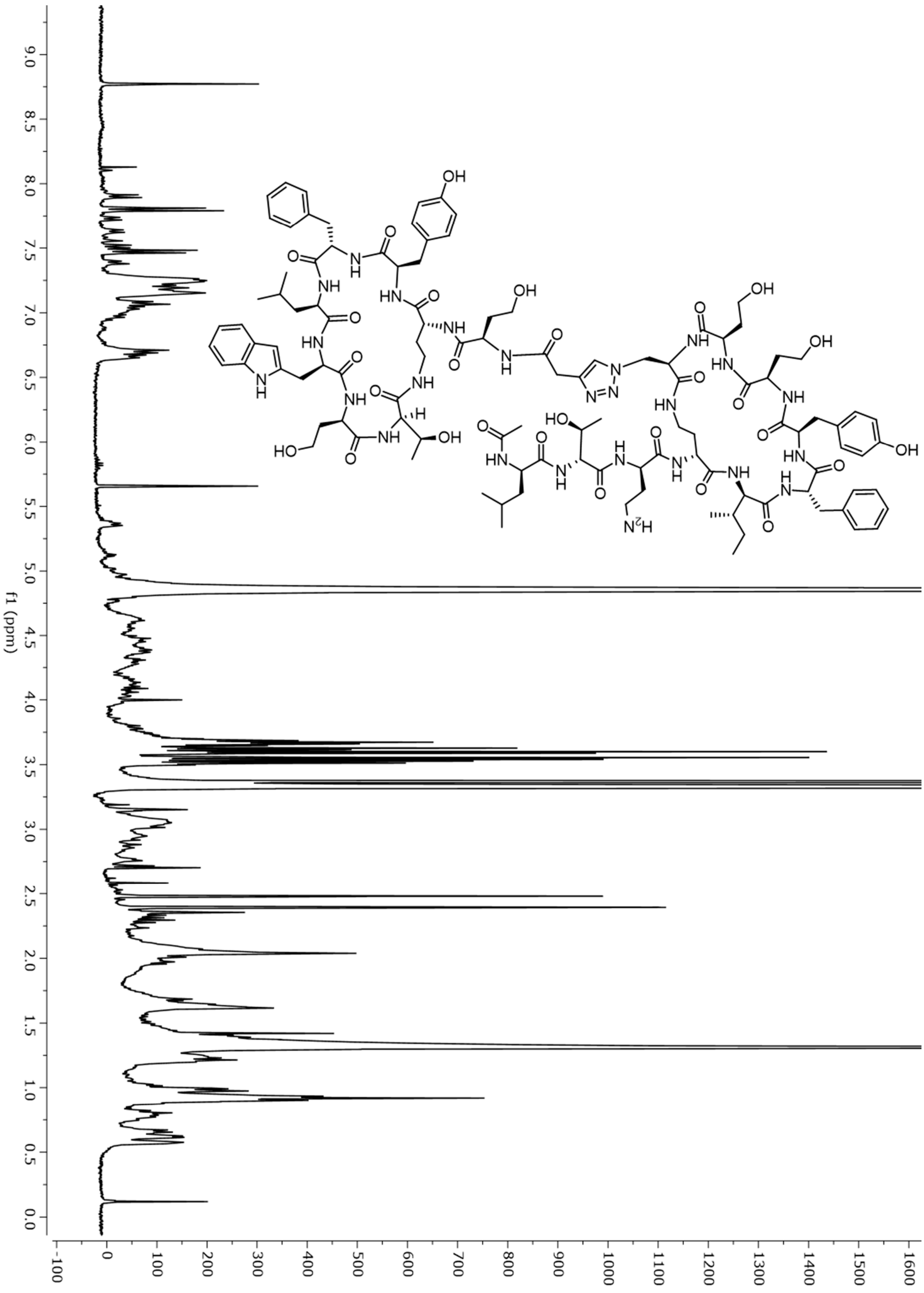

6. High resolution mass spectra

Monomer 1

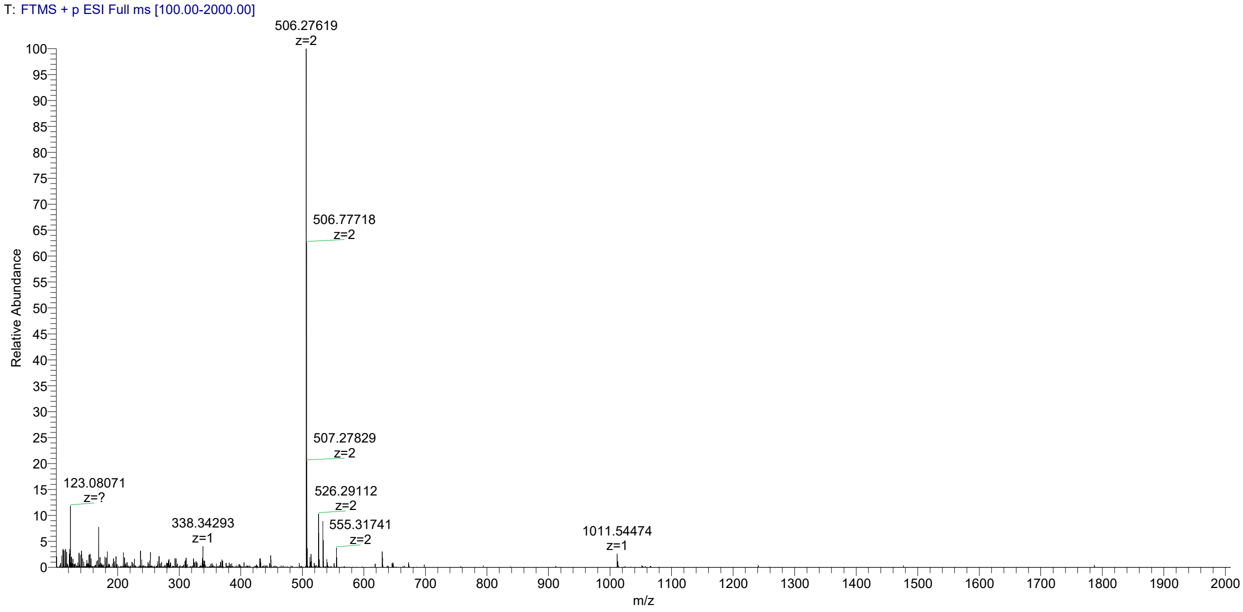

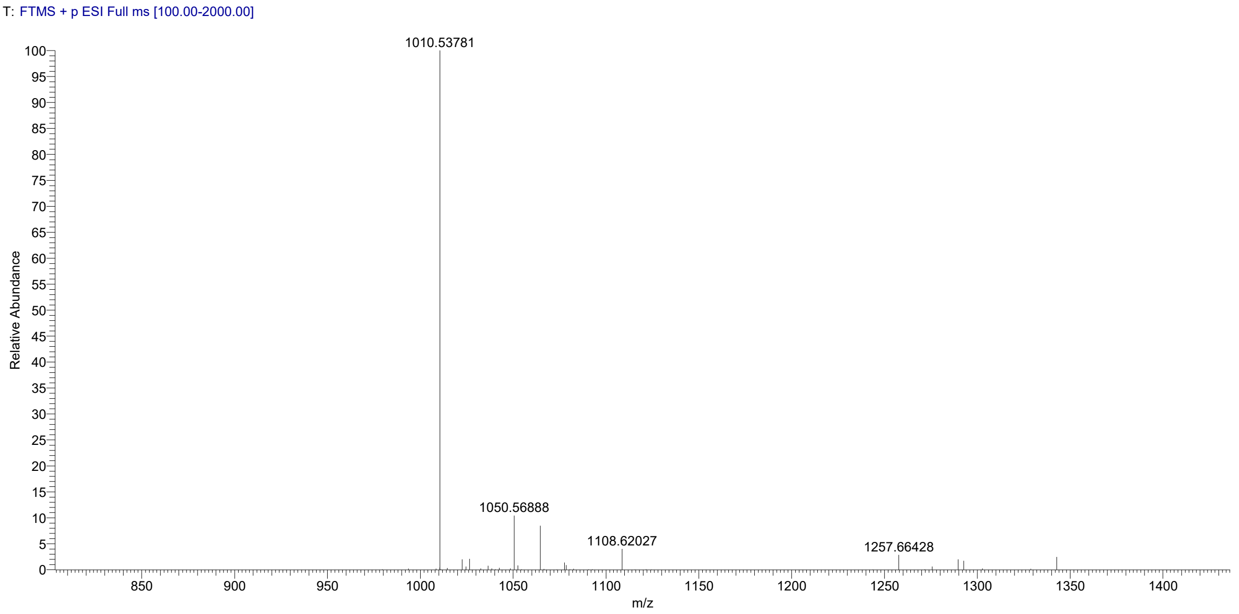

Monomer 5

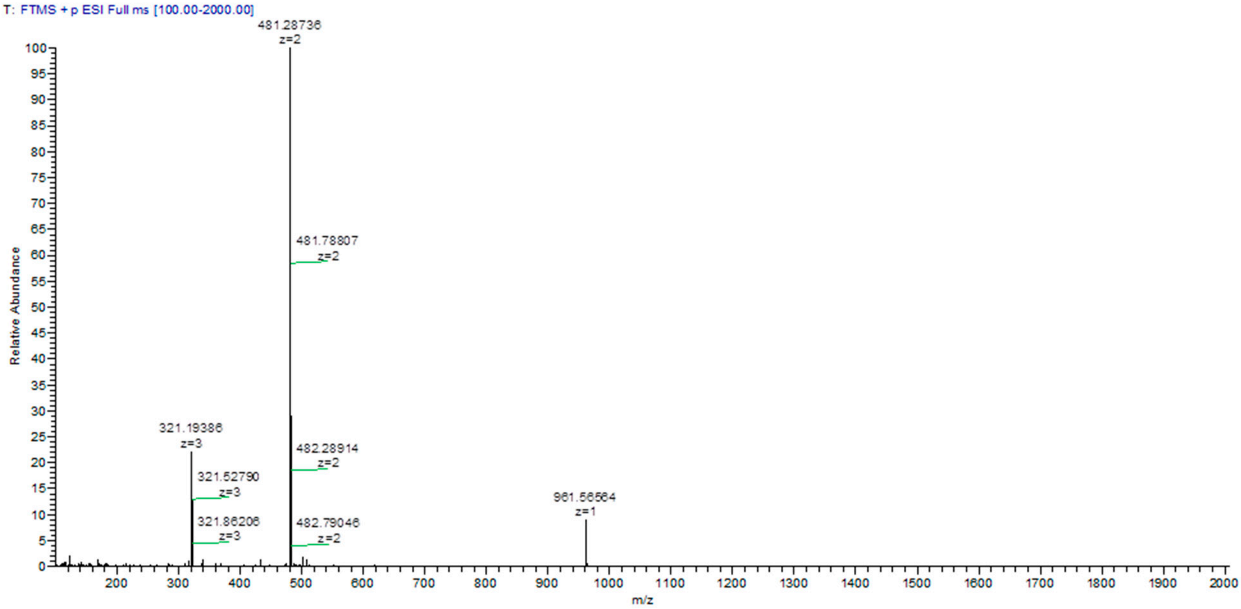
T: FTMS + p ESI Full ms [100.00-2000.00]
481.28736
z=2
481.78807
z=2
482.28914
z=2
482.79046
z=2
321.19386
z=3
321.52790
z=3
321.86206
z=3
961.56564
z=1
Relative Abundance
m/z

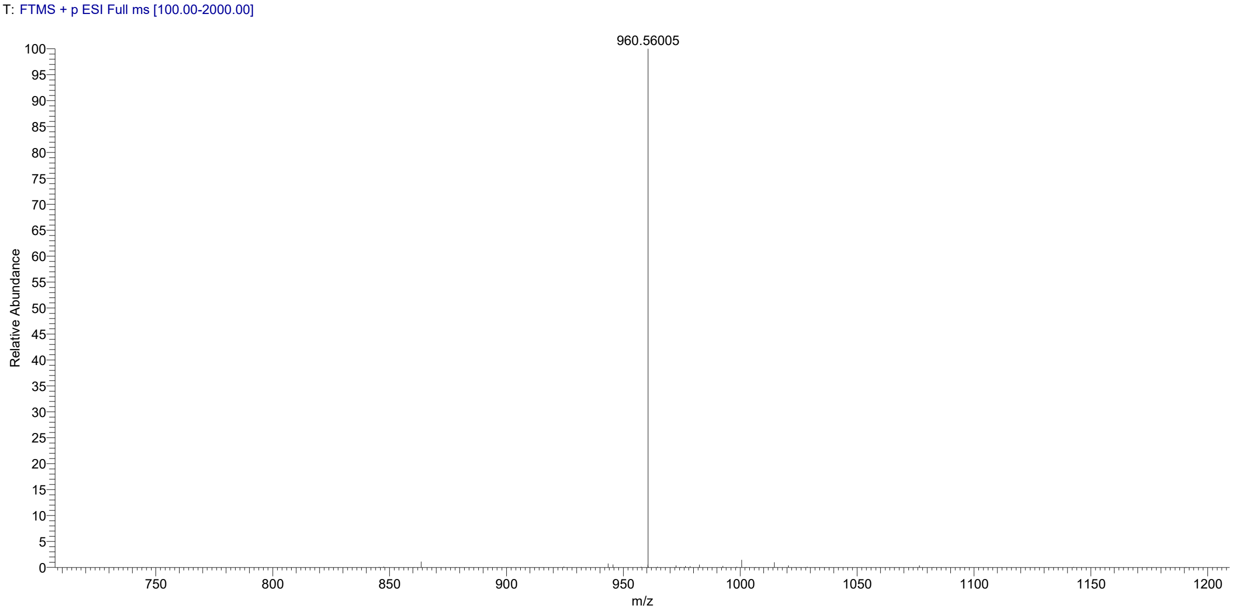
T: FTMS + p ESI Full ms [100.00-2000.00]
960.56005
Relative Abundance
m/z

Monomer 8

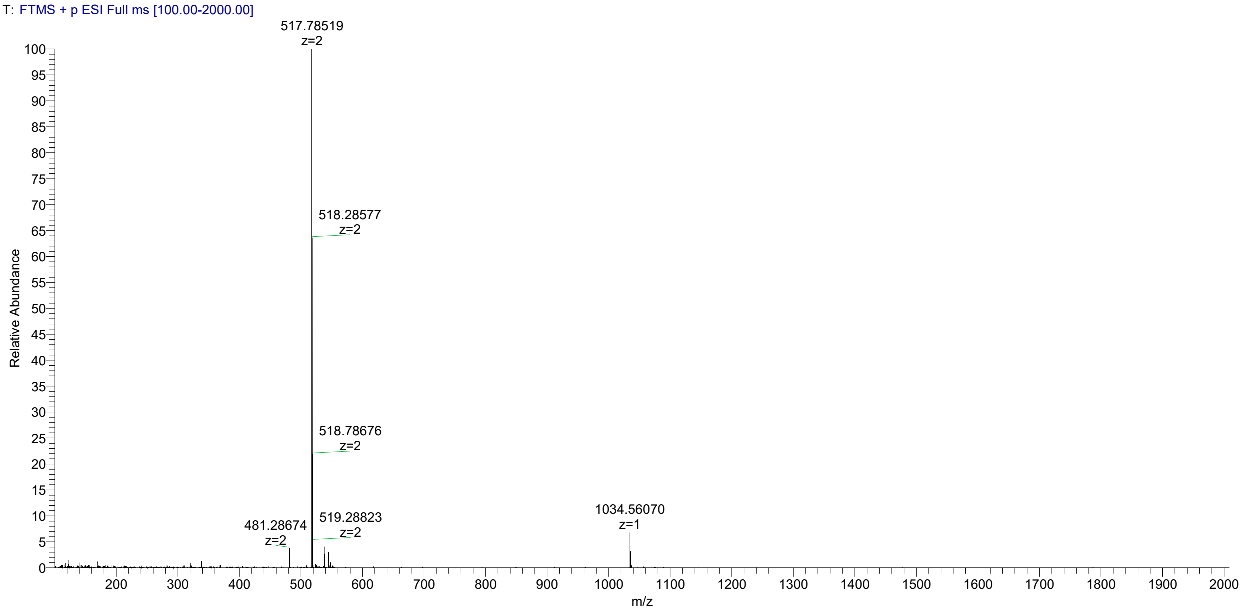
T: FTMS + p ESI Full ms [100.00-2000.00]
517.78519 z=2
518.28577 z=2
518.78676 z=2
519.28823 z=2
481.28674 z=2
1034.56070 z=1
Relative Abundance
m/z

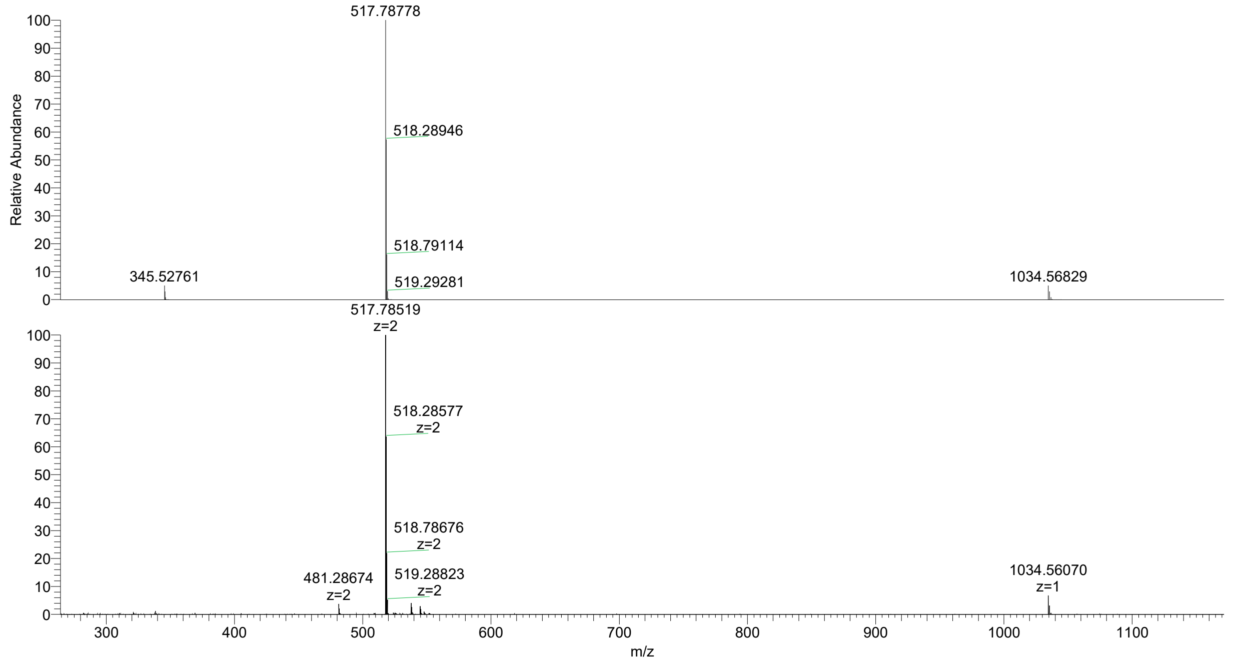
517.78778
518.28946
518.79114
519.29281
345.52761
1034.56829
517.78519 z=2
518.28577 z=2
518.78676 z=2
519.28823 z=2
481.28674 z=2
1034.56070 z=1
Relative Abundance
m/z

Monomer 12

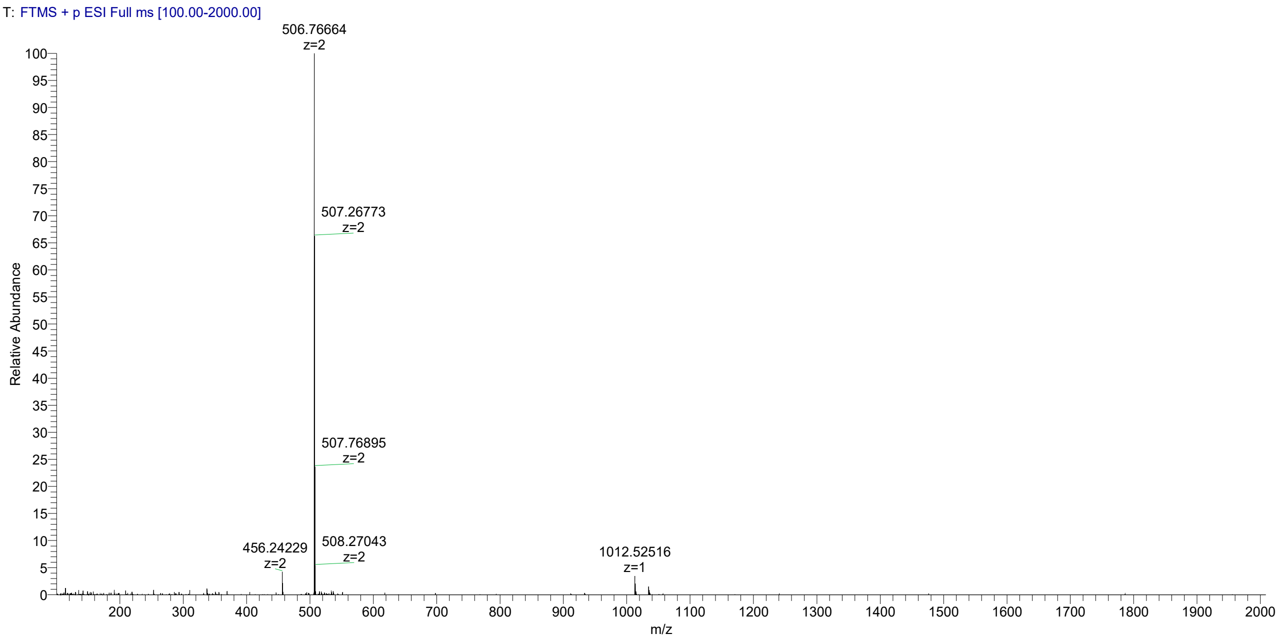

T: FTMS + p ESI Full ms [100.00-2000.00]
506.76664
z=2
507.26773
z=2
507.76895
z=2
508.27043
z=2
456.24229
z=2
1012.52516
z=1
Relative Abundance
m/z

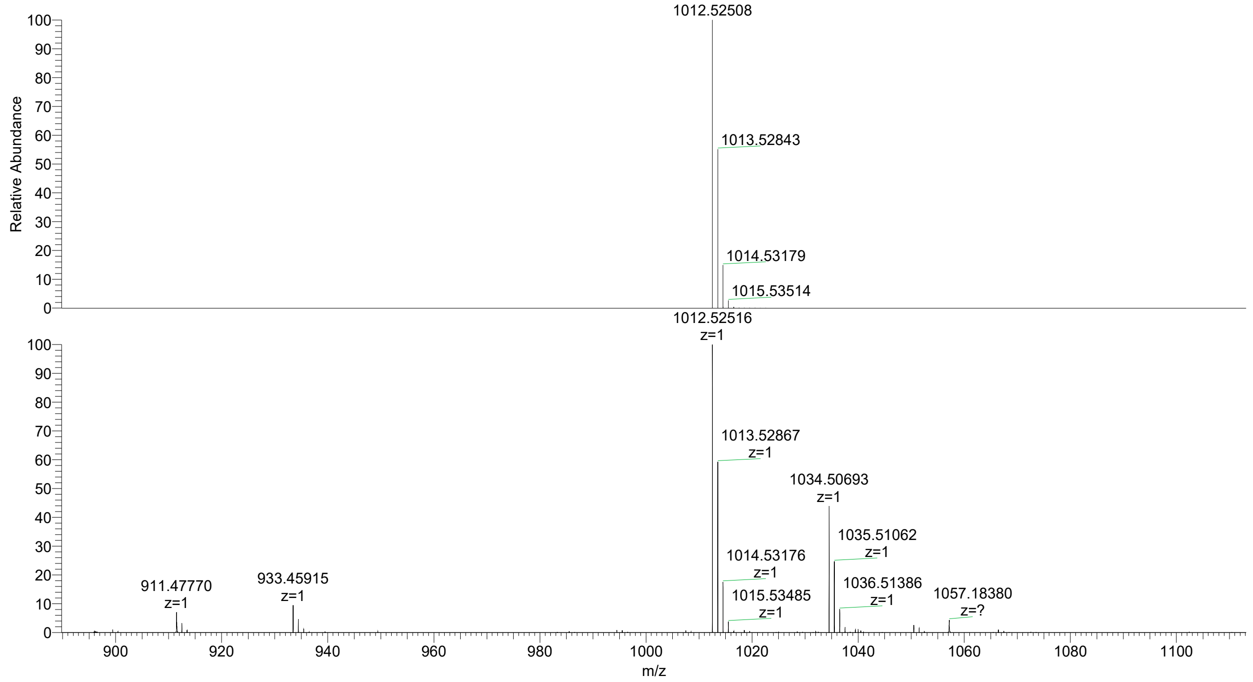

1012.52508
1013.52843
1014.53179
1015.53514
1012.52516
z=1
1013.52867
z=1
1034.50693
z=1
1035.51062
z=1
1014.53176
z=1
1036.51386
z=1
1015.53485
z=1
1057.18380
z=?
911.47770
z=1
933.45915
z=1
Relative Abundance
m/z

Monomer 15

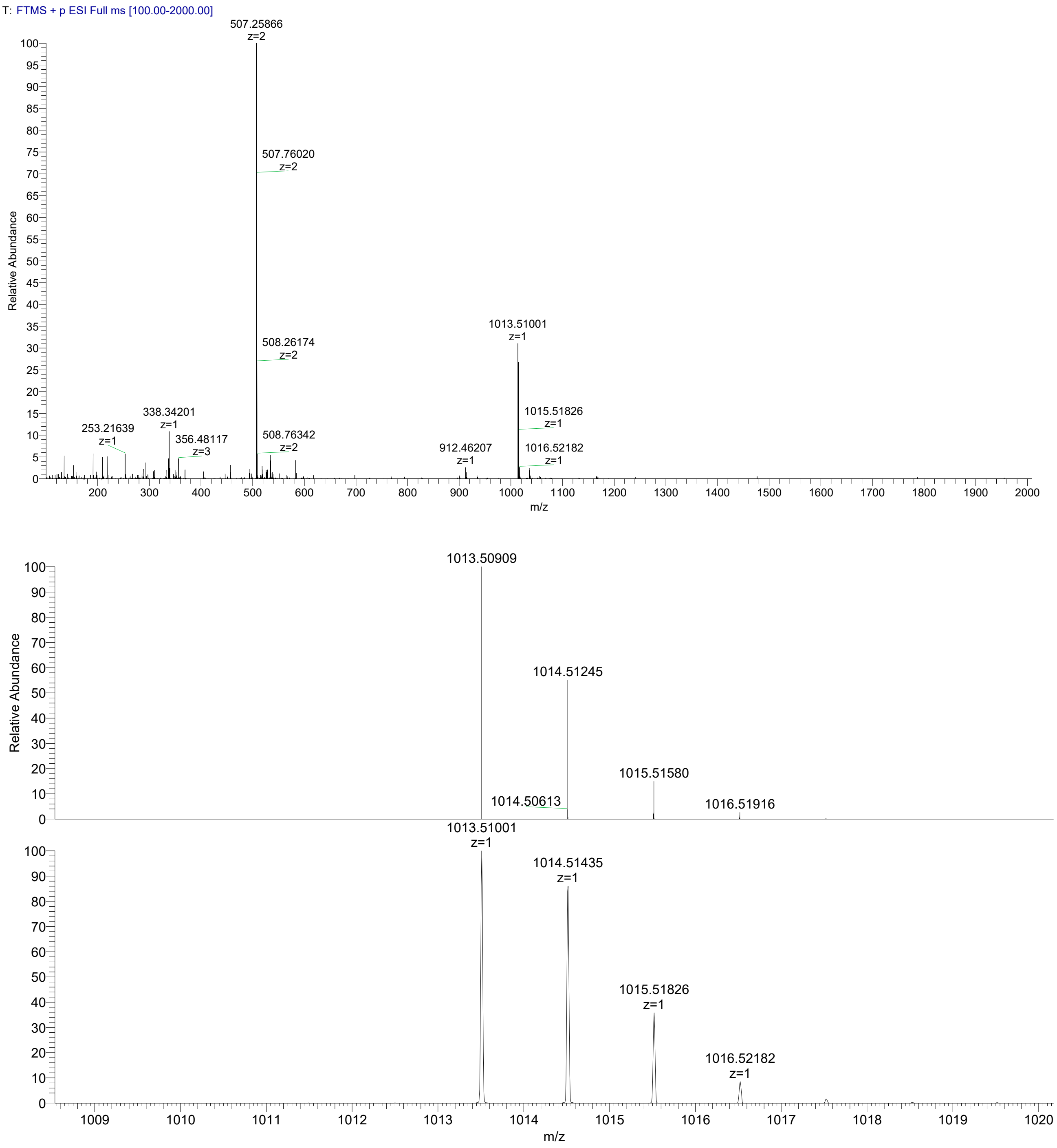

T: FTMS + p ESI Full ms [100.00-2000.00]
507.25866 z=2
507.76020 z=2
508.26174 z=2
508.76342 z=2
253.21639 z=1
338.34201 z=1
356.48117 z=3
912.46207 z=1
1013.51001 z=1
1015.51826 z=1
1016.52182 z=1
Relative Abundance
m/z
1013.50909
1014.51245
1014.50613
1015.51580
1016.51916
1013.51001 z=1
1014.51435 z=1
1015.51826 z=1
1016.52182 z=1

Monomer 2

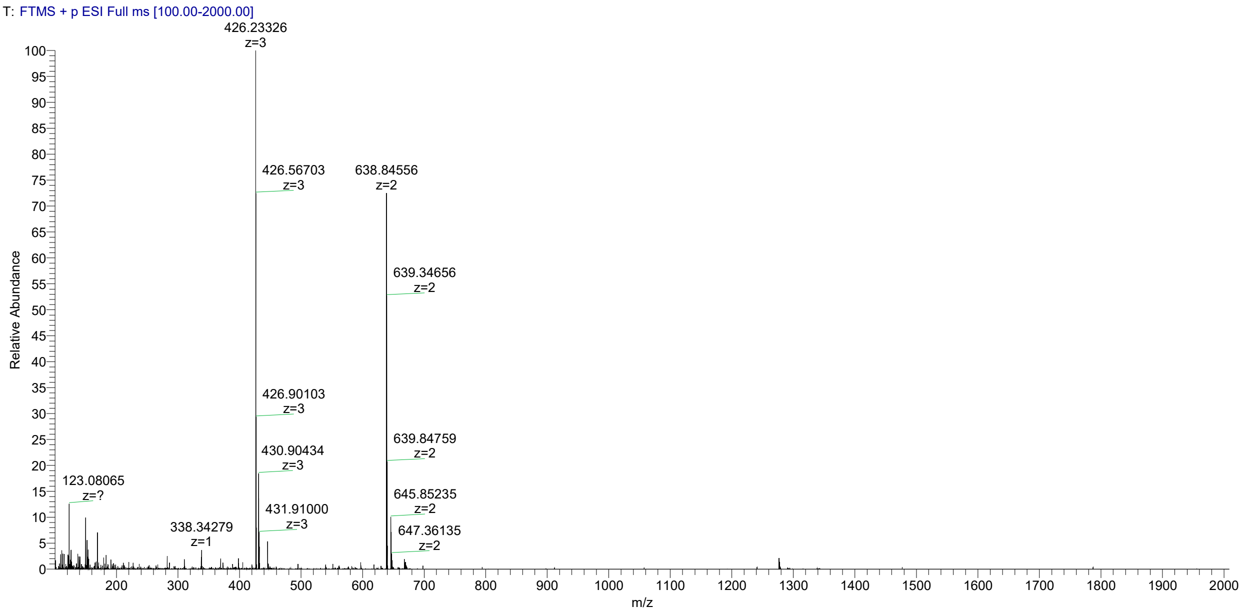
T: FTMS + p ESI Full ms [100.00-2000.00]
426.23326
z=3
426.56703
z=3
638.84556
z=2
639.34656
z=2
426.90103
z=3
639.84759
z=2
430.90434
z=3
123.08065
z=?
645.85235
z=2
431.91000
z=3
338.34279
z=1
647.36135
z=2
Relative Abundance
m/z

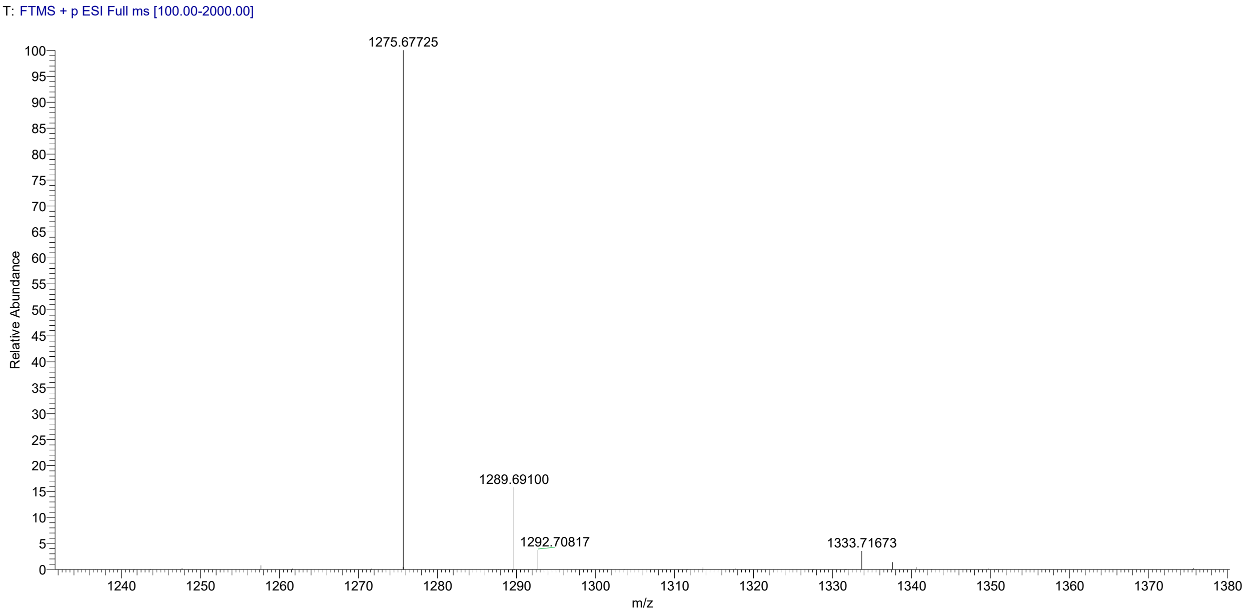
T: FTMS + p ESI Full ms [100.00-2000.00]
1275.67725
1289.69100
1292.70817
1333.71673
Relative Abundance
m/z

Monomer 6

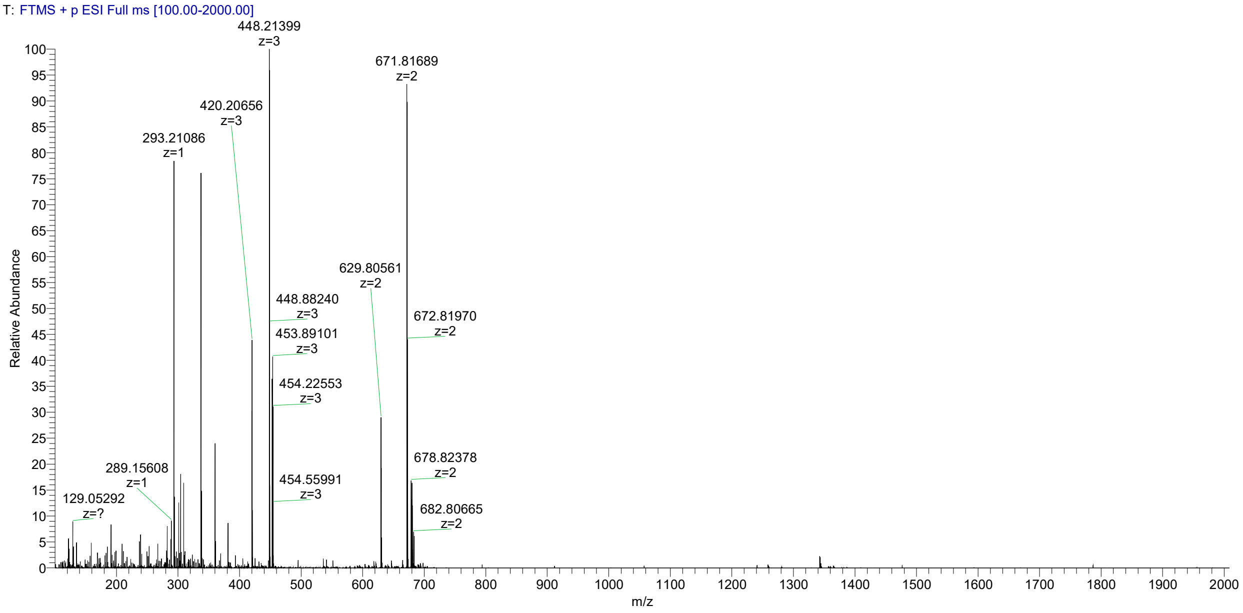
T: FTMS + p ESI Full ms [100.00-2000.00]
448.21399 z=3
671.81689 z=2
420.20656 z=3
293.21086 z=1
629.80561 z=2
448.88240 z=3
672.81970 z=2
453.89101 z=3
454.22553 z=3
678.82378 z=2
289.15608 z=1
454.55991 z=3
129.05292 z=?
682.80665 z=2
Relative Abundance
m/z

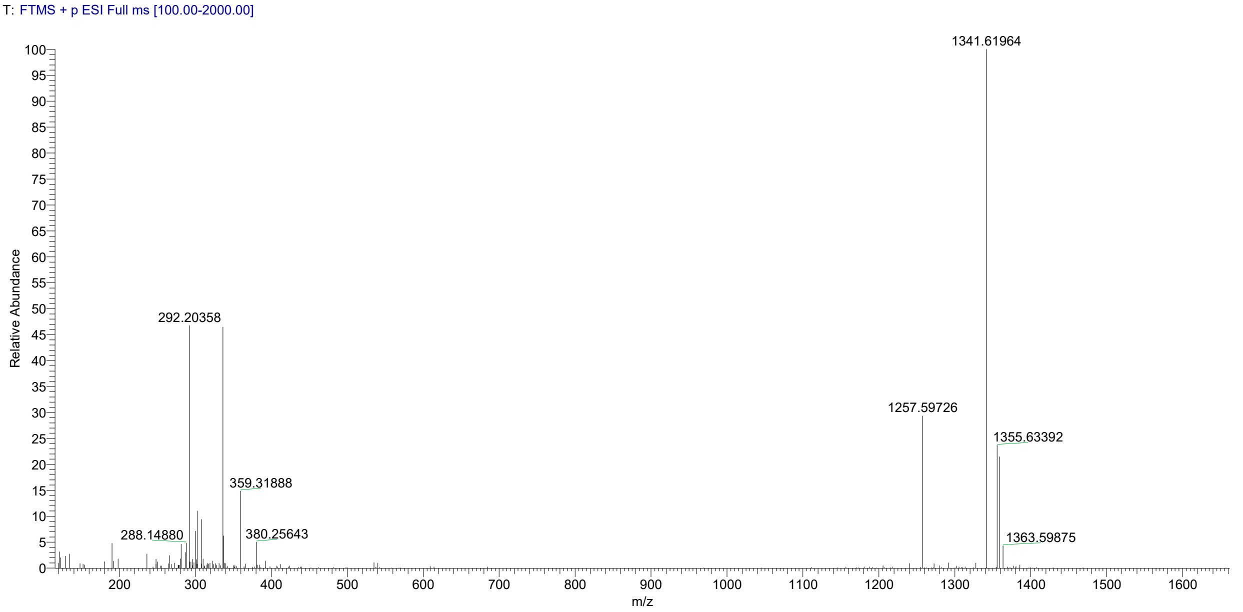
T: FTMS + p ESI Full ms [100.00-2000.00]
1341.61964
292.20358
1257.59726
1355.63392
359.31888
288.14880
380.25643
1363.59875
Relative Abundance
m/z

Monomer 9

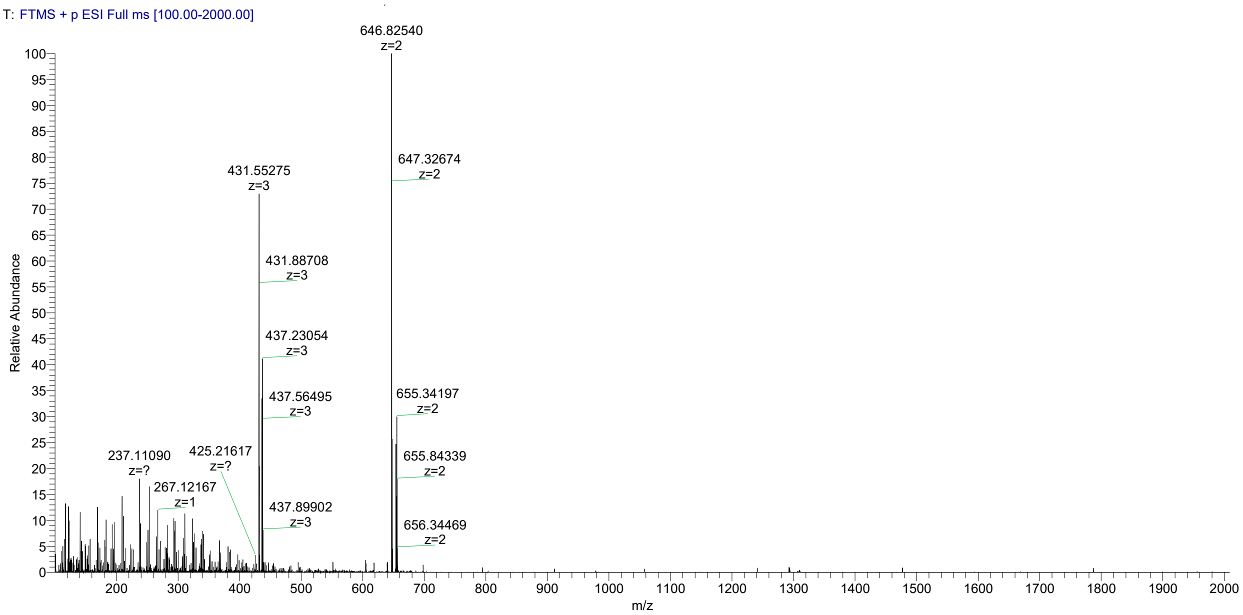
T: FTMS + p ESI Full ms [100.00-2000.00]
646.82540 z=2
647.32674 z=2
431.55275 z=3
431.88708 z=3
437.23054 z=3
437.56495 z=3
655.34197 z=2
655.84339 z=2
656.34469 z=2
237.11090 z=?
425.21617 z=?
267.12167 z=1
437.89902 z=3
Relative Abundance
m/z

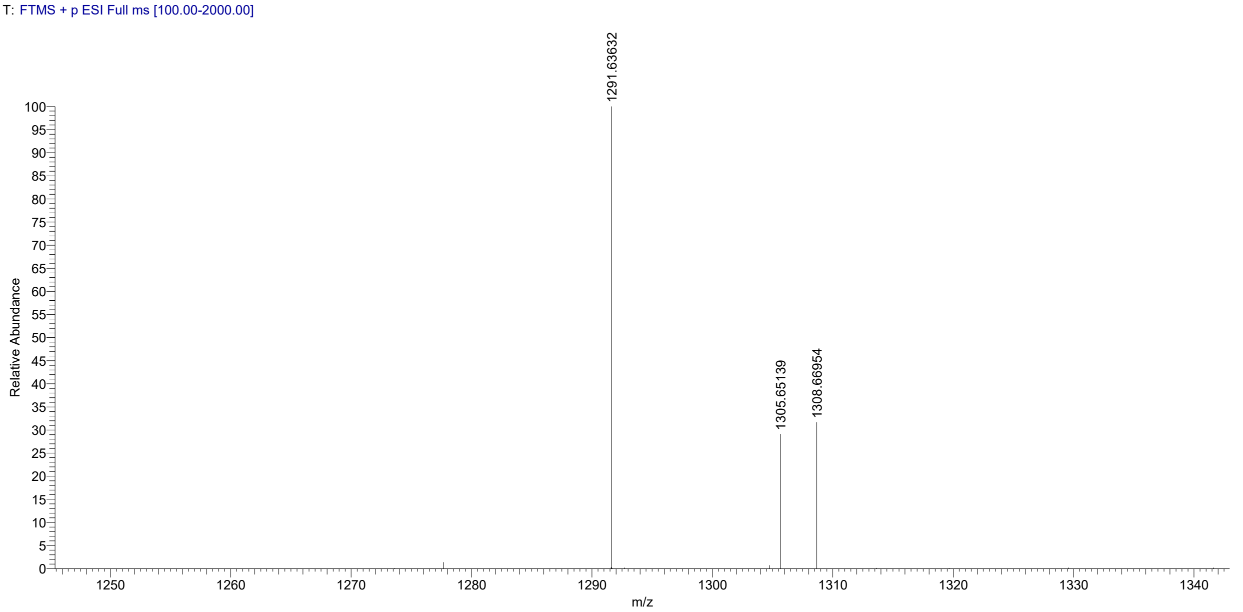
T: FTMS + p ESI Full ms [100.00-2000.00]
1291.63632
1305.65139
1308.66954
Relative Abundance
m/z

Monomer 13

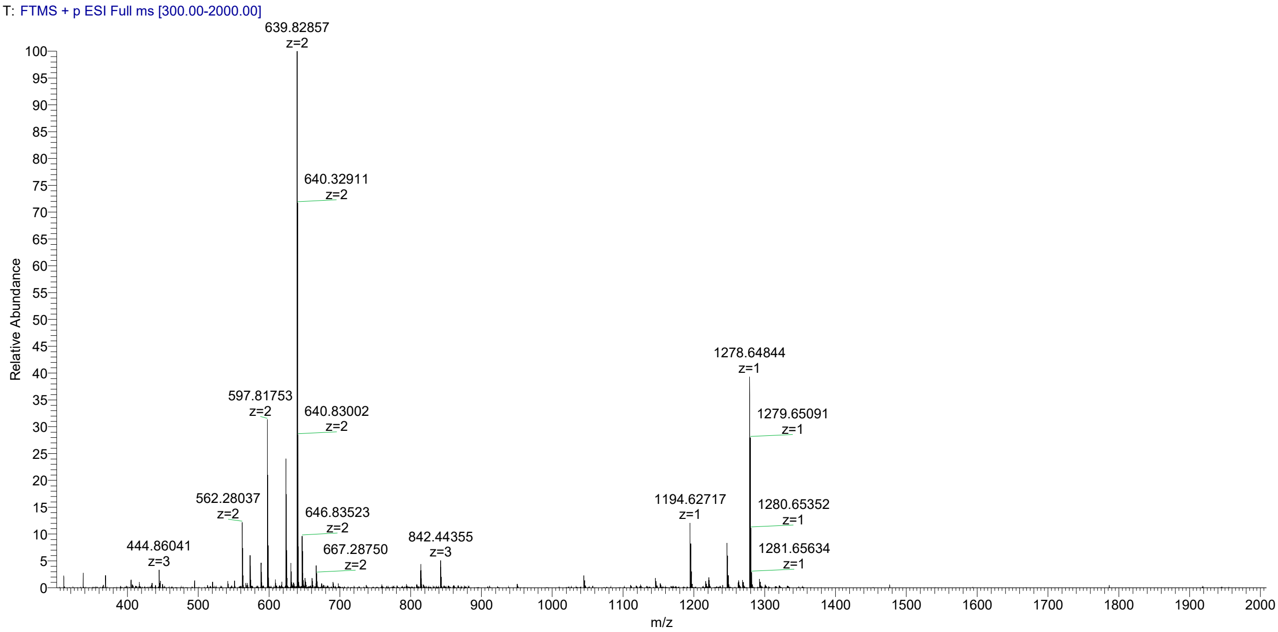
T: FTMS + p ESI Full ms [300.00-2000.00]
639.82857 z=2
640.32911 z=2
1278.64844 z=1
597.81753 z=2
640.83002 z=2
1279.65091 z=1
562.28037 z=2
646.83523 z=2
1194.62717 z=1
1280.65352 z=1
444.86041 z=3
842.44355 z=3
667.28750 z=2
1281.65634 z=1
Relative Abundance
m/z

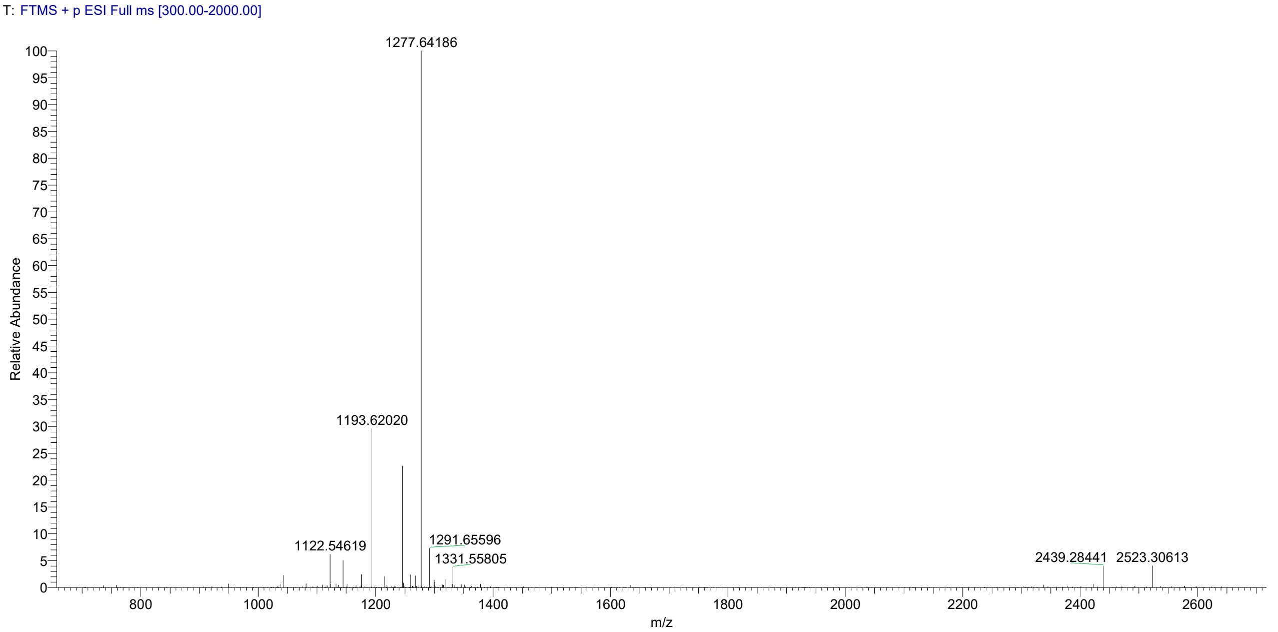
T: FTMS + p ESI Full ms [300.00-2000.00]
1277.64186
1193.62020
1122.54619
1291.65596
1331.55805
2439.28441
2523.30613
Relative Abundance
m/z

Dimer 3

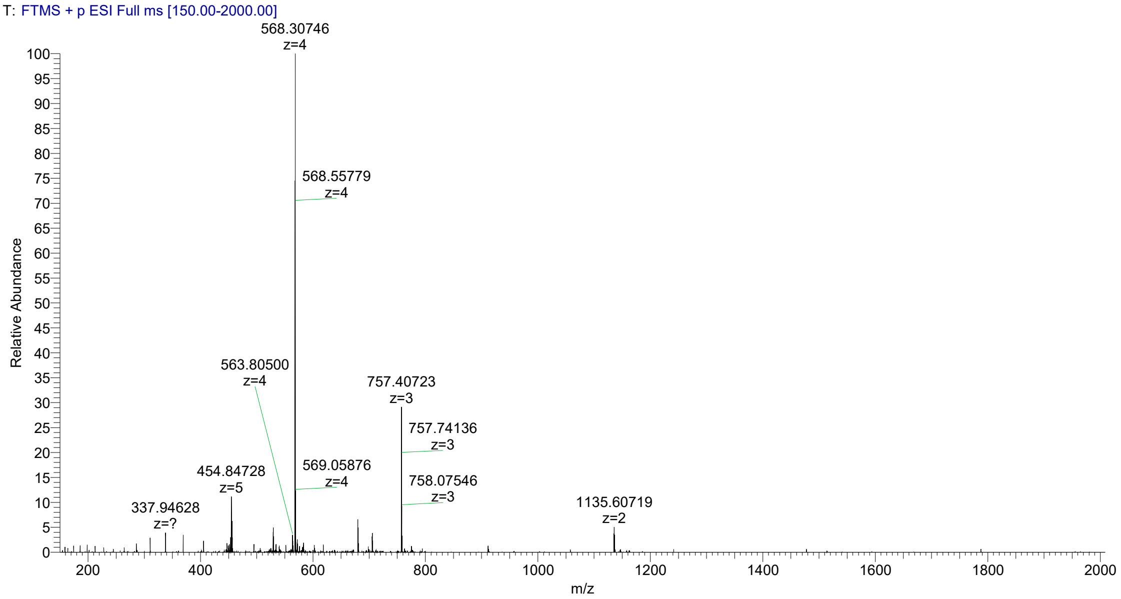
T: FTMS + p ESI Full ms [150.00-2000.00]
568.30746 z=4
568.55779 z=4
563.80500 z=4
757.40723 z=3
757.74136 z=3
569.05876 z=4
758.07546 z=3
454.84728 z=5
337.94628 z=?
1135.60719 z=2
Relative Abundance
m/z

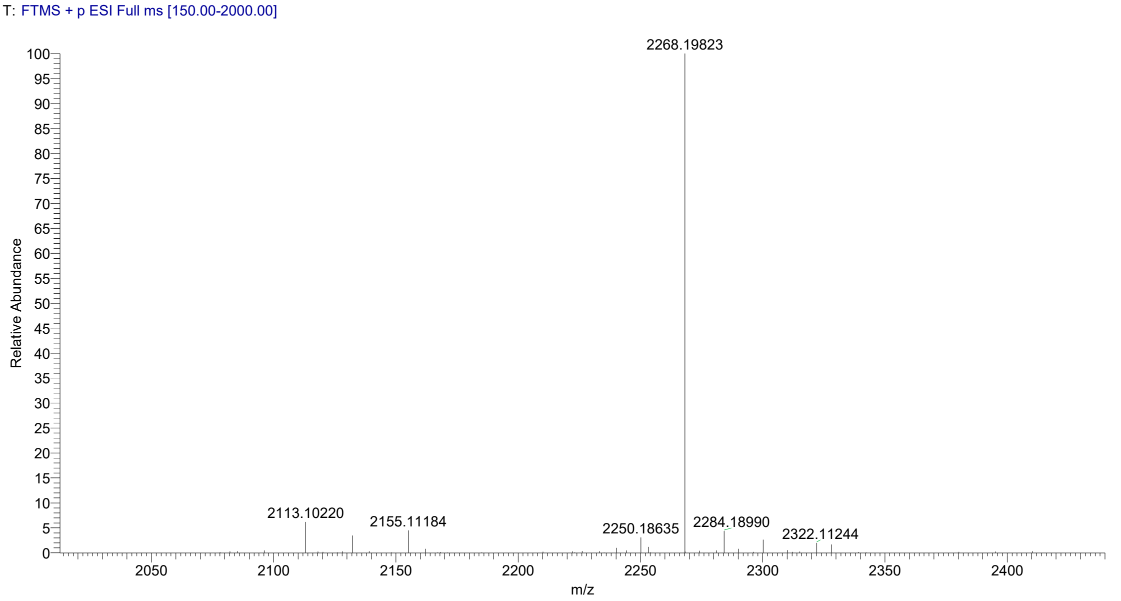
T: FTMS + p ESI Full ms [150.00-2000.00]
2268.19823
2113.10220
2155.11184
2250.18635
2284.18990
2322.11244
Relative Abundance
m/z

Dimer 4

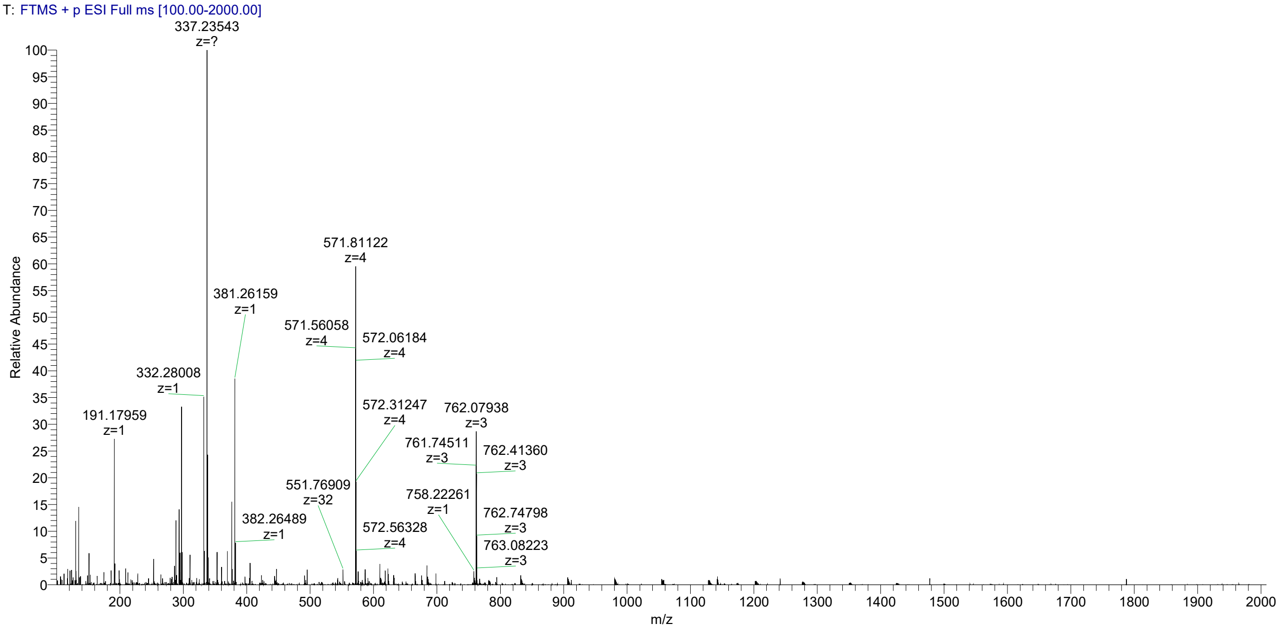
T: FTMS + p ESI Full ms [100.00-2000.00]
337.23543
z=?
571.81122
z=4
381.26159
z=1
571.56058
z=4
572.06184
z=4
332.28008
z=1
572.31247
z=4
762.07938
z=3
191.17959
z=1
761.74511
z=3
762.41360
z=3
551.76909
z=32
758.22261
z=1
382.26489
z=1
762.74798
z=3
572.56328
z=4
763.08223
z=3
Relative Abundance
m/z

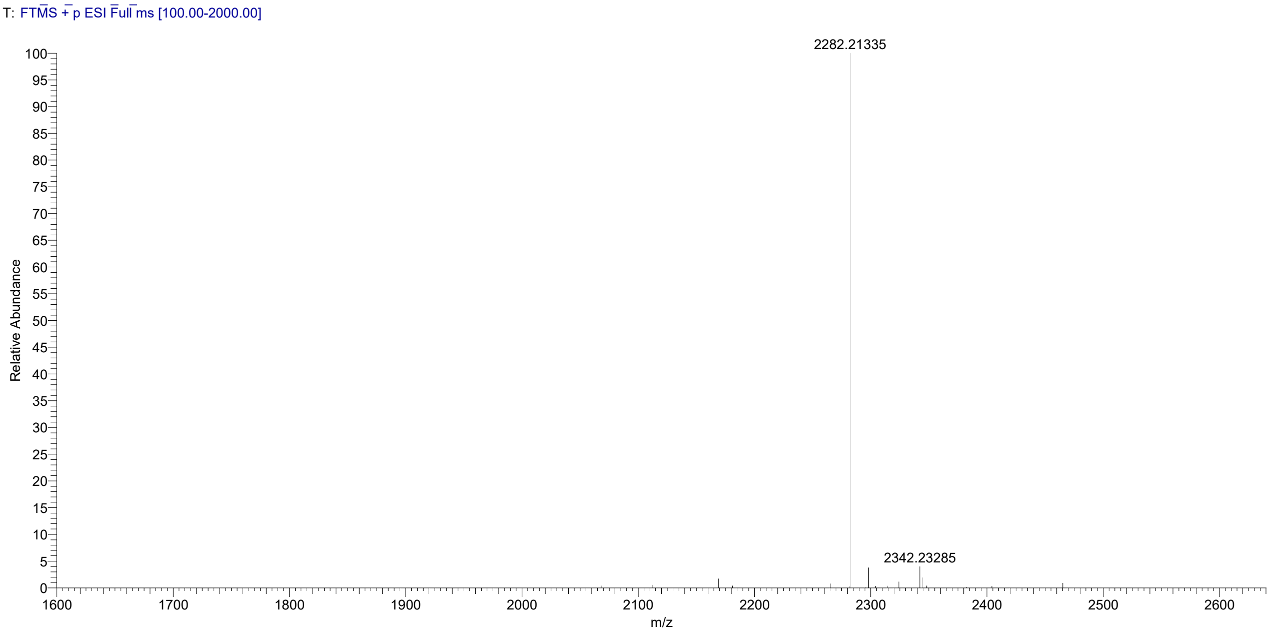
T: FTMS + p ESI Full ms [100.00-2000.00]
2282.21335
2342.23285
Relative Abundance
m/z

Dimer 7

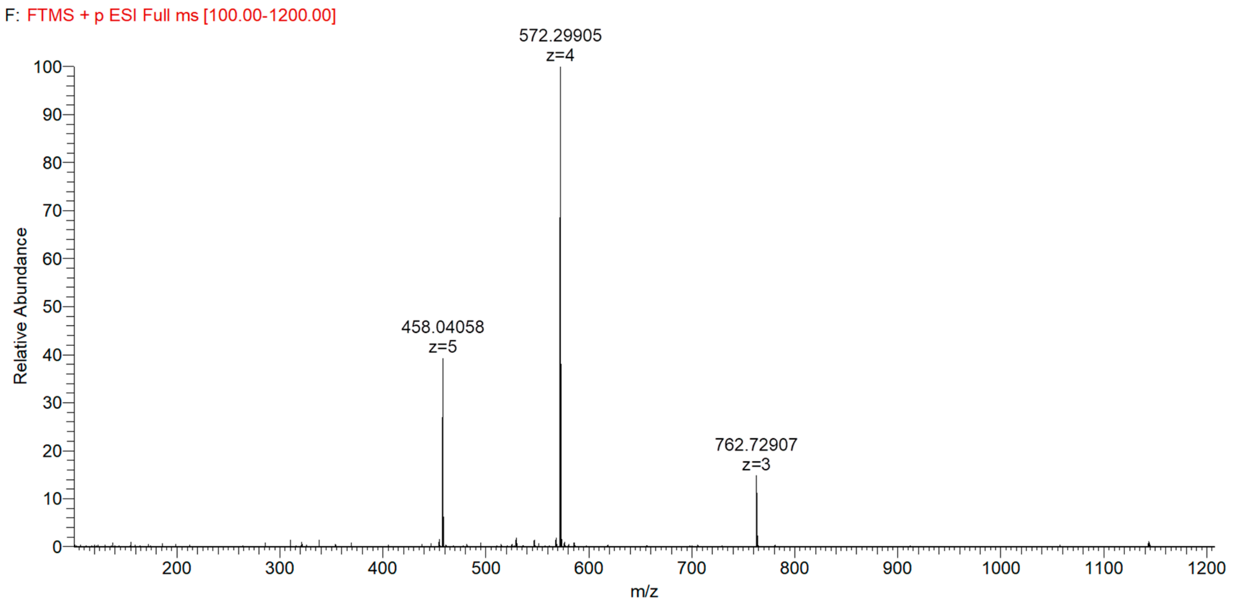

F: FTMS + p ESI Full ms [100.00-1200.00]
572.29905
z=4
458.04058
z=5
762.72907
z=3
Relative Abundance
m/z

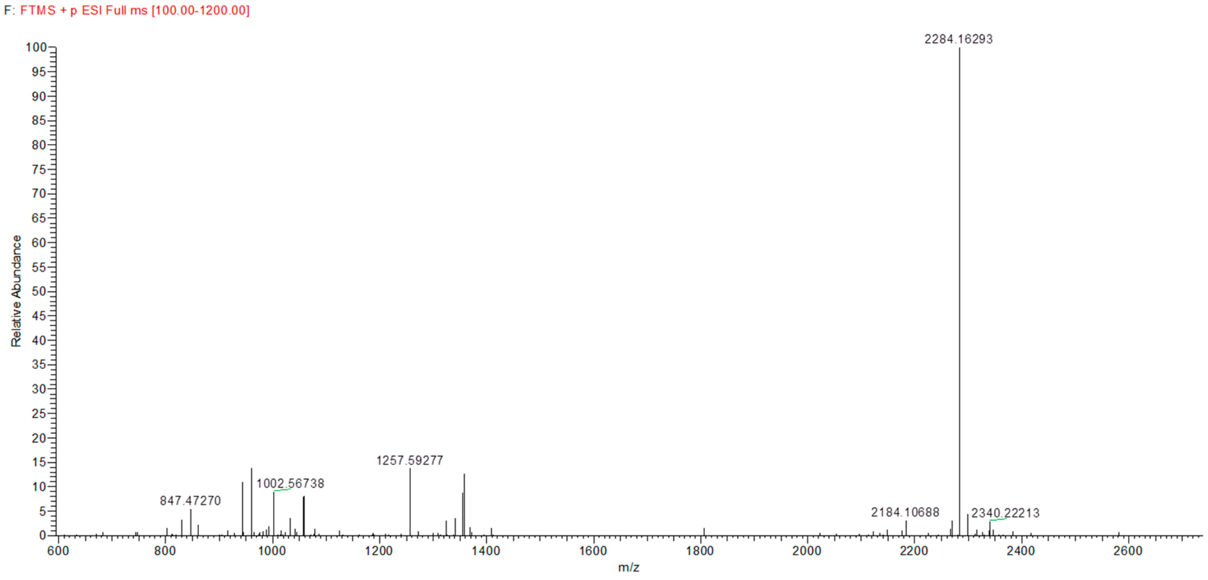

F: FTMS + p ESI Full ms [100.00-1200.00]
2284.16293
1257.59277
1002.56738
847.47270
2184.10688
2340.22213
Relative Abundance
m/z

Dimer 10

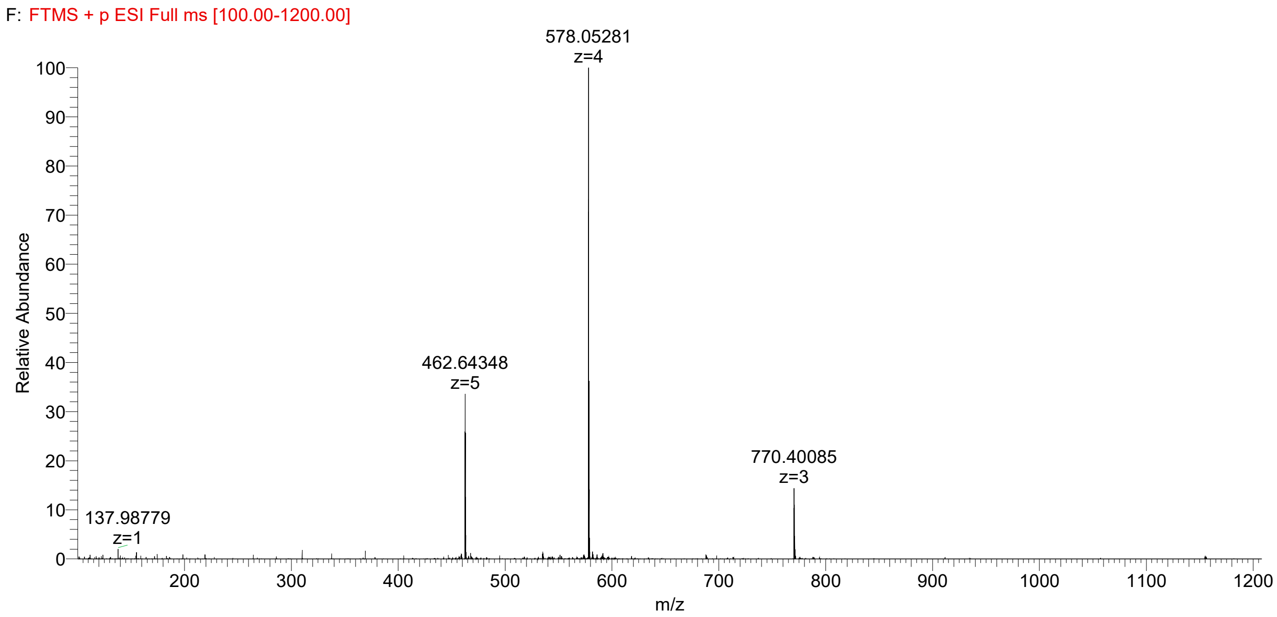

F: FTMS + p ESI Full ms [100.00-1200.00]
578.05281
z=4
462.64348
z=5
770.40085
z=3
137.98779
z=1
Relative Abundance
m/z

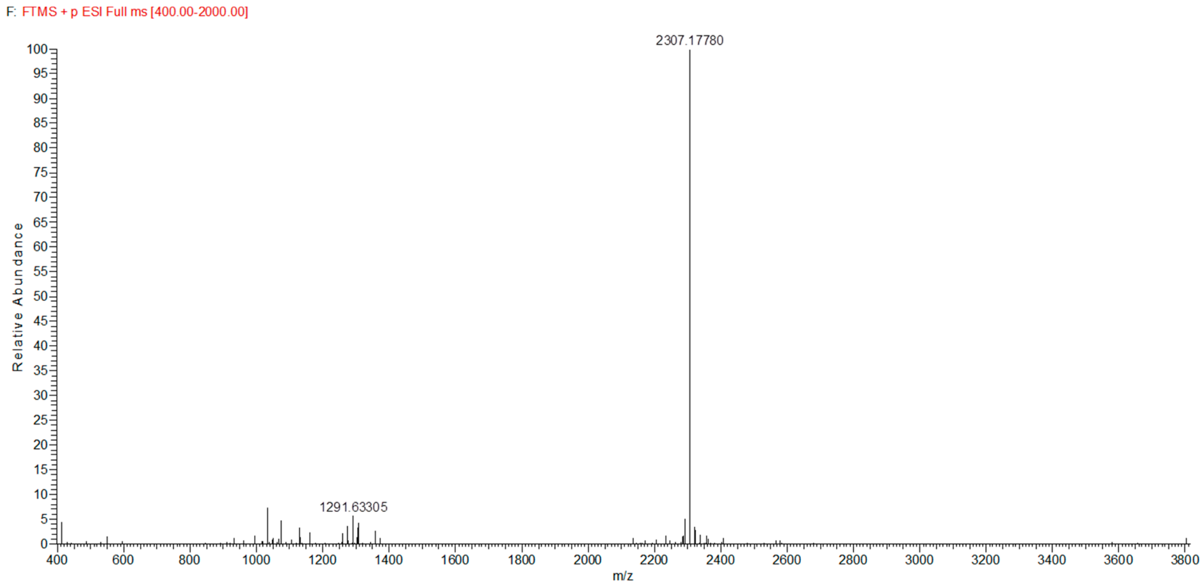

F: FTMS + p ESI Full ms [400.00-2000.00]
2307.17780
1291.63305
Relative Abundance
m/z

Dimer 14

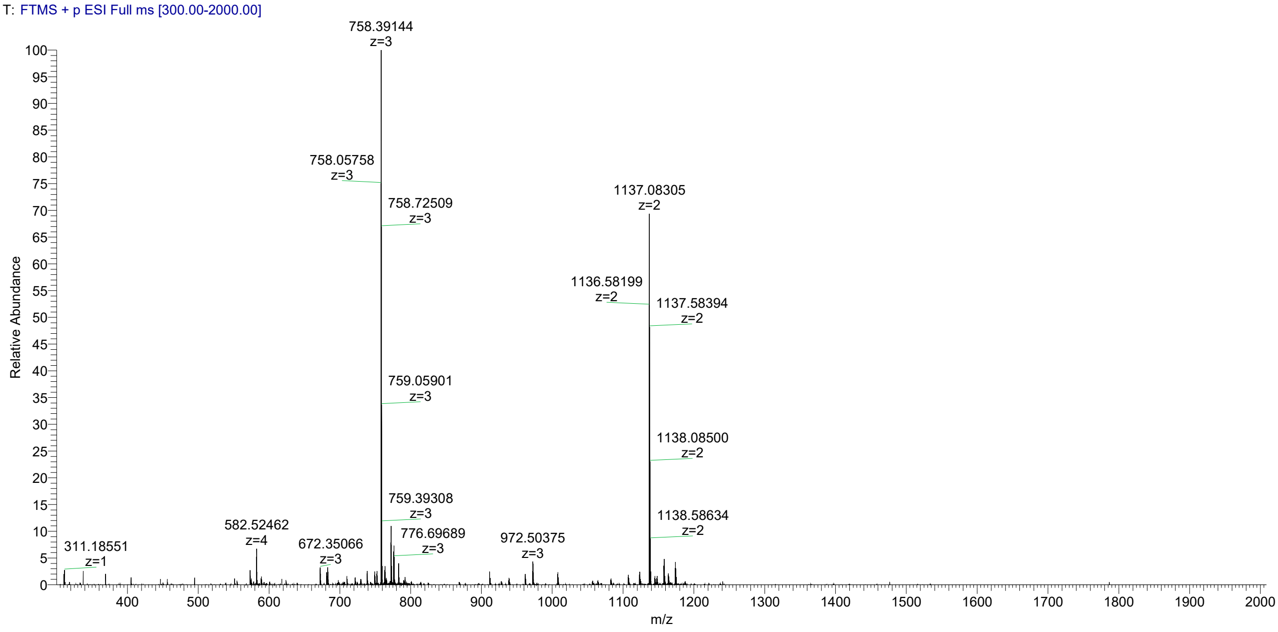
T: FTMS + p ESI Full ms [300.00-2000.00]
758.39144
z=3
758.05758
z=3
758.72509
z=3
759.05901
z=3
759.39308
z=3
776.69689
z=3
1137.08305
z=2
1136.58199
z=2
1137.58394
z=2
1138.08500
z=2
1138.58634
z=2
311.18551
z=1
582.52462
z=4
672.35066
z=3
972.50375
z=3
Relative Abundance
m/z

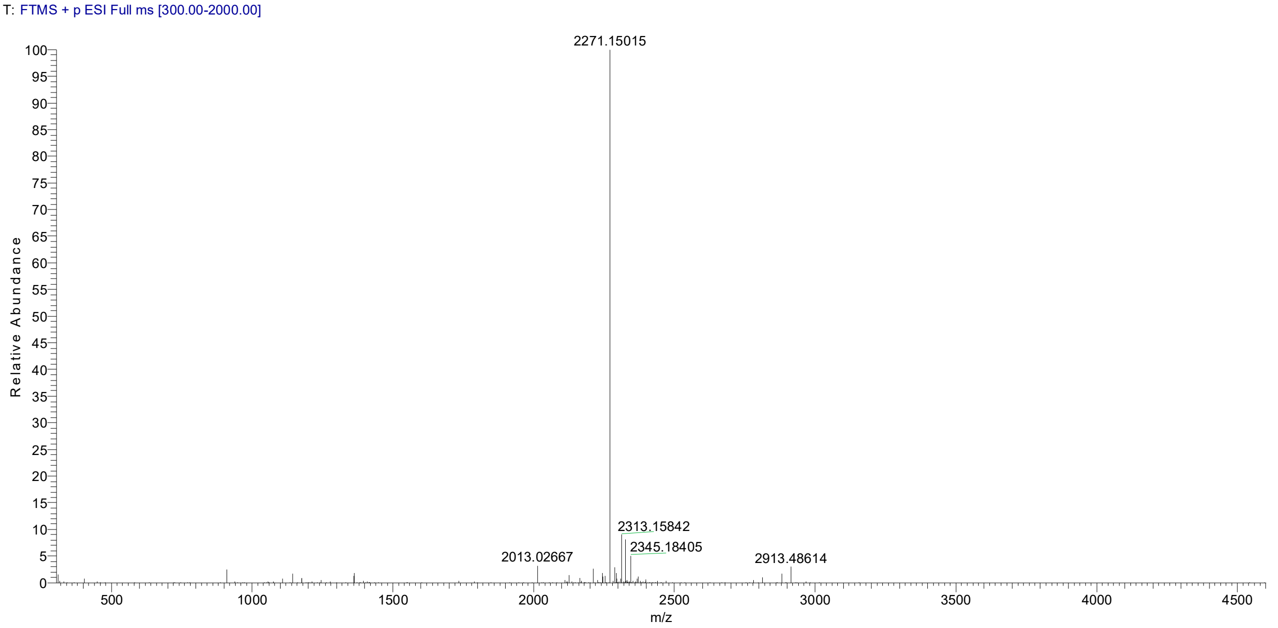
T: FTMS + p ESI Full ms [300.00-2000.00]
2271.15015
2313.15842
2345.18405
2013.02667
2913.48614
Relative Abundance
m/z

Dimer 16

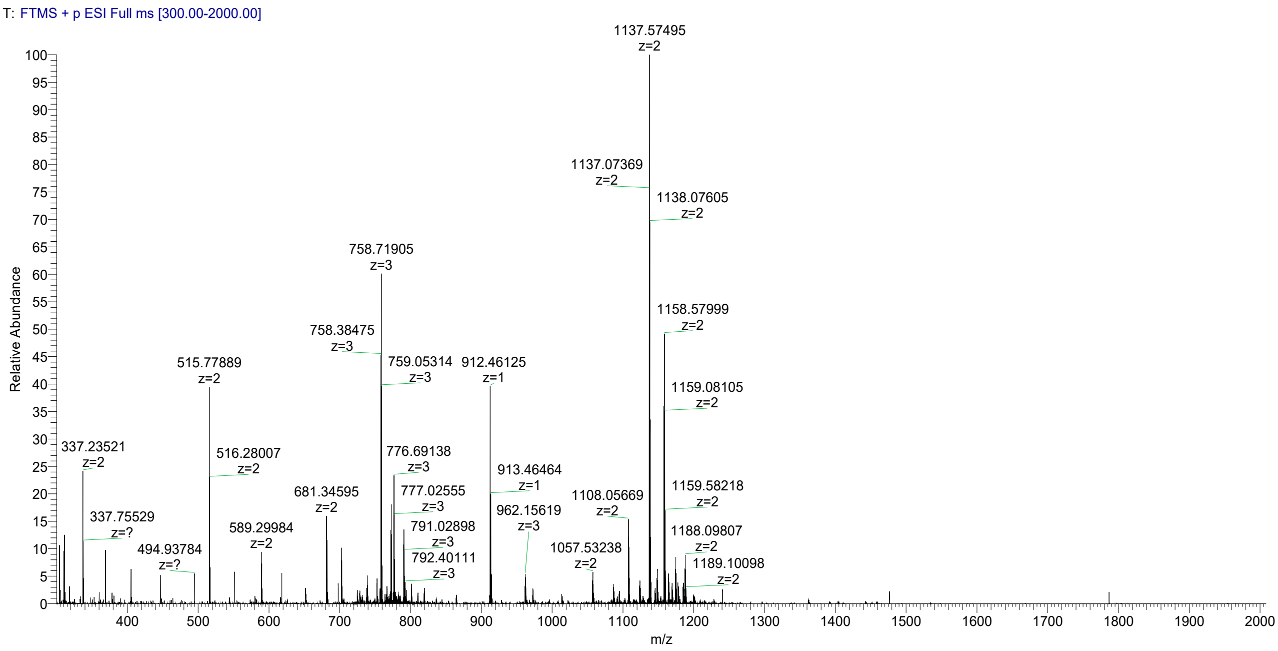
T: FTMS + p ESI Full ms [300.00-2000.00]
1137.57495 z=2
1137.07369 z=2
1138.07605 z=2
758.71905 z=3
1158.57999 z=2
758.38475 z=3
759.05314 z=3
912.46125 z=1
515.77889 z=2
1159.08105 z=2
337.23521 z=2
516.28007 z=2
776.69138 z=3
913.46464 z=1
681.34595 z=2
777.02555 z=3
1108.05669 z=2
1159.58218 z=2
337.75529 z=?
962.15619 z=3
791.02898 z=3
1188.09807 z=2
494.93784 z=?
589.29984 z=2
1057.53238 z=2
792.40111 z=3
1189.10098 z=2
Relative Abundance
m/z

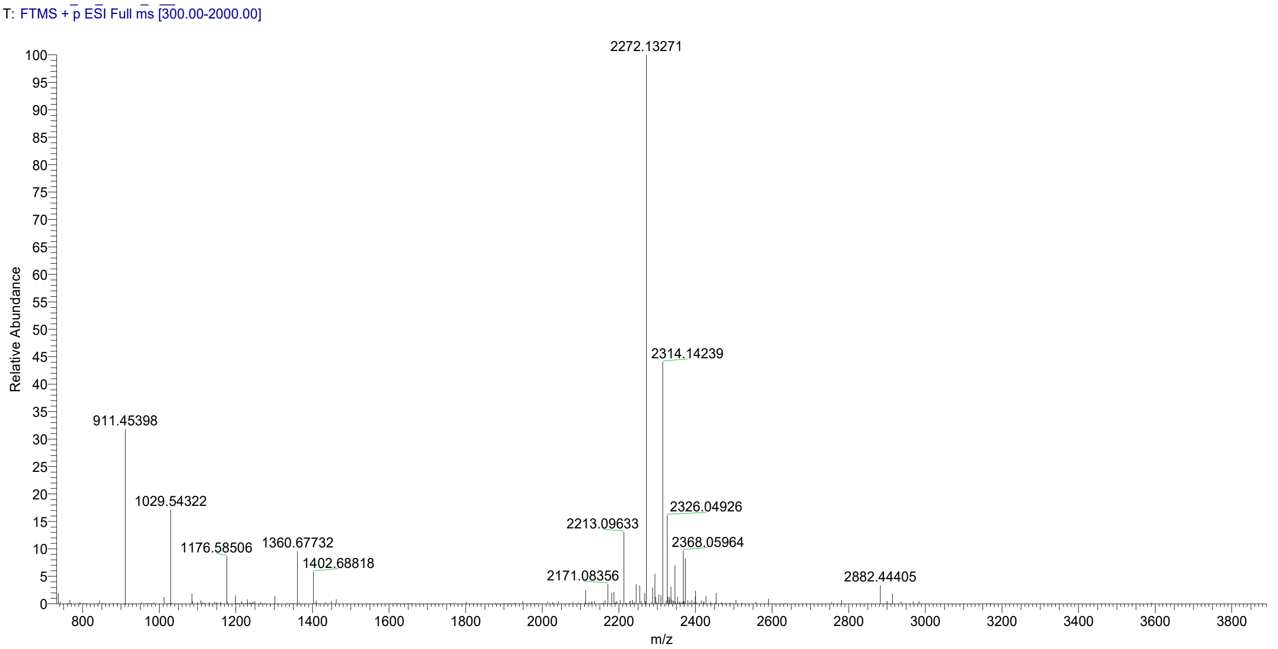
T: FTMS + p ESI Full ms [300.00-2000.00]
2272.13271
2314.14239
911.45398
1029.54322
2326.04926
2213.09633
1176.58506
1360.67732
2368.05964
1402.68818
2171.08356
2882.44405
Relative Abundance
m/z

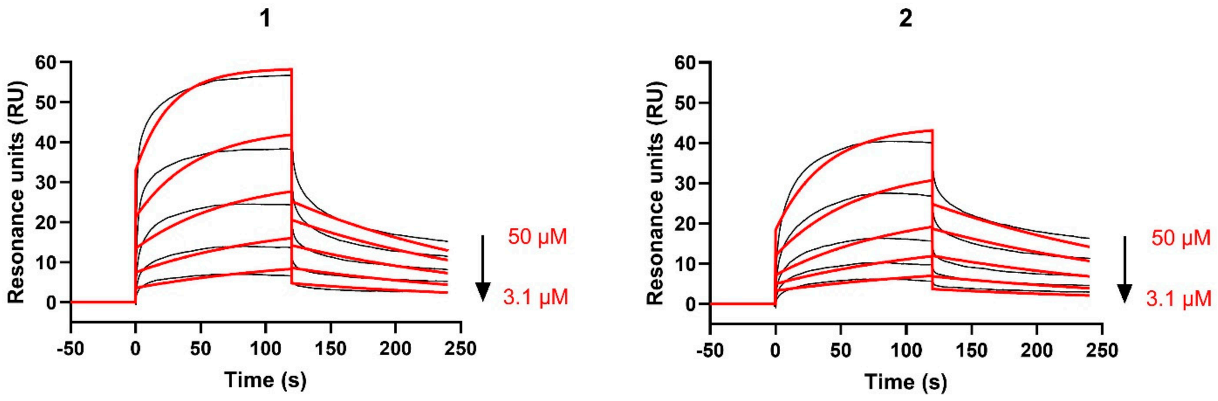

Figure S8. Surface plasmon resonance (SPR) analysis of PMX-Based Peptidomimetics 1 and 2. Representative SPR sensorgrams showing the binding kinetics for the monomeric PMs and immobilized RBD of Wuhan-Hu-1 spike protein (1:2 dilutions of peptidomimetics, starting from 50 µM). RBD was coupled to a Cytiva CM5 chip at 5350 RU. Data are shown as black lines, and the best fit of the data to a 1:1 binding model is shown in red. One representative sensorgram from 2 independent experiments is shown for each compound. See Table 2 in the main text for kinetics values.

Table S2. Antiviral and cytotoxicity properties of the synthesized peptidomimetics.

**Compound**	Concentration unit	U87.ACE2[a] **CC_{50}**	U87.ACE2[b] **20A.EU2** **IC_{50}**	A549.ACE2.TMPRSS2[+c] **CC_{50}**	A549.ACE2.TMPRSS2[+d] **Delta** **IC_{50}**
monomer **1**	µM	> 50	> 50	> 50	> 50
monomer **2**	µM	> 50	> 50	> 50	> 50
dimer **3**	µM	> 50	> 50	> 50	> 50
dimer **4**	µM	> 50	> 50	> 50	> 50
monomer **5**	µM	> 50	> 50	> 50	> 50
monomer **6**	µM	> 50	> 50	> 50	> 50
dimer **7**	µM	> 50	> 50	> 50	> 50
monomer **8**	µM	> 50	> 50	> 50	> 50
monomer **9**	µM	> 50	> 50	> 50	> 50
dimer **10**	µM	> 50	> 50	> 50	> 50
PMX	µg/ml	nd	nd	> 50	> 50
GS-441524	µM	nd	nd	> 40	2.3
remdesivir	µM	43	0.0044	nd	nd

[a]U87.ACE2 cells were incubated with a serial dilution of the compounds, incubated for 4 days and analyzed by MTS-PES. OD values were used to calculate the concentration that induced cell death by 50% (CC_{50}).

[b]U87.ACE2 cells were infected with SARS-CoV-2 (strain 20A.EU2) in absence or presence of the compounds, incubated for 4 days and analyzed by MTS-PES. OD values were used to calculate the % inhibition of viral replication. The median inhibitory concentration (IC_{50}), or the concentration that inhibited SARS-CoV-2-induced cell death by 50%, was calculated from the concentration–response curve.

[c]A549.ACE2.TMPRSS2$^+$ cells were incubated with a serial dilution of the compounds, incubated for 5 days and analyzed by MTS-PES. OD values were used to calculate the concentration that induced cell death by 50% (CC_{50}).

[d]A549.ACE2.TMPRSS2$^+$ cells were infected with SARS-CoV-2 (strain Delta) in absence or presence of the compounds, incubated for 5 days and analyzed by MTS-PES. OD values were used to calculate the % inhibition of viral replication. The median inhibitory concentration (IC_{50}), or the concentration that inhibited SARS-CoV-2-induced cell death by 50%, was calculated from the concentration–response curve.

nd: not determined
